# MCSP^+^ metastasis founder cells activate immunosuppression early in human melanoma metastatic colonization

**DOI:** 10.1038/s43018-025-00963-w

**Published:** 2025-05-16

**Authors:** Severin Guetter, Courtney König, Huiqin Koerkel-Qu, Aleksandra Markiewicz, Sebastian Scheitler, Marie Katzer, Mark Berneburg, Philipp Renner, Beatrix Cucuruz, Leonhard Guttenberger, Veronika Naimer, Kathrin Weidele, Steffi Treitschke, Christian Werno, Hanna Jaser, Tonia Bargmann, Armin Braun, Florian Weber, Katja Evert, Alexander Rochwarger, Christian M. Schürch, Katharina Limm, Peter J. Oefner, Reinhard Rachel, Felix Baumann, Jens Warfsmann, Lisa Schmidleithner, Kathrin Guetter, Parvaneh Mohammadi, Anja Ulmer, Sebastian Haferkamp, Christoph A. Klein, Melanie Werner-Klein

**Affiliations:** 1 Experimental Medicine and Therapy Research, University of Regensburg, Regensburg, Germany; 2 Department of Dermatology, University Medical Center Regensburg, Regensburg, Germany; 3 Department of Surgery, University Medical Center Regensburg, Regensburg, Germany; 4 Department of Vascular Surgery, University Medical Center Regensburg, Regensburg, Germany; 5 Division of Personalized Tumor Therapy, Fraunhofer Institute for Toxicology and Experimental Medicine, Regensburg, Germany; 6 Preclinical Pharmacology and Toxicology, Fraunhofer Institute for Toxicology and Experimental Medicine ITEM member of Biomedical Research in Endstage and Obstructive Lung Disease Hannover (BREATH) in the German Center for Lung Research (DZL), Hannover, Germany; 7 Institute of Immunology, Hannover Medical School, Hannover, Germany; 8 Institute of Pathology, University of Regensburg, Regensburg, Germany; 9 Department of Pathology and Neuropathology, University Hospital and Comprehensive Cancer Center Tübingen, Tübingen, Germany; 10 Cluster of Excellence iFIT (EXC 2180) “Image-Guided and Functionally Instructed Tumor Therapies”, University of Tübingen, Tübingen, Germany; 11 Institute of Functional Genomics, University of Regensburg, Regensburg, Germany; 12 Center for Electron Microscopy, University of Regensburg, Regensburg, Germany; 13 Department of Pharmaceutical Technology, University of Regensburg, Regensburg, Germany; 14 Leibniz Institute for Immunotherapy, University Hospital Regensburg, Regensburg, Germany; 15 Department of Dermatology, University of Tübingen, Tübingen, Germany

**Keywords:** Metastasis, Tumour immunology, Melanoma, Cancer

## Abstract

To investigate the early, poorly understood events driving metastatic progression, we searched for the earliest detectable disseminated cancer cells (DCCs), also often referred to as disseminated tumor cells (DTCs), in sentinel lymph node (SLN) biopsies of 492 patients with stage I–III melanoma. Using micromanipulator-assisted isolation of rare DCCs, single-cell mRNA and DNA sequencing, codetection by indexing immunofluorescence imaging and survival analysis, we identified melanoma-associated chondroitin sulfate proteoglycan (MCSP)^+^ melanoma cells as metastasis founder cells (MFCs). We found that DCCs entering SLNs predominantly exhibited a transitory phenotype that, upon interferon-γ exposure triggered by CD8 T cells, dedifferentiated into a neural-crest-like phenotype. This was accompanied by increased production of small extracellular vesicles (sEVs) carrying the immunomodulatory proteins CD155 and CD276 but rarely programmed cell death protein 1 ligand 1. The sEVs suppressed CD8 T cell proliferation and function, facilitating colony formation. Targeting MCSP^+^ MFCs or their immune escape mechanisms could be key to curing melanoma early by preventing manifestation of metastasis.

## Abstract (web summary)

Guetter et al. identify a melanoma-associated chondroitin sulfate proteoglycan-positive subpopulation of metastasis founder cells from lymph node biopsies of patients with melanoma and observe that they mediate immune evasion and predict systemic metastasis and poor outcomes.

## Main

Despite progress in immunotherapy of melanoma, over 40% of patients with metastasis succumb to their disease^1^. Early prevention of metastasis formation, therefore, is an important medical goal. At diagnosis, however, nearly 50% of melanomas, including those ≤1 mm thick (T1 stage), have spread regionally or to distant sites^2^. We previously found that most of these early disseminated cancer cells (DCCs) lack key genetic changes necessary for metastasis formation, which are acquired during cell divisions at metastatic sites^2^. This suggests that melanomas (and other cancers such as non-small cell lung cancer^3^) undergo molecular evolution at distant sites parallel to the primary tumor^4^. Yet, the metastasis founder cells (MFCs) and the molecular features driving this evolution are currently unknown.

A long-standing debate exists on whether migrating cancer stem cells (CSCs; that is, cancer cells with stem-like phenotypes) colonize distant sites. Early on, such cells were termed metastasis-initiating cells (MICs)^5–9^. The applicability of the CSC concept to MICs, however, remains controversial^10,11^, especially in melanoma, where studies have yielded conflicting results^12–16^ suggesting that melanoma stemness is not strictly hierarchical but results from cell plasticity that is hypothesized, although not proven, to underlie metastasis formation. These studies, however, focused on advanced stages of melanoma evolution, not early metastatic events. We aimed to investigate phenotypic progression in early metastasis, from initial homing to an ectopic site (here, the lymph node (LN)), through incipient colony formation and subsequent micrometastasis and macrometastasis formation, to identify and characterize MFCs without relying on CSC-based premises. To do so, we adapted our previously developed quantitative immunocytology (IC) assay^17^ to detect early DCCs using melanoma-associated chondroitin sulfate proteoglycan (MCSP), a cell surface marker chosen for both its ability to enable transcriptomic analysis and its expression in melanoma circulating tumor cells and DCCs with tumor-forming potential^2,18^. Additionally, MCSP’s role in stem-like cells across tissues and its association with CSCs in breast and head and neck cancers suggested that relevant cells were not overlooked^19,20^. Importantly, unlike previous single-cell RNA-sequencing (scRNA-seq) studies, we aimed to capture the earliest metastatic steps, from single invading melanoma cells to early micrometastatic colonies. High-throughput scRNA-seq approaches require target cell concentrations of at least 1 in 1,000 per sample, unachievable at early colonization when 1–200 DCCs per million LN cells are found. This rarity necessitated an alternative approach, combining microscopic inspection and micromanipulator-assisted manual isolation of individual candidate MFCs for scRNA-seq.

## Results

### MCSP as surface marker for melanoma DCC detection

**Fig. 1:**
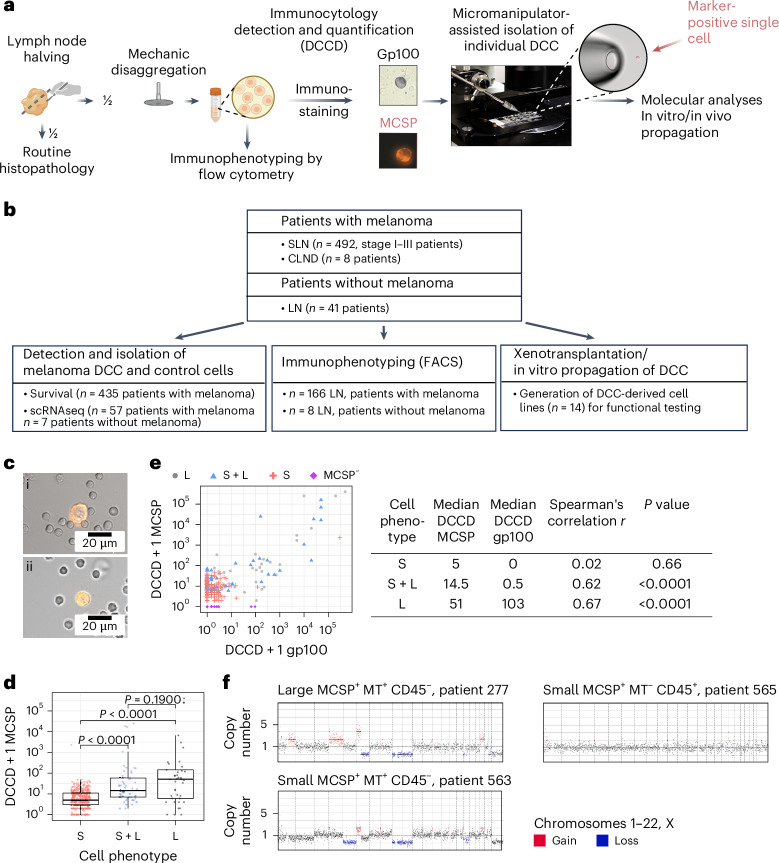
MCSP^+^ LN-derived cells comprise melanoma and nonmelanoma cells. **a**, LNs were split into halves for routine histopathology and IC. After mechanical disaggregation, single-cell suspensions were stained for gp100 or MCSP. Positive events were counted and recorded per million LN cells and isolated by micromanipulation. Isolated cells underwent single-cell molecular analyses. MCSP^+^ cells were propagated in vivo and in vitro. Immunophenotyping of LN cells was performed by flow cytometry if the number of leftover cells permitted. **b**, Overview of patients and methods. CLND, complete LN dissection. FACS, fluorescence-activated cell sorting. **c**, MCSP staining intensity and diameter of MCSP^+^ cells in SLNs of patients with melanoma with large, intensely stained cells (i) and small cells (ii). Scale bars are as indicated on the merged images of fluorescence and bright-field channels. **d**, DCCD_MCSP_ of LNs with MCSP^+^ cells (*n* = 477 SLNs from 392 patients) separated according to detected cellular phenotypes (diameter) into small (S; *n* = 378 SLNs), small and large (S + L; *n* = 58 SLNs) and large (L; *n* = 41 SLNs). **e**, Correlation of DCCD_MCSP_ and DCCD_gp100_ in SLNs stained for both MCSP and gp100 (*n* = 542 SLNs, 430 patients). Displayed are phenotypes of MCSP^+^ cells (S, *n* = 335 SLNs; S + L, *n* = 54 SLNs; L, *n* = 37 SLNs) and SLNs negative for MCSP (MCSP^−^ cells, *n* = 116 SLNs). **f**, Representative CNA profiles of small and large MCSP^+^MT^+^CD45^−^ and small MCSP^+^MT^−^CD45^+^ cells. *P* values in **d** were determined using a Wilcoxon test. *P* values in **e** were determined using Spearman’s rank correlation. Statistical tests were two-sided. Boxes mark the median, lower quartile and upper quartile, with whiskers extending to the minimum and maximum values within 1.5 times the interquartile range. Points beyond this range are shown as outliers. Source data

To track early metastatic colonization, we focused on LN samples because of their accessibility and their critical role in melanoma progression. LN status directly correlates with melanoma mortality^17^ and the lymphatic environment protects melanoma DCCs from ferroptosis, enhancing their survival during subsequent metastasis through the blood^21^. We exploited a quantitative assay to measure LN invasion, that is gp100 staining of melanoma DCCs in disaggregated sentinel LNs (SLNs) (Fig. 1a)^17^. This method quantifies gp100^+^ cells per million LN cells, expressed as the DCC density (DCCD). This assay (1) provided the best accuracy for DCC detection as a single-marker assay^18^; (2) demonstrated that every detected cancer cell increases the melanoma mortality risk^17^; (3) established that a DCCD around 100 marks microscopic metastasis formation^2^; and (4) revealed specific genetic alterations acquired at this metastatic colonization stage and DCCD^2^. However, gp100 as an intracellular marker precludes viable cell isolation for transcriptomic analysis. We, therefore, used the gp100 assay as a reference to rank phenotypic progression and microenvironmental responses during early metastasis formation and compared it to melanoma cell detection using MCSP.

**Extended Data Fig. 1:**
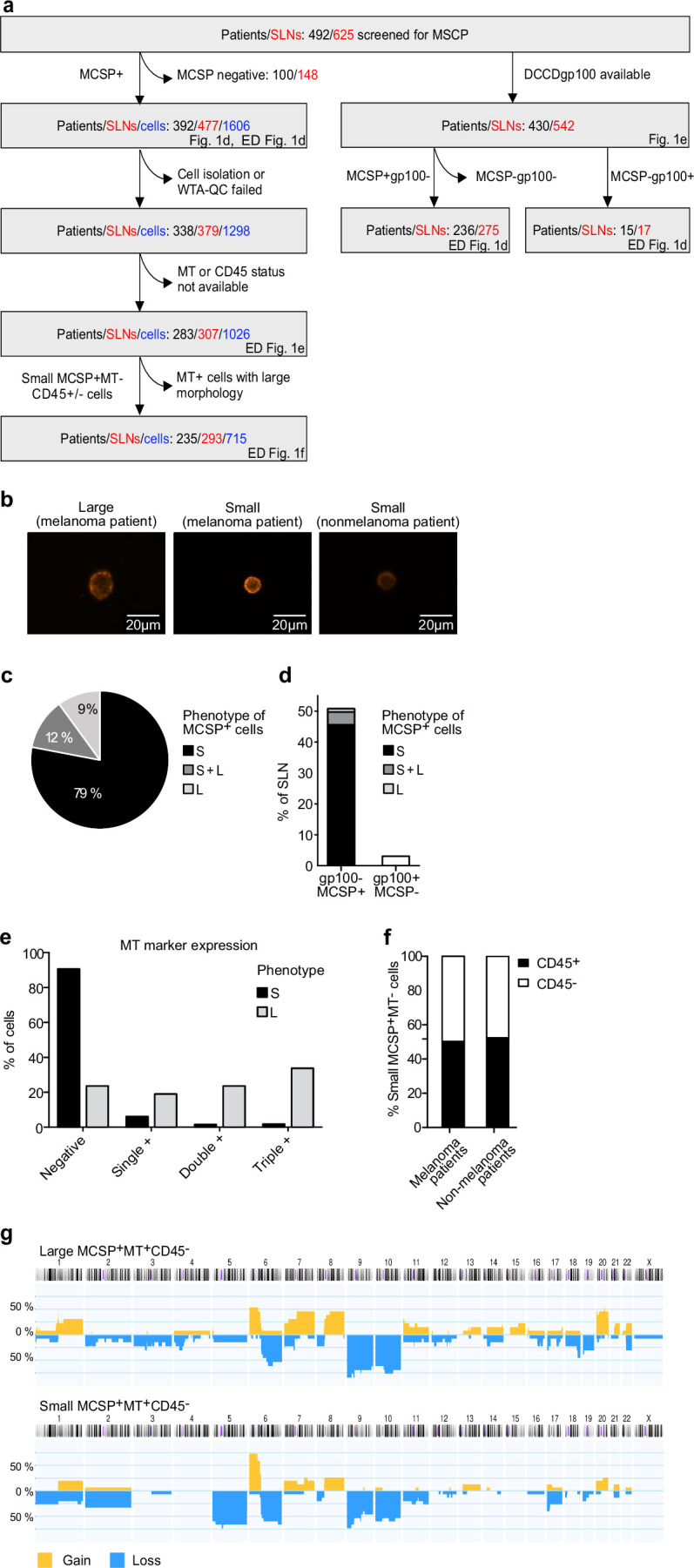
Combining MCSP immunofluorescence with melanoma transcript and genome analysis identifies melanoma DCCs. **a**, Schematic overview of analyzed patient samples, drop-out causes and reference to individual figure panels. Number of patients, SLNs and cells are depicted in black, red and blue, respectively. **b**, Staining intensity of MCSP^+^ cells in SLNs and LNs of melanoma and nonmelanoma patients. Scale bar as indicated. See also Fig. 1c for merged images of fluorescence and bright field channels of MCSP^+^ cells in SLNs of melanoma patients. **c**, Percentage of MCSP^+^ SLNs of melanoma patients with MCSP^+^ cells separated according to their phenotype (diameter) into small (S), small and large (S + L), large (L) cells. SLN numbers see panel a) and main text. **d**, Percentage of gp100^-^MCSP^+^ (n = 275) and gp100^+^MCSP^-^ SLN (n = 17) among SLNs of melanoma patients with staining-results for both gp100 and MCSP (n = 542). gp100^-^MCSP^+^ SLNs are annotated according to the observed phenotype (diameter) of detected MCSP^+^ cells. **e**, Percentage of MCSP^+^ small (n = 789) and large (n = 237) cells negative or positive for MT expression (*gp100, DCT* and *MLANA*), either alone (single^+^) or in combination (double^+^ for any two markers or triple^+^). **f**, Percentage of MCSP^+^MT^-^ cells with or without *CD45* expression isolated from melanoma (MT^-^ small cells, n = 715) and nonmelanoma patients (MT^-^ small cells, n = 61), MT = melanoma transcripts. **g**, Cumulative frequency plots of genomic aberrations in MCSP^+^MT^+^CD45^-^ large (n = 13) and small (n = 15) cells with genomic gains and losses in orange and blue, respectively. Source data

Staining for MCSP (*CSPG4*) in 625 SLNs from 492 patients with early-stage melanoma (Fig. 1a,b and Extended Data Fig. 1a) identified two morphologically distinct MCSP^+^ cell populations: large cells with a diameter of about 20 µm and bright fluorescence and small cells with a diameter of half the size (10 µm) and often weaker fluorescence staining (Fig. 1c and Extended Data Fig. 1b). MCSP^+^ SLNs (*n* = 477) contained small cells more frequently (79%, 378/477) than a mixture of small and large cells (12%, 58/477) or large cells only (9%, 41/477) (Extended Data Fig. 1c). The median DCCD_MCSP_ increased with large cell presence (Fig. 1d; *P* < 0.0001, Wilcoxon test). Direct comparison of the DCCD_gp100_ and DCCD_MCSP_ from the same SLN (*n* = 542) showed significant correlation only for samples with large MCSP^+^ cells (Fig. 1e; *P* < 0.0001, Spearman’s correlation). Notably, 51% (275/542) of gp100^−^ SLNs harbored MCSP^+^ cells, while only 3% (17/542) of MCSP^−^ SLN were positive for gp100 (Extended Data Fig. 1d). This discrepancy, primarily arising from cells with small cell morphology, and the fact that we also found small MCSP^+^ cells in 85% (35/41) of patients without melanoma (Extended Data Fig. 1b), albeit at low frequency (median DCCD = 4, range 0–15), led us to the question whether all small cells are indeed melanoma cells.

### MCSP staining and melanoma transcript detection identify DCCs

To investigate this further, we isolated 1,606 MCSP^+^ cells from 477 SLNs of 392 patients with melanoma, performed whole-transcriptome amplification (WTA)^22,23^ and tested 1,026 high-quality single cells (Methods) for the melanoma transcripts (MT) *PMEL* (gp100), *DCT* (dopachrome tautomerase) and *MLANA* (Melan A) (Extended Data Fig. 1a). Large MCSP^+^ cells (*n* = 237) predominantly expressed all three MTs, indicating melanoma origin (Extended Data Fig. 1e). Among small MCSP^+^ cells, 9% (74/789) expressed at least one MT, confirming a melanocytic origin.

We then tested the small MCSP^+^MT^−^ cells from both patients with melanoma (*n* = 715 cells) and patients without melanoma (*n* = 61 cells) for the leukocyte marker CD45 and found that 50% (360/715) and 53% (32/61), respectively, expressed *CD45* (Extended Data Fig. 1f). In total, 9% (74/789) of small MCSP^+^ cells were putative melanoma cells (Extended Data Fig. 1e), 46% (360/789) were CD45^+^ lymphocytes and 45% (355/789) were of unknown nonmelanoma lineage (Extended Data Fig. 1f).

**Extended Data Fig. 2:**
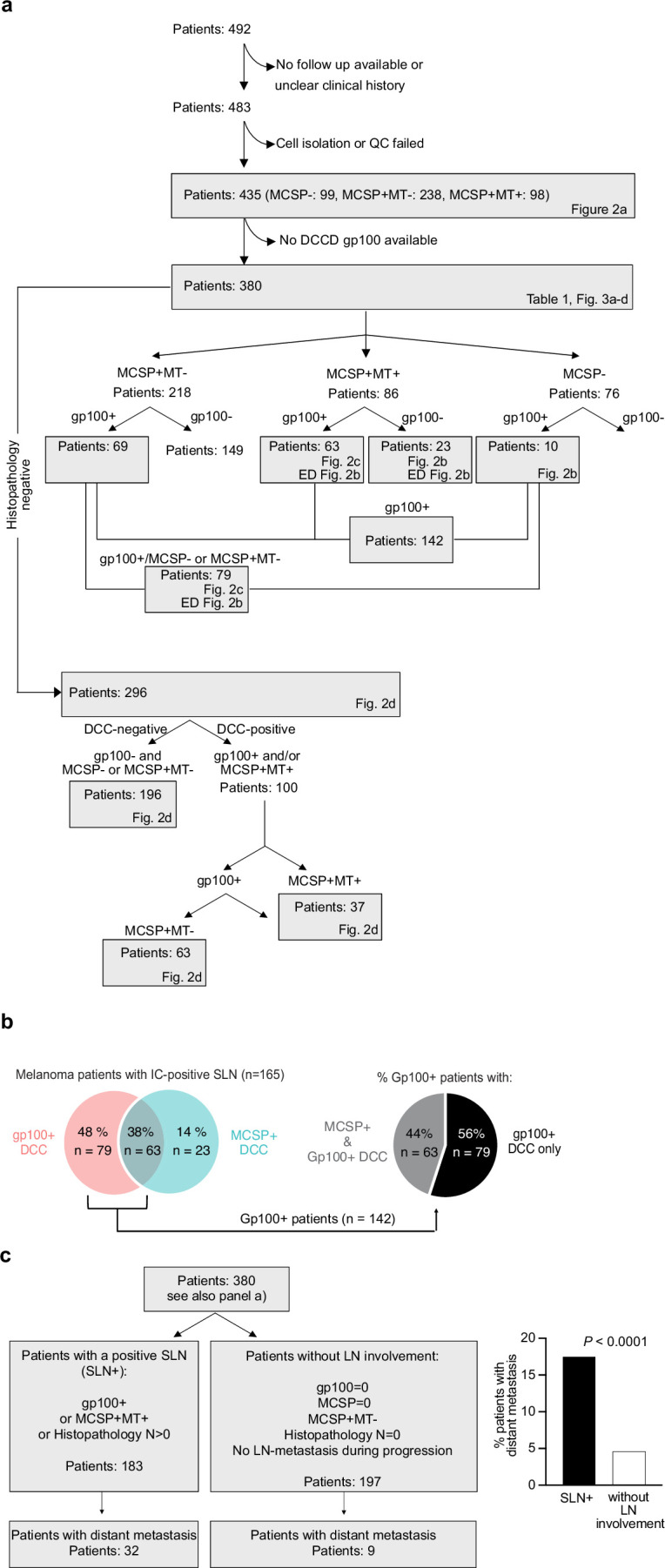
MCSP defines a subset of melanoma DCC linked to systemic progression. **a**, Schematic overview of analyzed patient samples and exclusion criteria. Note reference to individual figure panels. **b**, left panel: Melanoma patients with IC positive SLNs (n = 165). Percentage and number of patients with SLNs single-positive for gp100^+^ DCCs (79/165) or MCSP^+^ DCCs (23/165) or double-positive for gp100^+^ DCCs and MCSP^+^ DCCs (63/165). Right panel: Percentage and number of patients with Gp100^+^ DCCs in their SLNs, in whom MCSP^+^ DCCs were co-detected or not. **c**, Impact of LN involvement at diagnosis on metachronous distant metastasis. SLN^+^: patients with gp100^+^ and/or MCSP^+^MT^+^ cells in the SLN or with a positive SLN by histopathology. Patients without LN involvement: patients lacking gp100^+^ or MCSP^+^MT^+^ cells in SLN, a negative SLN by histopathology and without evidence of LN involvement at any time during the disease. *P* values in **c**, Fisher’s exact test (two-sided). See Table 1 for baseline characteristics of the study cohort. Source data

Copy-number alterations (CNAs) have been shown to differentiate not only between normal and malignant cells but also between malignant melanoma cells and rare, benign LN-residing nevus cells^12,24,25^. We analyzed CNAs in small (*n* = 15) and large (*n* = 13) MCSP^*+*^MT^*+*^CD45^−^ and MCSP^+^MT^−^CD45^+^ (*n* = 15) cells after whole-genome amplification (WGA) of their single-cell DNA^23^. None of the small MCSP^+^MT^−^CD45^+^ cells harbored CNAs, while all small (*n* = 15) and large (*n* = 13) MCSP^+^MT^+^CD45^−^ cells did (Fig. 1f). The CNA profiles of small and large MCSP^+^MT^+^CD45^−^ cells were highly similar (Extended Data Fig. 1g). Thus, genomic analysis confirmed the malignant origin of both small and large MCSP^+^MT^+^ cells. A comparison of SLNs from patients with both MCSP^+^MT^+^ and gp100^+^ status (*n* = 380; Extended Data Fig. 2a) revealed a significantly higher positivity rate for gp100 (37%, 142/380) than for MCSP and MT (23%, 86/380; *P* < 0.0001, Fisher’s exact test). This difference prompted us to investigate the prognostic impact of MCSP^+^MT^+^ cells, hereafter termed MCSP^+^ DCCs, on progression-free (PFS), melanoma-specific (MSS) and overall survival (OS).

### MCSP^+^MT^+^ DCCs predict poor outcome

**Fig. 2:**
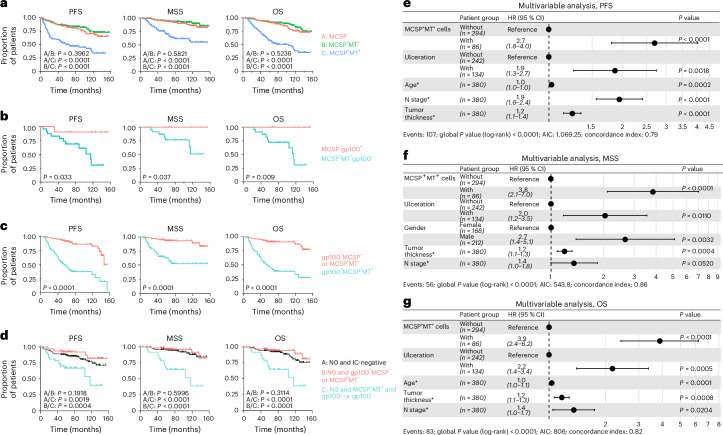
MCSP^+^ DCCs impose high risk of progression. **a**–**d**, Kaplan–Meier curves of PFS, MSS and OS of patients stratified according to IC, MT assay or histopathology results. **a**, Patients with LNs without MCSP cells (MCSP^−^, *n* = 99), positive for MCSP^+^MT^−^ cells (*n* = 238) or positive for MCSP^+^MT^+^ cells (*n* = 98). **b**, Patients with MCSP^−^gp100^+^ LNs (*n* = 10) or MCSP^+^MT^+^gp100^−^ LNs (*n* = 23) (b). **c**, Patients with gp100^+^ cells (*n* = 142) stratified according to whether MCSP^+^ DCCs (gp100^+^MCSP^+^MT^+^, *n* = 63) were codetected in the SLN (gp100^+^MCSP^−^ or MCSP^+^MT^−^, *n* = 79). **d**, Patients with histopathology-negative SLNs (N0, *n* = 296) stratified according to whether IC was negative for MCSP^+^MT^+^ and gp100^+^ DCCs (N0 and IC-negative, *n* = 196), positive for gp100^+^ DCCs only (N0 and gp100^+^MCSP^−^ or MCSP^+^MT^−^, *n* = 63) or positive for MCSP^+^MT^+^ DCCs with or without codetection of gp100^+^ DCCs (N0, MCSP^+^MT^+^ and gp100^+^ or gp100^−^, *n* = 37). **e**–**g**, Multivariable Cox regression analysis for PFS (**e**), MSS (**f**) and OS (**g**) comprising the most informative, backward selected features (*n* = 380). Patients without MCSP^+^MT^+^ cells, female patients and patients without ulceration were used as the reference for defining the hazard ratio (HR). Parameters marked with an asterisk (*) were analyzed as continuous variables, that is, the increase in age (in year), N category (N status) and thickness (in mm). The dots represent the HRs and the whiskers indicate the 95% confidence interval (CI). AIC, Akaike information criterion. *P* values in **a**–**d** were determined using a log-rank test. *P* values in **e**,**f** were determined using a Wald test. All statistical tests were two-sided. The baseline characteristics of patients are listed in Table 1. Source data

We compared three groups: patients with SLNs (1) negative for MCSP-expressing cells (*n* = 99); (2) positive for MCSP^+^MT^−^ cells (*n* = 238) and (3) positive for MCSP^+^MT^+^ cells (*n* = 98), totaling 435 patients with follow-up data for PFS, MSS and OS (Extended Data Fig. 2a and Table 1). The strongest impact on all endpoints was found for MCSP^+^MT^+^ cases (*P* < 0.0001, log-rank test; Fig. 2a). In contrast, MCSP^+^MT^−^ patients showed nearly identical outcomes to MCSP^−^ patients, consistent with the classification of MCSP^+^MT^−^ cells as nonmalignant cells. Our standardized protocol for isolation of MCSP^+^ cells for RNA analysis limits isolation to 5–10 cells, such that MT status is unavailable for additionally detected and counted but not isolated MCSP^+^ cells. Therefore, the DCCD_MCSP_ overestimates the number of MCSP^+^MT^+^ cells (because DCCD_MCSP_ = DCCD_MCSP+MT+_ + DCCD_MCSP+MT−_), particularly when more than ten cells are detected. We could, however, directly compare how single positivity (that is, for either gp100^+^ or MCSP^+^MT^+^ cells within a sample) impacts survival. Of 380 patients with both gp100 and MCSP data, 23 had only MCSP^+^MT^+^ cells and 10 had gp100^+^ cells but no MCSP^+^MT^+^ cells (Extended Data Fig. 2a). Strikingly, patients with only MCSP^+^MT^+^ cells had a significantly shorter survival (Fig. 2b; *P* = 0.033 for PFS, *P* = 0.037 for MSS and *P* = 0.009 for OS, log-rank test) than MCSP^−^ patients with gp100^+^ cells. The prognostic importance of MCSP^+^ DCCs was further supported by comparing patients with gp100^+^ DCCs with and without MCSP^+^ DCCs, showing worse outcomes for those with MCSP^+^ DCCs (*P* < 0.0001; Fig. 2c). Furthermore, in N0 patients with tumor-free LNs according to histopathology (Extended Data Fig. 2a), who are often positive in IC^17^, detection of MCSP^+^ DCCs placed patients at high risk for all endpoints (Fig. 2d).

**Table 1:** Patient characteristics

Characteristic	Number of patients	Percentage	Median	Range	Interquartile range
Patients	435				
**Gender**					
Female	192	44.1			
Male	243	55.9			
Age (years)			56	15–85	
Breslow’s thickness (mm)			2	0.2–15.0	1–3
**Ulceration**					
No	278	63.9			
Yes	153	35.2			
Not specified	4	0.9			
**Localization**					
Extremities	252	57.9			
Trunk or head	183	42.1			
**Nodal status histopathology**					
Negative	342	78.6			
Positive	93	21.4			
**Clinical stage**					
IA	49	11.3			
IB	168	38.6			
II	1	0.2			
IIA	73	16.8			
IIB	40	9.2			
IIC	12	2.8			
III	4	0.9			
IIIA	32	7.4			
IIIB	38	8.7			
IIIC	17	3.9			
IIID	1	0.2			
DCCD^a^ (MCSP)			5	0–400,000	1–12
DCCD^b^ (gp100)			0	0–500,000	0–1
**Survival**					
Deceased	98	22.5			
Alive	337	77.5			
**Adjuvant therapy**					
Yes^c^	54	12.4			
No/not specified	381	87.6			

^a^DCCD in the SLN: number of MCSP^+^ cells per million isolated cells; if more than one node per patient was positive, the node with the highest cancer cell density was taken.

^b^DCCD in the SLN: number of gp100^+^ cells per million isolated cells; if more than one node per patient was positive, the node with the highest cancer cell density was taken.

^c^Of the 54 patients, three received pembrolizumab, 47 received IFNG and four received unspecified therapy.

In summary, using only gp100 as detection marker fails to detect melanoma DCCs with metastasis founder potential in about 14% (23/165) of patients with evidence of early dissemination (Extended Data Fig. 2a,b). Moreover, exclusive gp100 positivity overestimates the risk for poor PFS, MSS and OS in 56% (79/142) of gp100^+^ patients, as they lack MCSP^+^MT^+^ DCCs (Extended Data Fig. 2a,b). This highlights the strong independent risk posed by MCSP^+^MT^+^ DCCs, prompting us to assess the impact of DCCD_MCSP_ and MT status alongside clinical risk factors such as Breslow thickness, N status, ulceration, gp100, sex and age. Univariable analysis showed that gp100 counts, MCSP counts and MT expression in MCSP^+^ cells significantly impacted PFS, MSS and OS (all *P* < 0.0001, Wald test; Table 2). However, multivariable analysis revealed that detection of MCSP^+^MT^+^ melanoma cells in the SLN, alongside primary melanoma thickness, was the strongest risk factor for PFS, MSS and OS, outperforming all other variables, including DCCD_gp100_ (Fig. 2e–g). Given their detection in gp100^−^ samples and impact on patient survival, small and large MCSP^+^MT^+^ cells may comprise early harbingers of metachronous metastasis and death in malignant melanoma. Because patients with cutaneous melanoma do not die from LN metastasis but from distant metastasis, we directly evaluated the risk of metachronous distant metastasis for patients with locoregional lymphatic spread at diagnosis or any time point thereafter. Patients with SLN involvement by either histopathology or IC (gp100^+^ and/or MCSP^+^MT^+^) showed a fourfold higher frequency of distant metastasis than those without local LN involvement (Extended Data Fig. 2c; *P* < 0.0001). Most patients with distant metastasis had lymphatic involvement (78%, 32/41), while only 22% (9/41) had exclusive hematogenous spread.

**Table 2:** MCSP^+^MT^+^ counts strongly impact PFS, MSS and OS

	PFS		MSS		OS	
	HR (95% CI for HR)	*P* value	HR (95% CI for HR)	*P* value	HR (95% CI for HR)	*P* value
MCSP^+^MT^+^ cells (HR provided for patients with versus patients without)	4.3 (2.9–6.3)	<0.0001	5.7 (3.4–9.7)	<0.0001	6 (3.9–9.2)	<0.0001
log_10_ DCCD_gp100_	1.6 (1.4–1.8)	<0.0001	1.5 (1.3–1.8)	<0.0001	1.5 (1.4–1.7)	<0.0001
log_10_ DCCD_MCSP_	1.8 (1.5–2)	<0.0001	1.6 (1.4–2)	<0.0001	1.6 (1.4–1.9)	<0.0001
Gender (f, m)	1.8 (1.2–2.7)	0.0029	2.9 (1.6–5.4)	0.0007	1.9 (1.2–2.9)	0.0074
N status^a^	2.3 (1.9–2.8)	<0.0001	2.1 (1.6–2.6)	<0.0001	2 (1.6–2.4)	<0.0001
Age^a^	1 (1–1)	<0.0001	1 (1–1)	0.0014	1 (1–1.1)	<0.0001
Ulceration (HR provided for patients with ulcerated melanoma versus patients without)	2.7 (1.8–3.9)	<0.0001	3.1 (1.8–5.3)	<0.0001	2.9 (1.9–4.5)	<0.0001
Tumor thickness^a^	1.3 (1.2–1.4)	<0.0001	1.3 (1.2–1.4)	<0.0001	1.2 (1.2–1.3)	<0.0001

Univariable analysis of 380 patients (Extended Data Fig. 2a). *P* values were determined using a Wald test (two-sided).

^a^Analyzed as a continuous variable increasing per log level (log_10_ DCCD_gp100_ or DCCD_MCSP_): N category (N status), age (in years) and thickness (in mm). For example, each millimeter increase in thickness increases the HR for PFS by 1.3.

Source data

### DCCs switch phenotypes during metastatic colonization

**Extended Data Fig. 3:**
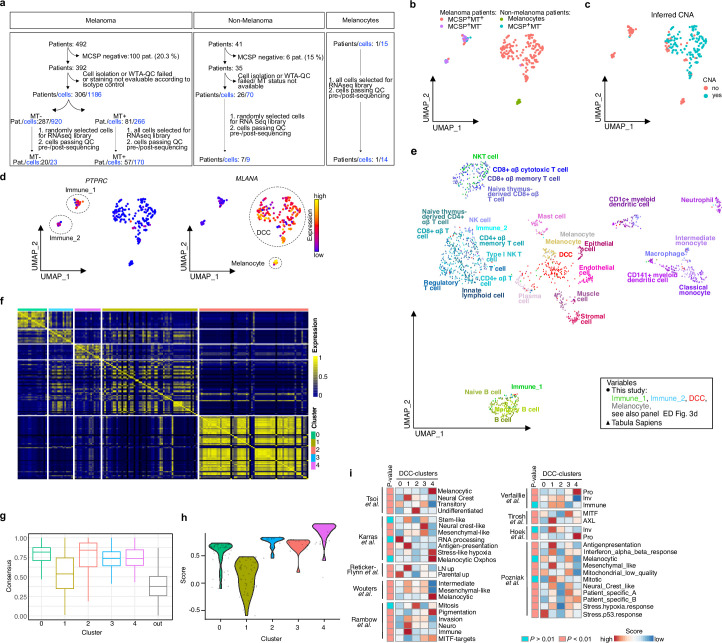
MCSP+ melanoma DCC display different phenotypes during metastatic colonization. **a**, Schematic overview of analyzed patient samples and exclusion criteria. The number of patients and cells are depicted in black and blue, respectively. **b-d**, UMAP of scRNA-seq data of LN-derived small and large MCSP^+^MT^+^ cells (n = 170 cells) and MCSP^+^MT^*-*^ cells (n = 23 cells) from melanoma patients, LN-derived MCSP^+^ cells from nonmelanoma patients (n = 9 cells) and cultured human melanocytes (n = 14 cells) with Seurat. Cells are annotated by their patient origin (melanoma, nonmelanoma patient) and cell type (melanocyte, MCSP/MT-status) (**b**), gene expression inferred CNA (**c**) or expression of marker genes for immune cells (*PTPRC/CD45*) and cells of melanocytic origin (*MLANA*) (**d**). **e**, scRNA-seq data (see panel **d**) integrated into skin and LN of the Human Cell Atlas (Tabula sapiens). Each cell type retrieved from the Human Cell Atlas (n = 33 cell types) was downsampled to contain 50 cells. Each cell is colored by its assigned cluster or cell type. **f, g**, Stability of DCC cluster assignment (Fig. 3a, n = 164 cells) using Seurat was assessed by comparison with multiple alternative clustering methods and variable parameter combination. Consensus heatmap (**f**) and its quantification (**g**). The box plots (**g**) show consensus scores of cell pairs within Seurat clusters and consensus scores of cell pairs between different Seurat clusters. Boxes represent the median, lower quartile, and upper quartile, with whiskers extending to the minimum and maximum values within 1.5 times the interquartile range. **h**, Silhoutte score analysis for DCC clusters. **i**, AUCell scores based on published melanoma subtype marker gene sets ^26–34^ averaged over DCC clusters. Source data

To investigate the molecular characteristics of candidate MFCs, we performed scRNA-seq on LN-derived small and large MCSP^+^MT^+^ cells (*n* = 170) and MCSP^+^MT^−^ cells (*n* = 23) from 77 patients with melanoma and MCSP^+^MT^−^ cells (*n* = 9) from 7 patients without melanoma. These cell numbers were obtained after screening of 492 patients with melanoma and 41 patients without melanoma, manual isolation of 1,606 single cells and application of stringent quality controls (Extended Data Figs. 1a and 3a). We also tested 14 cultured human melanocytes from one donor (Extended Data Fig. 3a). Expression-based dimensionality reduction separated DCCs (MCSP^+^MT^+^), nonmelanoma cells (MCSP^+^MT^−^) and melanocytes (Extended Data Fig. 3b). Transcript-based CNA analysis and expression of CD45 (*PTPRC*) and Melan A (*MLANA*) largely matched uniform manifold approximation and projection (UMAP) dimensionality reduction (Extended Data Fig. 3c,d); the DCC group comprised 139/164 (85%) cells with inferred genomic aberrations, whereas melanocytes or immune cells had none (0/14) or only 5% (2/38), respectively. Integration of the DCC group into the Human Cell Atlas confirmed their relatedness with melanocytic cells (Extended Data Fig. 3e).

**Fig. 3:**
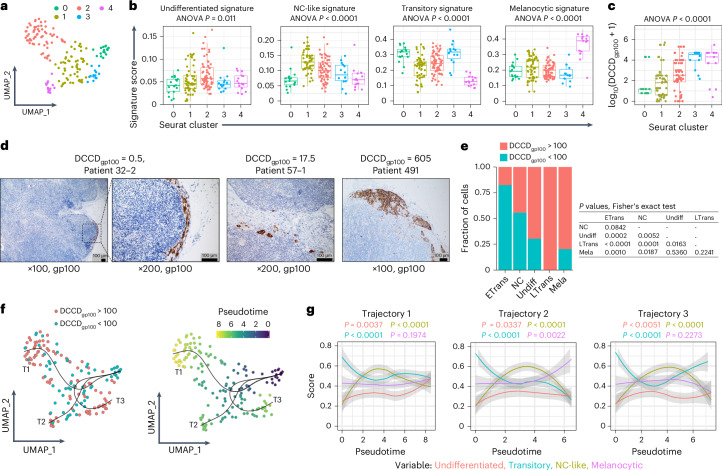
Metastatic colonization is associated with phenotypic plasticity of melanoma DCCs. **a**–**c**, scRNA-seq data from the DCC group (*n* = 164 cells; Extended Data Fig. 3d). Each point represents an individual cell. The clusters are labeled 0, 1, 2, 3 and 4, with corresponding cell numbers of *n* = 17, 55, 63, 14 and 15, respectively. Louvain clustering with Seurat based on UMAP Seurat (**a**). Signature scores of the four melanoma phenotypes^29^ for DCCs annotated by Seurat cluster labels (**b**). DCCD_gp100_ annotated by Seurat cluster label (**c**). **d**, Representative examples of SLNs analyzed by histopathology using gp100 staining. The results of the matched LN half analyzed by IC are provided as DCCD. The examples illustrate that a DCCD_gp100_ < 100 in IC corresponds to isolated tumor cells in histopathology, while a DCCD_gp100_ > 100 corresponds to micrometastasis^2^. **e**, Percentage of DCCs (*n* = 164 cells) before (DCCD_gp100_ < 100, *n* = 67 cells) and after (DCCD_gp100_ > 100, *n* = 97 cells) metastatic colony formation displaying the different phenotypes. **f**, Inferred trajectories (T1, T2 and T3) with slingshot. Left: each cell is colored according to its DCCD (DCCD_gp100_ < 100, blue, *n* = 67 cells; DCCD_gp100_ > 100, red, *n* = 97 cells). Right: each cell is colored according to its pseudotime. **g**, Melanocytic, NC-like, transitory and undifferentiated signature scores^29^ with AUCell score along pseudotime (slingshot) of trajectories 1–3. The gray areas indicate the 95% CI of the curves. *P* values in **b**,**c** were determined using a one-way ANOVA. All statistical tests were two-sided. Boxes mark the median, lower quartile and upper quartile, with whiskers extending to the minimum and maximum values within 1.5 times the interquartile range. Points beyond this range are shown as outliers. *P* values in **e** were determined using a Fisher’s exact test. *P* values in **g** were determined using a chi-square test. Source data

**Extended Data Fig. 4:**
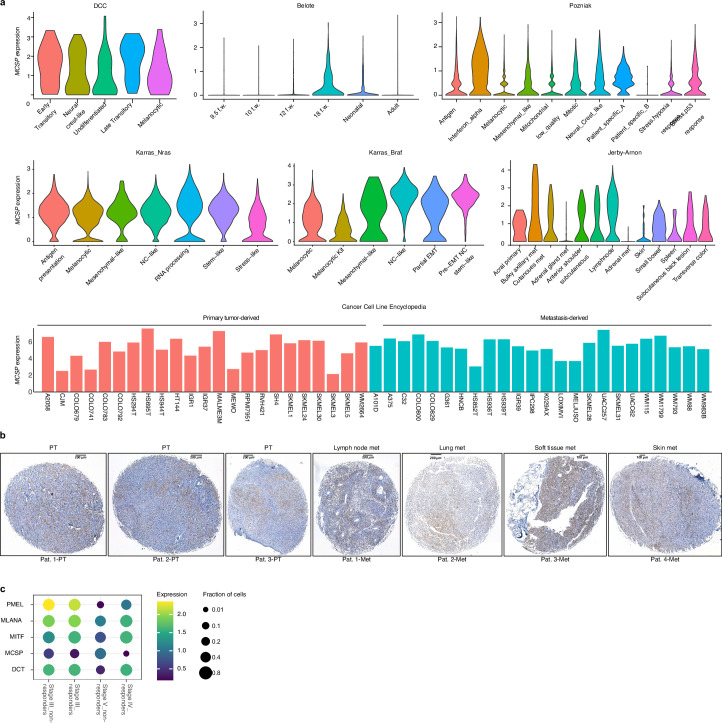
MCSP expression is preserved during metastatic progression of melanoma. **a**, *MCSP* expression in the five DCC clusters and published scRNA-seq data sets ^31,33,35,36^ or in melanoma cell lines of the Cancer Cell Line Encyclopedia^37^. **b**, Representative examples of primary melanomas (PT) and metastases (met) analyzed by histopathology using MCSP staining. **c**, Expression of *DCT*, *MCSP*, *MITF*, *PMEL*, *MLANA* in relation to immunotherapy response in stage III/IV melanoma patients^31^. Source data

Graph-based cluster analysis of the DCC group revealed five transcriptomic clusters (Fig. 3a) validated by multiple reduction and clustering methods (Extended Data Fig. 3f–h). All clusters aligned with melanoma transcriptional signatures from human^26–32^ and mouse^33,34^ datasets (Extended Data Fig. 3i). Notably, *MCSP* expression was detected across all clusters and in human fetal epidermal melanocytes^35^, *NRAS* or *BRAF*-mutant mouse melanoma models^33^, human primary tumors, metastases^31,36^ and melanoma cell lines^37^ (Extended Data Fig. 4a). Immunohistochemistry (IHC) of human primary melanomas and metastases showed that MCSP was heterogeneously expressed in 60% of metastases (25/42) and 62% of primary tumors (8/13; Extended Data Fig. 4b). Additionally, *MCSP* expression was higher and more frequent in immunotherapy nonresponders than in responders^31^, unlike *DCT*, *MITF*, *MLANA* and *PMEL* (gp100; Extended Data Fig. 4c). Together, these findings further support the use of MCSP as an MFC detection marker.

For detailed characterization of the five transcriptomic DCC-derived clusters, we applied the signatures from Tsoi et al.^29^, originally developed from human embryonic stem cell differentiation into melanocytes. Each DCC cluster was significantly associated with one of the transcriptomic subtypes: transitory with clusters 0 and 3, neural crest (NC)-like with cluster 1, melanocytic with cluster 4 (all *P* < 0.001) and undifferentiated with cluster 2 (*P* = 0.011, analysis of variance (ANOVA); Fig. 3b and Extended Data Fig. 3i). Strikingly, cluster annotation was associated with DCCD_gp100_ values (*P* < 0.0001, ANOVA; Fig. 3c), with median DCCD_gp100_ values rising from 15 (cluster 0, transitory), through 61 (cluster 1, NC-like), 334 (cluster 2, undifferentiated) and 20,000 (cluster 3, transitory), to 37,500 (cluster 4, melanocytic). Clusters 0 and 3 comprising cells with a transitory phenotype could thereby be differentiated into early transitory (cluster 0, low DCCD, early invading DCCs) and late transitory cells (cluster 3, high DCCD, colony-forming DCC). Together, this strongly indicates that melanoma cells dynamically switch their phenotype during LN colonization.

**Extended Data Fig. 5:**
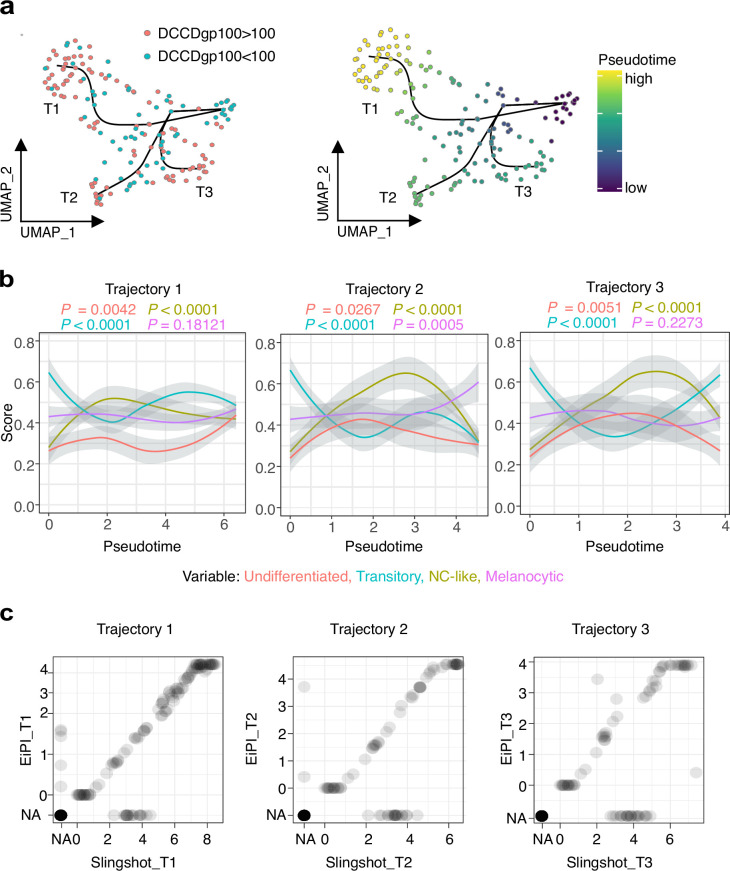
Robust trajectories of phenotype changes can be identified from low to high DCCD. **a**, Inferred trajectories (T1, T2, T3) with ElPiGraph. Left: each cell is colored according to its DCCD (DCCD < gp100 blue, n = 67 cells, DCCD > gp100 red, n = 97 cells). Right: each cell is colored according to its pseudotime. **b**, Melanocytic, neural-crest-like, transitory and undifferentiated signature scores^29^ with AUCell along pseudotime (EiPl) of trajectory 1-3. The grey area indicates the 95% confidence interval of the curves. *P* values according to Chi-square test (two-sided). **c**, Comparison of inferred pseudotime using Slingshot (x-axis) and ElPiGraph (y-axis) for trajectory 1-3 (T1, T2, T3). Each dot is a cell. NA indicates cells not belonging to the compared trajectories. Source data

We previously documented that a DCCD_gp100_ of around 100 marks the transition from isolated tumor cells to detectable colony formation as assessed by histopathology^2,18^ (Fig. 3d). Interestingly, early transitory and NC-like phenotypes dominated DCCD_gp100_ values < 100, while undifferentiated, late transitory and melanocytic phenotypes were mostly found at DCCD_gp100_ > 100 (Fig. 3e). Slingshot and ELPIGraph-based pseudotime analysis^38,39^, two methods specifically validated for datasets of dozens to few hundreds of cells or samples, consistently identified trajectories initiating from early transitory cells entering the LN. These cells progress through an intermediate NC-like phenotypic stage, from which colony-expanding DCCs further transition to late transitory, undifferentiated or melanocytic phenotypes (Fig. 3f,g and Extended Data Fig. 5a–c).

### NC-like DCCs display immune-regulated pathways

**Fig. 4:**
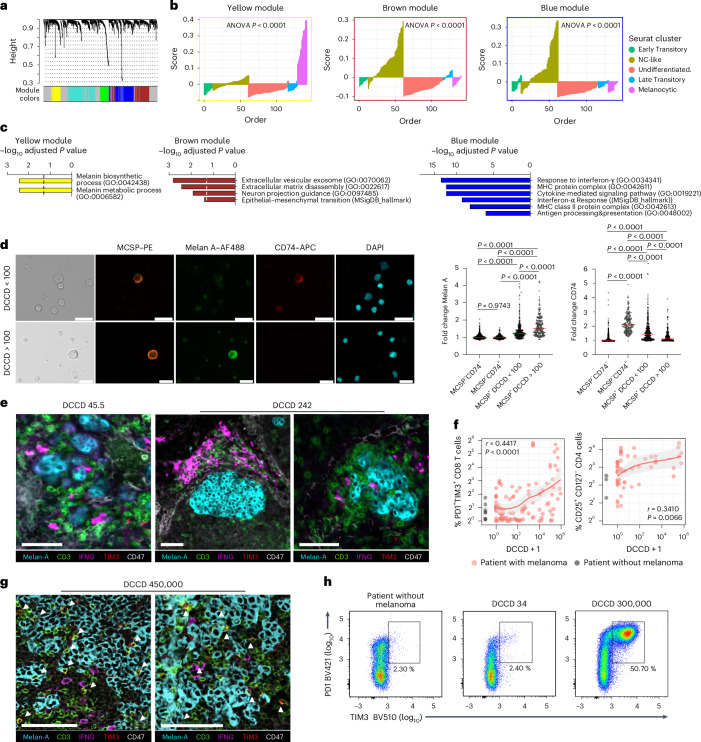
NC-like DCCs crosstalk with immune cells. **a**,**b**, scWGCNA using top variable genes. Cluster dendrograms group genes into distinct modules (**a**), with module score summaries plotted against DCC samples of each Seurat cluster (*x* axis) (**b**). **c**, Enrichr gene set enrichment analysis. Enriched Gene Ontology and Hallmark collection terms in the Molecular Signature Database assigned to genes in brown, blue and yellow modules. **d**, Immunofluorescence staining and quantification of CD74 and Melan A in MCSP^+^ and MCSP^−^ cells in SLNs with DCCD < 100 (*n* = 28 patients per SLN) and DCCD > 100 (*n* = 13 patients per SLN) and cell nuclei (DAPI). Plots depict the fold change in gray of Melan A or CD74 immunofluorescence of MCSP^+^ (DCCD < 100, *n* = 402 cells; DCCD > 100, *n* = 230 cells) and MCSP^−^CD74^+^ cells (*n* = 366 cells) relative to MCSP^−^CD74^−^ cells (*n* = 765 cells). **e**, CODEX multiplex immunofluorescence for Melan A, CD3, IFNG, TIM3 and CD47 in SLN (*n* = 2 patients) with incipient metastatic colonization. Note the absence of CD3 and TIM3 double-positive cells. Scale bars, 50 µm. **f**, Percentage of PD1^+^TIM3^+^ CD8 T cells and CD4^+^CD25^+^CD127^−^ T_reg_ cells in LNs from patients with melanoma (red; *n* = 116 for PD1^+^TIM3^+^ CD8 T cells and *n* = 57 for T_reg_ cells) and patients without melanoma (gray; *n* = 8 for PD1^+^TIM3^+^ CD8 T cells and *n* = 3 for T_reg_ cells) as a function of their DCCD values on a log scale. The red line provides the percentages for the model where log_10_(DCCD + 1) is entered as a continuous variable (gray area, 95% CI). **g**, CODEX multiplex immunofluorescence imaging for Melan A, CD3, IFNG, TIM3 and CD47 (as ubiquitously expressed cell surface protein) in an LN with high DCCD. White triangles indicate CD3 and TIM3 double-positive cells. Scale bars, 100 µm. **h**, Representative flow cytometric analysis of PD1 and TIM3 expression in CD3^+^CD8^+^ T cells from LNs of a patient without tumor and two LNs from a patient with melanoma from the same regional bed. *P* values in **b** were determined using a one-way ANOVA. *P* values in **c** were determined using a hypergeometric test. *P* values in **d** were determined using a one-way ANOVA with Dunn’s post hoc analysis. *P* values in **f** were determined using Pearson’s correlation. All statistical tests were two-sided. Source data

**Extended Data Fig. 6:**
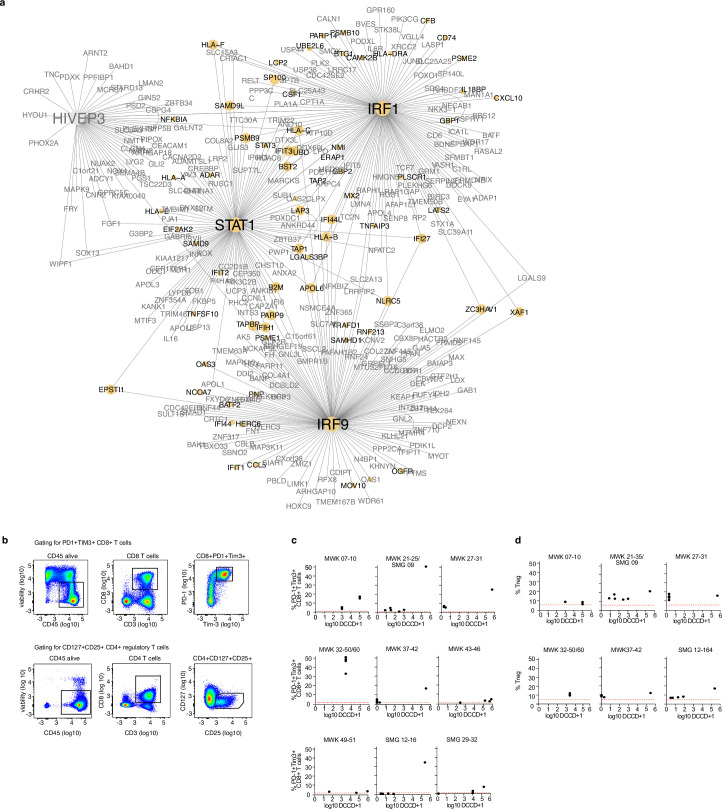
The phenotypes of DCC and immune cells depend on colonization stage and their interaction. **a**, Transcription factor regulatory network highly activated in cluster 1 DCC. Black letters and yellow circles indicate genes involved in interferon alpha/gamma signaling pathways. **b - d**, Flow cytometric analysis of sentinel and regional LNs from melanoma patients for PD1^+^TIM3^+^ CD8 T and CD4^+^CD25^+^CD127^-^ regulatory T cells. Gating as indicated (**b**). Percentage of PD1^+^TIM3^+^ CD8 T cells (c; n = 9 patients) and CD4^+^CD25^+^CD127^-^ regulatory T cells (d; n = 6 patients) plotted against the DCCD of LNs. Each dot represents one LN originating from the same regional bed of the corresponding melanoma patient. Patient IDs are indicated above each graph. Red dotted lines indicate the median percentage of PD1^+^TIM3^+^ CD8 T (n = 16 LNs) or CD4^+^CD25^+^CD127^-^ T cells (n = 17 LNs) in DCC-free LNs of melanoma patients. Source data

To identify pathways characteristic of different phenotypes, we performed single-cell-weighted gene coexpression network analysis (scWGCNA; Fig. 4a), revealing gene modules that were significantly differentially activated between Seurat clusters, with significantly enriched pathways (all *P* < 0.0001, ANOVA; Fig. 4b). For example, melanocytic DCCs (Seurat group 4) were enriched for melanin biosynthetic and metabolic processes (yellow module; Fig. 4b,c). In contrast, NC-like DCCs (Seurat group 1) were enriched for the GO terms extracellular vesicular exosome and epithelial–mesenchymal transition (brown module) or interferon-γ (IFNG) response and antigen processing and presentation (blue module) (Fig. 4c), supported by gene-regulatory network analysis (Extended Data Fig. 6a). Pathway analysis based on differentially expressed genes (DEGs) showed highly consistent results with scWGCNA (Supplementary Tables 1–5). Notably, activation of the blue module in NC-like cells resembled a recent analysis of LN metastasis in a mouse model^34^, where B16 melanoma cells selected for LN metastasis upregulated immune pathways similar to those seen in human NC-like DCCs during early metastatic colonization, switching from early transitory (cluster 0) to NC-like (cluster 1) gene expression (Reticker-Flynn mouse model; Extended Data Fig. 3i).

To validate the NC-like intermediate phenotype before colony expansion, we stained an independent set of patient samples (*n* = 41 SLNs, 41 patients) for MCSP (DCC marker), Melan A (melanocytic marker) and CD74, the invariant light chain of the human leukocyte antigen (HLA) class II histocompatibility complex. CD74 was selected, as it is a marker gene (Supplementary Table 6) for NC-like Seurat cluster 1 and part of the antigen-presenting pathway upregulated by IFNG. Consistent with pseudotime analysis, CD74 expression was significantly higher in MCSP^+^ cells in the DCCD < 100 (NC-like) group than in the DCCD > 100 group, which included further differentiated cells with higher Melan A expression (Fig. 4d).

The activation of the IFNG pathway in NC-like DCCs suggested interactions with T cells early after arrival in the lymph node. This was confirmed by multiplexed immunofluorescence imaging using codetection by indexing (CODEX), which identified IFNG-producing T cells in the direct cellular neighborhood to small DCC clusters and micrometastases (Fig. 4e). Because extracellular vesicles (EVs) mediate cell-to-cell communication and are upregulated in NC-like DCCs (brown module, Fig. 4c), we searched for candidate recipient cells of EV-transmitted signals. Flow cytometry of SLNs and regional LNs of patients with melanoma revealed a rise in exhausted programmed cell death protein 1 (PD1)^+^ and T cell immunoglobulin and mucin domain-containing protein 3 (TIM3)^+^ CD8 T cells and CD127^−^CD25^+^ regulatory T (T_reg_) cells with increasing DCCD_gp100_ (*P* < 0.0001 for CD8 T cells and *P* = 0.0066 for T_reg_ cells, Pearson’s correlation; Fig. 4f). CODEX imaging corroborated this, showing a higher frequency of TIM3^+^ T cells in LNs with high DCCD compared to those with incipient metastatic colonization (Fig. 4e,g). Analysis of patients with LNs from the same regional bed and varying DCCD_gp100_ levels (Fig. 4h and Extended Data Fig. 6b–d) indicated that the rise in exhausted CD8 T cells was locally associated with metastatic colonization and not a systemic effect. This prompted further investigations into whether DCC-derived EVs affect CD8 T cell function during metastatic LN colonization.

### IFNG-induced plasticity drives T cell suppression by sEV

**Fig. 5:**
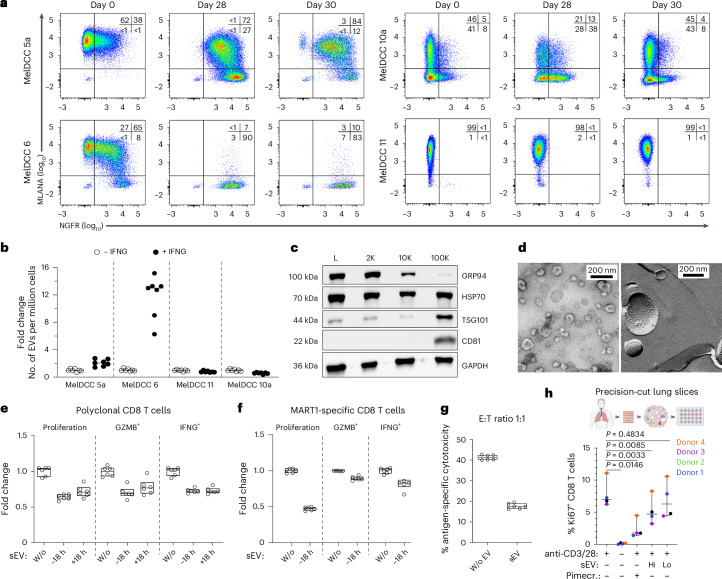
IFNG-induced acquisition of the NC-like phenotype enhances secretion of T cell-suppressive sEVs by melanoma DCCs. **a**, Flow cytometric analysis of MelDCC 5a, 6, 10a and 11 for Melan A and NGFR expression before (day 0) and after 4 weeks of IFNG treatment (day 28) or after 4 weeks of IFNG treatment followed by 48 h in IFNG-free EV production medium (day 30). **b**, Fold change in the number of sEVs secreted per million MelDCCs over 48 h, either untreated or treated with IFNG for 28 days, followed by 48 h in IFNG-free EV production medium (*n* = 6 technical replicates each). **c**, WB analysis of EV pellets (2K, 10K and 100K) isolated from MelDCC 10a, using antibodies for small (CD81 and TSG101) and large (GRP94) EV markers or the pan-EV marker HSP70. L, whole-cell lysate. **d**, TEM of 100K preparations. Left, negative staining; right, freeze-etching. **e**,**f**, Flow cytometric analysis of proliferation and effector cytokine production (IFNG and GZMB) in polyclonal (**e**) or MART1_27L26-35_-specific (**f**) CD8 T cells exposed to sEV from MelDCC 10a or PBS for 18 h before (−18 h) or after anti-CD3/CD28 stimulation (+18 h) (*n* = 6 technical replicates each). **g**, Cytotoxic activity of MART1_27L26-35_-specific CD8 T cells exposed or not to sEVs from MelDCC 10a 18 h before anti-CD3/CD28 stimulation. CD8 T cells were harvested on day 4 and added at an effector-to-target ratio of 1:1 to MART1_27L26-35_-loaded CFSE-labeled T2 cells. Nonloaded, CellTrace violet-labeled T2 cells served as nontarget controls (*n* = 6 technical replicates each). **h**, Flow cytometric analysis of Ki67^+^ CD8 T cells in human PCLSs (*n* = 4 patients) 5 days after anti-CD3/CD28 stimulation and exposure to high or low doses of sEVs (sEVs produced by 6.25 × 10^6^ or 1.25 × 10^6^ MelDCC 10a cells) or 5 µM pimecrolimus. Nonstimulated PCLSs served as the control. sEVs or pimecrolimus was added 18 h after stimulation. Shown is the median (line) with range (whiskers) delineating the minimum and maximum values. *P* values in **h** were determined using a one-way ANOVA with Dunnett’s post hoc multiple-comparison test (two-sided). Boxes represent the median, lower quartile and upper quartile, with individual data points illustrating the data distribution. Source data

**Extended Data Fig. 7:**
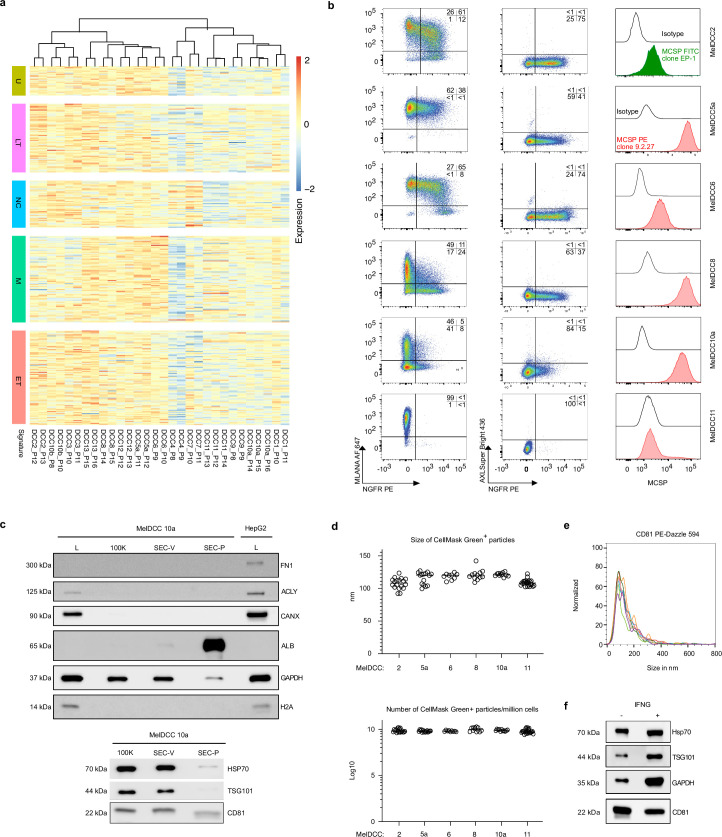
DCC-derived cell lines reflect the diversity of ex vivo DCC phenotypes. **a**, Expression of DCC-derived marker genes for the different phenotypes of early and late transitory (ET, LT), neural crest-like (NC), undifferentiated (U), and melanocytic (M) phenotypes in MelDCC 1-13, analyzed by bulk RNAseq in duplicates or triplicates of consecutive passages. **b**, Flow cytometric analysis of MelDCC 2, 5a, 6, 8, 10a, and 11 for NGFR (neural crest), Melan A (melanocytic), AXL (invasiveness), and MCSP expression. MelDCC2 was stained with anti-MCSP-FITC (clone EP-1), all other MelDCC lines with anti-MCSP-PE (clone 9.2.27). **c**, Western blot of 100 K sEVs or size-exclusion chromatography-separated 100 K sEV vesicle (SEC-V) and protein (SEC-P) fractions. Markers for exomeres (ACLY), and non-EV contaminants (fibronectin, FN1; calnexin, CANX; histone H2A; albumin, ALB) and sEV (CD81, TSG101), and pan-EV (HSP70, GAPDH) were used. Loaded per lane: 2.5×10^6^ sEVs, 10^6^ cells, 10 µg whole cell lysate (L). The albumin signal in SEC-V and SEC-P samples is due to the absence of a washing step in the SEC workflow, unlike ultracentrifugation. **d**, Particle size and NTA quantification of sEVs secreted per million MelDCC cells over 48 h. MelDCC 2, 5a, 6, 8, 10a, and 11 (n = 18, 15, 9, 12, 11, and 23). N = biological replicates. **e**, Size distribution of CD81^+^ 100 K sEVs from MelDCC 10a (n = 7 biological replicates, indicated by different color codes) determined by NTA. **f**, Western blot of 100 K sEVs from untreated or IFNG-treated MelDCC 6 for 4 weeks. Loaded are sEVs from 0.5×10^6^ cells/lane. Source data

We used previously established melanoma DCC-derived (MelDCC) cell lines from LN-derived DCCs^2^, encompassing the different DCC phenotypes (Extended Data Fig. 7a,b), to test whether NC-like cells secreted more small EVs (sEVs, previously called exosomes), as suggested by our pathway analysis. After 28 days of IFNG exposure, the phenotypically plastic MelDCC 5a and 6 lines differentiated into NC-like cells expressing nerve growth factor receptor (NGFR) (Fig. 5a). To distinguish sEV from nonvesicular particles, we used the lipid membrane dye CellMaskGreen (CMG) for nanoparticle tracking analysis (NTA). Because of the interference of IFNG and BSA with CMG-based NTA, sEV secretion into the culture supernatant was measured 48 h after IFNG and BSA withdrawal (days 28–30). Controls included MelDCC 10a and 11, which did not acquire (MelDCC 11) or quickly lost (MelDCC 10a) the NC-like phenotype during the IFNG-free EV production phase (Fig. 5a). NTA showed increased sEV production in NC-enriched, IFNG-treated MelDCC 5a and 6 cultures (Fig. 5b).

To confirm NTA results by western blot (WB) and obtain purified sEV preparations for testing the effect of sEVs on CD8 T cell function, we isolated sEVs through differential ultracentrifugation (dUC)^40^ from culture supernatants of MelDCC lines. The purity of the 100,000*g* (100K) pellet was confirmed by strong signals for the sEV markers TSG101 and CD81 and the absence of GRP94, a marker for larger EVs (Fig. 5c). Further separation of the 100K pellet into vesicular and protein fractions by size-exclusion chromatography (SEC) confirmed the absence of nonvesicular proteins and exomeres and the high purity of sEV in the 100K pellet (Extended Data Fig. 7c). Transmission electron microscopy (TEM) and NTA further revealed intact sEVs with a median size of 108–120 nm, production ranging from 5.2 × 10^9^ to 9.5 × 10^9^ sEVs per million cells within 48 h and the presence of proteins in their membrane (Fig. 5d and Extended Data Fig. 7d,e). The WB analysis of sEVs from IFNG-treated MelDCC 6 (Extended Data Fig. 7f) corroborated the NTA findings (Fig. 5b), linking the IFNG-induced NC phenotype with increased EV production.

**Extended Data Fig. 8:**
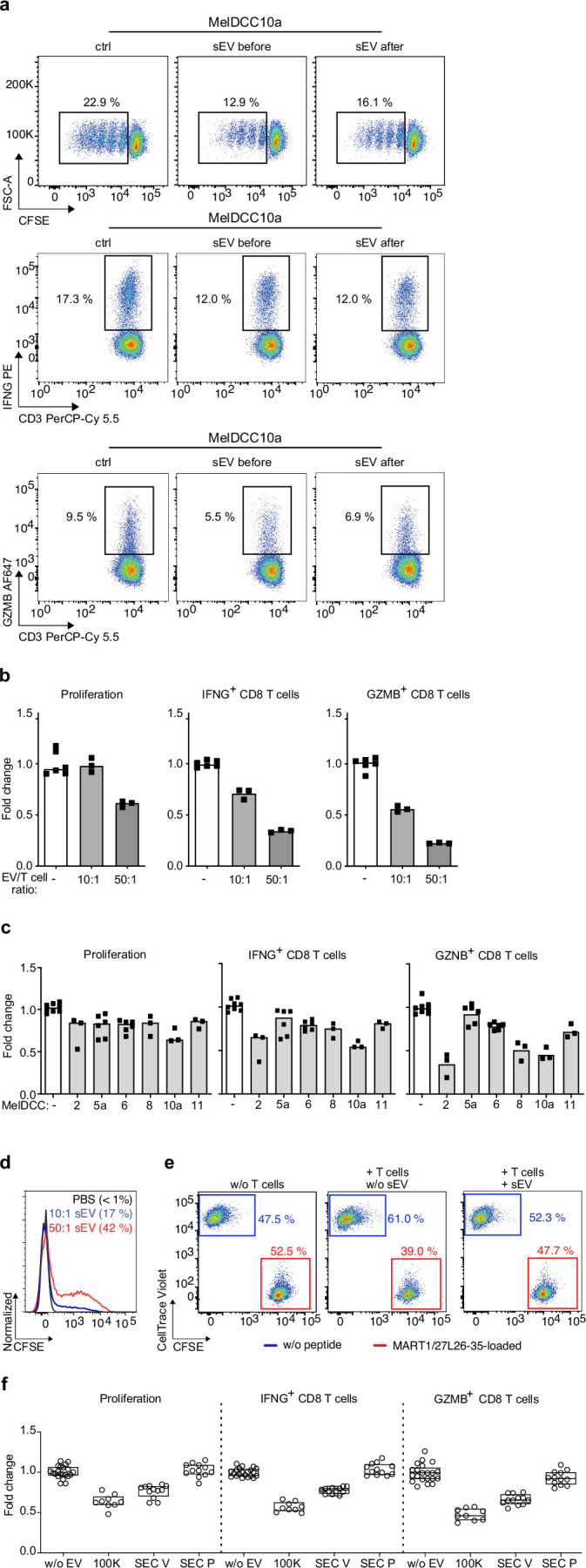
MelDCC-derived sEVs suppress CD8 T cell proliferation and function. **a-c**, CD8 T cell proliferation and IFNG/GZMB production 4 days after anti-CD3/CD28 stimulation. Cells exposed to PBS or sEVs (MelDCC 10a in panel a-c; MelDCC 2, 5a, 6, 8, 10a, 11 in panel c). sEVs were added 18 h before stimulation at a 50:1 ratio unless otherwise noted. Experiments were performed in two independent experimental sets with n = 3 (w/o EV, MelDCC2, 8, 10a, 11), n = 6 (MelDCC5a, 6) technical replicates per MelDCC, and results were normalized to the respective PBS control (n = 3 technical replicates for MelDCC 2, 8, 10a, 11 and n = 5 for MelDCC 5a, 6) to enable a pooled comparative analysis. Bars indicate the median, with individual data points illustrating the data distribution (b,c). **d**, Uptake of CFSE-labeled sEVs by anti-CD3/CD28-stimulated polyclonal human CD8 T cells incubated with PBS or sEVs for 24 h at a 50:1 or 10:1 ratio (n = 3 technical replicates). **e**, Flow cytometric analysis of cytotoxicity using MART_127L26-35_-loaded, CFSE-labeled T2 target cells (red gate) and non-loaded, CellTrace Violet-labeled T2 cells (blue gate) as non-target controls. In the presence of antigen-specific T cells, the fraction of target cells (red gate) decreases due to the cytotoxic T cell activity as compared to non-target control cells (blue gate). The cytotoxicity of T cells is decreased when CD8 T cells were exposed to sEV prior to anti-CD3/CD28 stimulation. **f**, CD8 T cell proliferation and IFNG/GZMB production 4 days after exposure to PBS, 100 K sEVs or SEC-V/SEC-P-fractions derived from 100 K sEVs of MelDCC 10a. Experiments with SEC-V/P and 100 K sEVs were performed independently with n = 12 technical replicates (SEC-V/P) and n = 9 (100 K, 3 experiments with 3 technical replicates each), and results were normalized to the respective PBS controls to enable a pooled comparative analysis. Boxes represent the median, lower quartile, and upper quartile, with individual data points illustrating the data distribution. Source data

To assess the effect of sEVs on CD8 T cells, we added sEVs (100K) of six MelDCC lines to healthy donor-derived polyclonal (Fig. 5e and Extended Data Fig. 8a–c) or MART1_27L26-35_-specific CD8 T cells (Fig. 5f). sEVs were added 18 h before or after anti-CD3 and anti-CD28 (anti-CD3/CD28) stimulation in a concentration of 10:1 or 50:1 (that is, sEVs produced by 10 and 50 MelDCC cells per CD8 T cell, respectively). Dose-dependent uptake of sEVs was confirmed (Extended Data Fig. 8d). Coculture with polyclonal or antigen-specific CD8 T cells showed significant suppression of proliferation and differentiation into cells producing IFNG and granzyme B (GZMB), irrespective of whether sEVs were added before or after stimulation (Fig. 5e,f and Extended Data Fig. 8a). Notably, effector cytokine production was suppressed at lower sEV concentrations compared to proliferation (Extended Data Fig. 8b). Moreover, sEVs reduced the antigen-specific ability of CD8 T cells to eliminate target cells (Fig. 5g and Extended Data Fig. 8e). SEC removal of proteins from the 100K pellet confirmed that sEVs but not copurified proteins or exomeres were responsible for the suppressive effect (Extended Data Fig. 8f). Lastly, in ex vivo-derived human precision-cut lung slices (PCLSs) from four patients, sEVs significantly reduced the Ki67^+^ CD8 T cell frequency dose dependently (Fig. 5h).

### DCCs and their sEVs carry CD155 and CD276 but not PD1 ligand 1

**Fig. 6:**
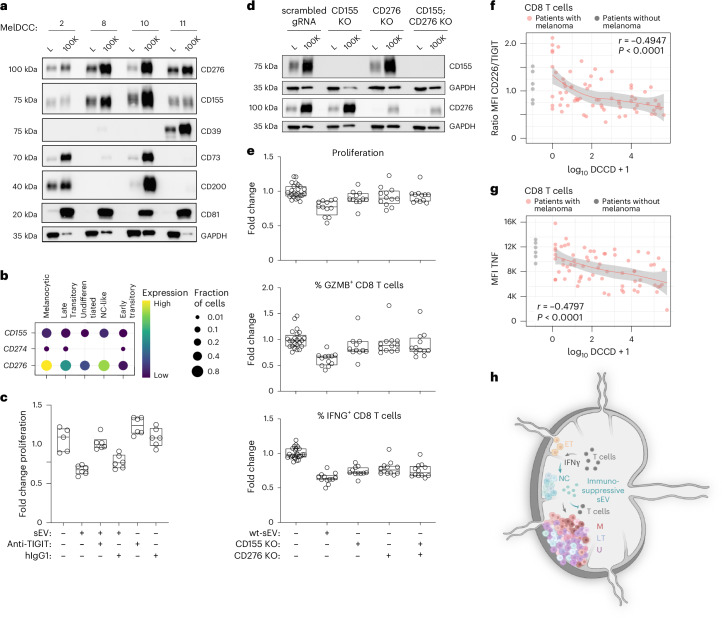
Melanoma DCCs suppress T cells through CD155 and CD276. **a**, WB for various ICLs in MelDCC lines and their respective 100K pellets. **b**, Expression of ICLs in DCCs separated according to their phenotype (scRNA-seq data of DCCs; *n* = 164 cells). **c**, Flow cytometric analysis of CD8 T cell proliferation on day 4 after anti-CD3/CD28 stimulation and addition of PBS (*n* = 5 technical replicates) or MelDCC 10a sEVs in the presence or absence of 10 µg of anti-human TIGIT antibody or human IgG1 isotype control (all *n* = 6 technical replicates). The bar indicates the median. **d**, WB for CD155 and CD276 expression in MelDCC 10a controls or CD155;CD276 single or double knockout and their respective 100K pellets. KO, knockout; gRNA, guide RNA. **e**, Flow cytometric analysis of CD8 T cell proliferation and percentage of IFNG^+^ and GZMB^+^ CD8 T cells on day 4 after anti-CD3/CD28 stimulation and addition of PBS or sEVs of MelDCC 10a (wild-type sEVs) and MelDCC 10a with CD155;CD276 single or double knockout 18 h before anti-CD3/CD28 stimulation. Experiments with CD155 or CD276 single-knockout or CD155;CD276 double-knockout sEVs were performed independently with *n* = 11–12 technical replicates per condition and results were normalized to their respective PBS controls to enable a pooled comparative analysis. **f**,**g**, Flow cytometric analysis of CD226:TIGIT (**f**) and TNF (**g**) expression in CD8 T cells from LNs of patients with melanoma (*n* = 69 LNs) and patients without melanoma (*n* = 6 LNs). Mean fluorescence intensity of marker expression by CD8 T cells as a function of patient DCCD. The red line provides the LOESS regression where log_10_(DCCD + 1) is entered as a continuous variable (gray area, 95% CI; values for patients with and without melanoma are presented as red and gray dots). **h**, Synopsis of DCC phenotype switching during metastatic colony formation in human melanoma. Acquisition of the NC-like phenotype enables DCCs to suppress early CD8 T cell attack by immunosuppressive sEVs. M, melanocytic; U, undifferentiated; ET and LT, early and late transitory phenotypes. *P* values in **f**,**g** were determined using Pearson’s correlation (two-sided). Boxes represent the median, lower quartile and upper quartile, with individual data points illustrating the data distribution. Source data

**Extended Data Fig. 9:**
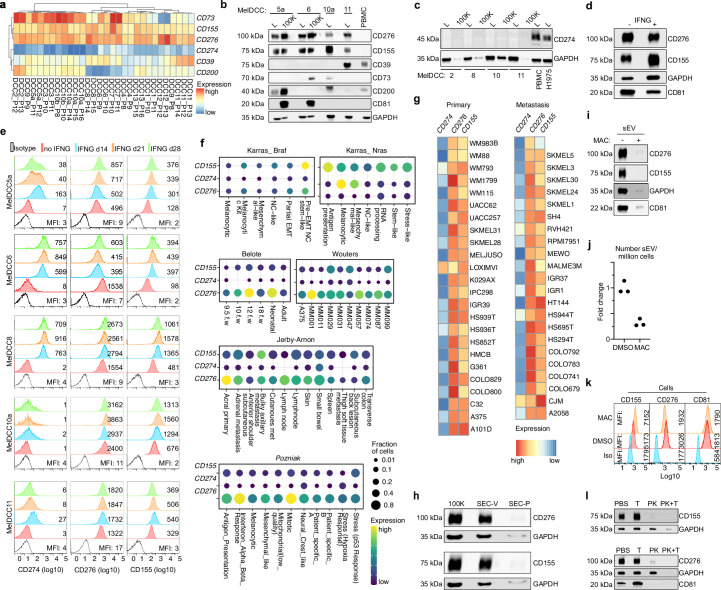
The immune checkpoint ligands CD155 and CD276 are expressed by MelDCC and associated with sEV. **a**, Transcript Expression of *CD155*, *CD274* and *CD276* by MelDCC 1-13. Each cell line was analyzed by bulk RNAseq and in duplicates or triplicates of consecutive passages. **b, c**, Western blot analysis for presence of ICL in whole cell lysates of MelDCC lines (L) and their respective 100 K pellets. sEVs from 2.5×10^6^ cells/lane and 15 µg whole cell lysates/lane were loaded. **d**, Western blot analysis for presence of the ICLs CD155, CD276 and the EV markers CD81 and GAPDH in 100 K sEVs of MelDCC 6 untreated or treated with IFNG for 4 weeks. 2×10^9^ sEVs/lane were loaded, as determined by NTA. **e**, Flow cytometric analysis of CD155, CD274 and CD276 expression in MelDCC lines cultured in the absence (red) or presence of 500 U IFNG for 14 (blue), 21 (orange) or 28 (green) days. As isotype controls (black line) did not differ between the time points, only the isotype control of untreated cells at d0 is shown. **f, g**, Expression of *CD155*, *CD276* and *CD274* in publicly available scRNA-seq data of melanoma ^31–33,35,36^ (**f**) or bulkRNAseq of cell lines of the Cancer Cell Line Encyclopedia^37^ (**g**). **h**, Western blot analysis for the presence of CD155 and CD276 in a 100 K EV-preparation separated by size exclusion chromatography into a vesicle (SEC-V) and protein (SEV-P) fraction. sEVs from 2.5×10^6^ cells/lane were loaded. **i-k**, Inhibition of EV-biogenesis in MelDCC 6 by 50 µM Macitentan. Western blot for the ICLs CD155 and CD276 and the EV-markers CD81 and GAPDH in 100 K sEVs isolated from untreated or Macitentan-treated MelDCC 6. sEVs from 0.5×10^6^ cells/lane were loaded (**i**). NTA-based enumeration of sEVs secreted per million cells within 48 h of DMSO or Macitentan-treated MelDCC 6 (j, n = 3 technical replicates). Flow cytometric analysis of cell surface expression of CD155, CD276 and CD81 on MelDCC 6 treated with DMSO or Macitentan (**k**). **l**, Western blot analysis of sEVs treated with PBS, Triton X-100, Proteinase K or Proteinase K plus Triton X-100 for CD81, CD155, CD276 and GADPH. sEVs from 0.5×10^6^ cells/lane were loaded. Source data

The inhibition of CD8 T cell proliferation and cytokine production by sEVs suggested the presence of inhibitory immune checkpoint ligands (ICLs) on sEVs, as reported for CD274 (PD1 ligand 1 (PDL1))^41^. Transcriptomics (Extended Data Fig. 9a) and mass spectrometry (MS) proteomics data (Supplementary Table 7) from MelDCC lines and sEVs were analyzed and candidate ICLs were confirmed through WB (Fig. 6a and Extended Data Fig. 9b,c). CD155 and CD276 emerged as key candidates because of their constitutive expression in all MelDCC lines (Fig. 6a and Extended Data Fig. 9a,b), with slight IFNG-induced changes in both cells and sEV (Extended Data Fig. 9d,e). In contrast, CD39, CD73 and CD200 showed cell-line-specific expression, while CD274 expression depended on exogenous IFNG (Extended Data Fig. 9e). Notably, *CD155* and *CD276* were significantly more frequently and more highly expressed than *CD274* in SLN DCCs (*P* < 0.0001, Fisher’s exact test; Fig. 6b), primary tumors, metastases^31,36^, melanoma cell lines^32,37^, human epidermal melanocytes^35^ and mouse melanoma models^33^ (Extended Data Fig. 9f,g). CD155 and CD276 were confirmed to be associated with sEVs using SEC and inhibition of EV secretion by macitentan^42^ (Extended Data Fig. 9h–k). Proteinase K (PK) treatment of sEVs confirmed their surface localization, with PK degrading CD155 and CD276 but not the luminal protein glyceraldehyde 3-phosphate dehydrogenase (GAPDH)^40^ (Extended Data Fig. 9l).

### sEV-associated CD155 and CD276 mediate CD8 T cell suppression

**Extended Data Fig. 10:**
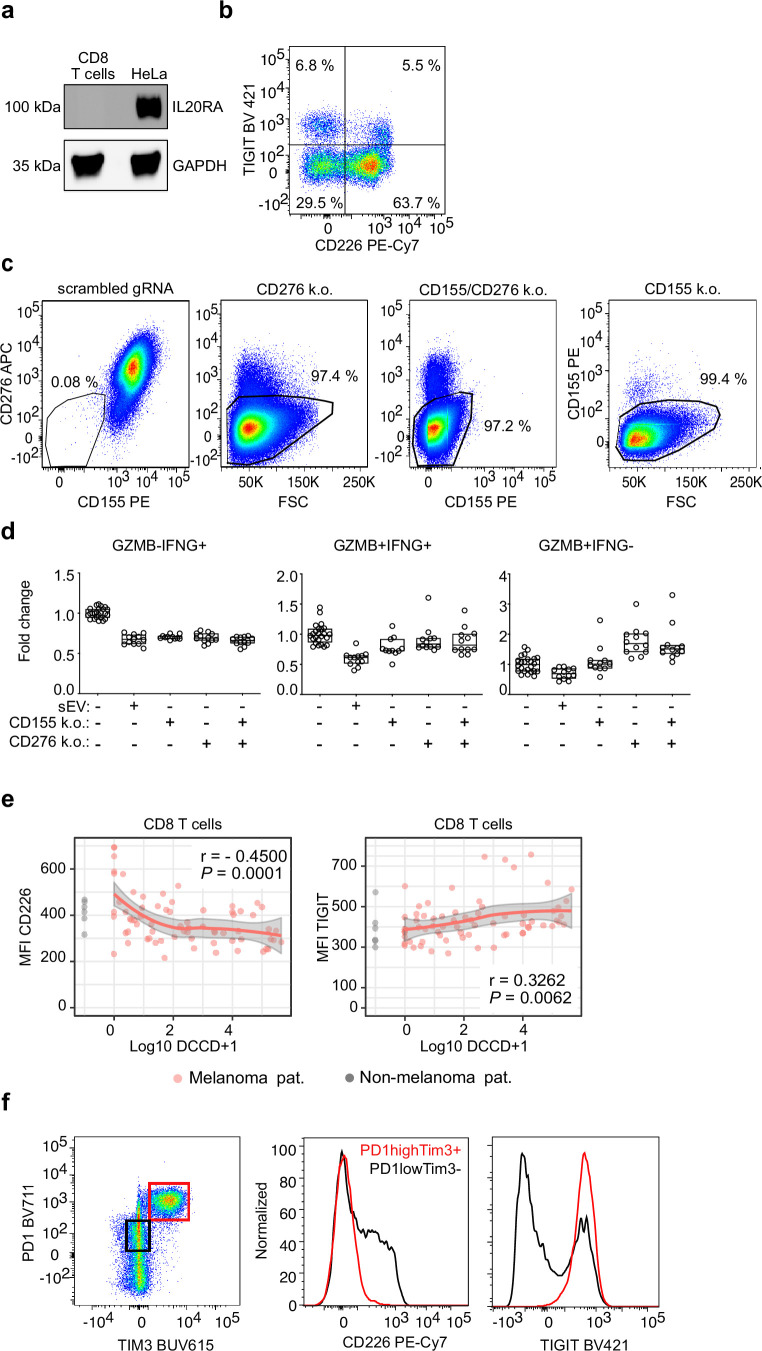
sEV-associated CD155 and CD276 suppress CD8 + T Cell proliferation and function. **a**, Western blot analysis for IL20RA expression in CD8 T cells from a healthy donor and HeLa cells as positive control. 20 µg of whole cell lysates/lane were loaded. **b**, Flow cytometric analysis of TIGIT and CD226 expression in CD8 T cells from peripheral blood of a healthy donor. **c**, Flow cytometric analysis for CD155 and CD276 expression in MelDCC 10a CD155;CD276 single or double knock-outs and scrambled gRNA electroporated control. **d**, Flow cytometric analysis of CD8 T cell proliferation and percentage of GZMB^-^IFNG^+^, GZMB^+^IFNG^+^ and GZMB^+^IFNG^-^ CD8 T cells at day 4 after anti-CD3/CD28 stimulation and addition of PBS or sEVs of MelDCC 10a (wt EV) and MelDCC 10a with CD155/CD276 single or double knock-out 18 h before anti-CD3/CD28 stimulation. Experiments with CD155 single and CD276 single/double-knock out sEV were performed independently with n = 11 (CD155 k.o.) and n = 12 (CD276 k.o., CD155/CD276 k.o.) technical replicates per group, and results were normalized to the respective PBS controls to enable a pooled comparative analysis. **e**, Flow cytometric analysis of TIGIT and CD226 expression in polyclonal CD8 T cells from LNs of melanoma patients (n = 69 LNs) and nonmelanoma patients (n = 6 LNs). MFI of marker expression by CD8 T cells as a function of patient DCCD. The red line provides LOESS regression where log10(DCCD + 1) is entered as a continuous variable (grey area, 95% CI; values for melanoma and nonmelanoma patients presented as red and grey dots, respectively). **f**, Expression levels of TIGIT and CD226 in PD1^high^TIM3^high^ (red gate in dot plot and red line in histograms) and PD1^low^TIM3^low^ (black gate in dot plot and black line in histograms) of a patient LN from panel e) with high DCCD (250.000). *P* values in **e**, according to Pearson’s correlation, two-sided. Boxes represent the median, lower quartile, and upper quartile, with individual data points illustrating the data distribution. Source data

We next analyzed the expression of CD155 and CD276 receptors on healthy donor-derived CD8 T cells. The putative CD276 receptor, the α-subunit of the CD276 interleukin-20 receptor^43^, was not detected (Extended Data Fig. 10a), ruling out its role in CD276-mediated suppression of CD8 T cells by sEVs. In contrast, 12% of CD8 T cells expressed the inhibitory T cell immunoreceptor with Ig and ITIM domains (TIGIT) and 69% expressed the stimulatory receptor CD226, both of which bind CD155 (Extended Data Fig. 10b). Blocking the TIGIT–CD155 interaction with an anti-TIGIT antibody neutralized the immunosuppressive effect of sEVs (Fig. 6c). Moreover, knockout of CD155 or CD276 (Fig. 6d and Extended Data Fig. 10c) reduced the suppressive effect of sEVs on T cell proliferation, with no additional reduction from the double knockout (Fig. 6e). Knockouts increased the percentage of GZMB^+^ cells but minimally affected the percentage of IFNG^+^ cells, suggesting other suppressive molecules or varying ICL susceptibilities among IFNG and GZMB CD8 T cell subpopulations (Extended Data Fig. 10d).

TIGIT competes with CD226 for CD155 binding but binds CD155 with higher affinity^44^. As binding of tumor-associated CD155 to CD226 triggers its ubiquitination and proteasomal degradation^45^, a decreasing ratio of CD226:TIGIT indicates previous CD155 binding. Testing 75 SLNs and regional LNs from patients with melanoma revealed a negative correlation between the CD226:TIGIT ratio on CD8 T cells and the DCCD (*P* < 0.0001, Pearson’s correlation; Fig. 6f). This ratio decline, primarily driven by reduced CD226 expression (Extended Data Fig. 10e), was most pronounced at the precolonizing stage (DCCD < 100; Fig. 6f and Extended Data Fig. 10e) and culminated at high DCCD in PD1^+^Tim3^+^ CD8 T cells completely lacking CD226 (Extended Data Fig. 10f). The CD226:TIGIT ratio decline paralleled reduced tumor necrosis factor (TNF) production by CD8 T cells (*P* < 0.0001, Pearson’s correlation; Fig. 6g). These findings, together with the IFNG-induced increase in sEV secretion by NC-like cells, highlight that immune selection shapes DCC adaptation in patients with melanoma already at the precolonizing stage and links the NC phenotype to immune evasion that extends beyond the mere loss of melanoma differentiation antigens.

## Discussion

In this study, we dissected the earliest steps of metastatic colony formation in melanoma by analyzing treatment-naive melanoma DCCs in SLNs of patients with melanoma to identify and characterize MFCs. We found that melanoma cells entering the LN predominantly exhibited an early transitory phenotype^29^ and transition to an NC-like phenotype upon IFNG exposure at incipient colony formation. This aligns with recent mouse model data, where melanoma cells transition from a mesenchymal or early transitory phenotype to acquire an IFN signature as they invade from the subcapsular sinus into the cortical region^33,34^. Our findings indicate that T cell attack drives the NC-like transition, with surviving melanoma cells upregulating immunosuppressive sEV production. sEV-mediated immunosuppression likely reduces IFNG levels, thereby enabling phenotype switching from NC-like to other phenotypes and, consequently, metastatic colony formation (Fig. 6h). While the high plasticity of melanoma cells has been observed in various conditions^46–48^, its dynamic nature during the earliest steps of metastasis formation at the secondary site has not been shown before.

To uncover this, we started a scrupulous search for candidate MFCs. Approximately 20 years ago, we had begun to compare gp100, S100, Melan A and MCSP for their ability to detect early DCCs and linked them to patient survival. We found that gp100 was the most accurate prognostic marker^17,18^, while MCSP was initially limited because of specificity issues. With the addition of MTs (*PMEL*, *MLANA* or *DCT*), defining MCSP^+^MT^+^ cells, it surpassed gp100 in terms of clinical impact. Even in the best prognostic group of gp100^−^ or histopathology-negative patients, detection of MCSP^+^MT^+^ cells marked a high risk of melanoma mortality. MCSP expression peaks during human fetal development at week 18 and is preserved across all subtypes in human and murine melanoma^33,35^. It remains to be determined which of the multiple functions of MCSP identified so far confers MFC potential to DCCs^33,35^.

Tracking MFC progression by metastasis formation through the DCCD^2,17^ revealed a correlation among melanoma phenotypes, immune response and early colonization. In addition to the acquisition of genetic alterations^2^, unlike a static CSC phenotype, MFCs apparently display a dynamic, microenvironmentally responsive behavior.

At the transition from single invading DCCs to micrometastasis, the predominant phenotype showed an NC-like gene expression program, an IFN signature indicating immune cell interactions and activation of the exosomal pathway. Our data show that NC-like DCCs use sEV cargo, particularly CD155 and CD276 but not CD274 (PDL1), to impair CD8 T cells and promote early immune escape. The importance of CD155-mediated suppression in early metastatic colonization is highlighted by the decrease in the CD226:TIGIT ratio on CD8 T cells. This decrease is most pronounced at the precolonizing stage and precedes the accumulation of exhausted CD8 T cells. While consistent with experimental data on the role of early immune escape in systemic cancer^11,49^, further investigation is needed to understand the organ and site dependency and the activation state of DCCs in relation to innate versus adaptive immune escape.

Our findings suggest that metastatic competence is selected at metastatic sites and shaped by tumor-intrinsic and microenvironmental factors. Genomic analyses of gp100^+^ DCCs showed that DCCs acquire mutations as they progress from initial colonization to micrometastases^2^. We demonstrate here that phenotypic plasticity and, specifically, survival under INFG exposure trigger the NC-like program at the beginning of metastatic colonization. Our data and the Reticker-Flynn mouse model suggest that immune microenvironment interactions, particularly IFN signaling, enhance the metastatic competency of LN-colonizing cells. These mechanisms include increased production of immunosuppressive sEVs (this study) and a rise in T_reg_ cells^34^. Intriguingly, both studies concur that lymphatic spread increases the frequency of distant metastasis in patients and mice.

In summary, we identified MCSP^+^ DCCs as the strongest candidate for melanoma MFCs and drivers of early immune evasion, laying a foundation for advancing melanoma immunotherapy. Targeting MCSP could be an optimal strategy for eliminating MFCs. First, MCSP is expressed in all phenotypes forming nascent metastases, likely including MFCs in other organs. Second, MCSP expression is restricted and low in nontumor tissue^50^. Moreover, MCSP-specific immunity, whether spontaneous or induced by immunotherapy, has not shown toxicity in patients with melanoma, healthy individuals or animal models^19^. Third, bypassing immunosuppression with MCSP-directed chimeric antigen receptor T cells may prove particularly promising, as demonstrated in a recent study on minimal systemic cancer^51^. Additionally, our study highlights the importance of CD155 and CD276 as possibly superior or complementary targets to PDL1 and PD1 in adjuvant immunotherapy.

## Methods

### Patient inclusion and ethics statement

The study complied with all relevant ethical regulations regarding the use of human material. Human DCCs were obtained from SLNs or regional LNs of patients with melanoma and control skin-draining LNs were obtained from patients without melanoma (no. 07-079 and 18-948-101, ethics committee of the University of Regensburg). Human peripheral blood mononuclear cells were obtained from a healthy donor (no. 20-1991-101, ethics committee of the University of Regensburg) and human tumor-free lung samples were obtained from patients with lung cancer (no. 2701-2015, ethics committee of Medical School Hannover). Written informed consent was obtained from all participants. Participants provided explicit consent for the inclusion of information listed in Table 2 and Source data. Participants did not receive any compensation for their involvement in this study.

### Cell lines

MelDCC lines were derived from DCC xenografts or directly propagated in vitro as described previously^2^. Patient origin was verified by short tandem repeat (STR) analysis (Cell-ID, Promega), melanoma origin was verified by a surgical pathologist and genotype was verified by CNAs. The NCI-H1975 cell line was obtained from the American Type Culture Collection and the HeLa cell line was a gift from T. Hehlgans (Leibniz Institute for Immunotherapy). Both lines were authenticated using STR profiling. MelDCC, NCI-H1975 and HeLa cells were cultured in RPMI 1640 with 10% FBS, 2 mM l-glutamine and 1% penicillin–streptomycin (P/S) (all Pan-Biotech). Adult human epidermal melanocytes (Lonza) were cultured in MGM-4 with supplements and 1% P/S and used at passage 1 for scRNA-seq. All cells were maintained at 37 °C and 5% CO_2_ in humidified conditions and tested for *Mycoplasma* by PCR.

### LN disaggregation and IC

Quantitative IC with primary antibodies to gp100 and MCSP (clone 9.2.27) was performed on unfixed SLN tissue following SLN biopsy or regional LN removal as described previously^2,17,18^. LNs were defined as gp100^+^ or MCSP^+^ if they contained at least one positive cell. The number of positive cells per million lymphocytes (DCCD) was recorded as DCCD_gp100_ or DCCD_MCSP_. MCSP^+^ cells were isolated using a micromanipulator (Eppendorf PatchMan NP2, Eppendorf) and RNA and genomic DNA were extracted using WTA and WGA, respectively. Images of MCSP^+^ cells were acquired on Axiovert 200M (Zeiss) or IX81 (Olympus) microscopes.

For multiparameter immunofluorescence staining for MCSP, CD74 and Melan A, SLN cell suspensions were stained on adhesion slides. After blocking with 300 µl per spot of human TruStain FcX and 5% BSA in PBS for 30 min at room temperature (RT), slides were sequentially stained with primary and secondary antibodies and reagents in 5% BSA in PBS with 150 µl per spot for 30 min (secondary antibodies and reagents) or 60 min (primary antibodies) at RT. Slides were washed three times for 3 min each with PBS between staining steps. Slides were first stained with an unconjugated anti-human MCSP (clone LHM2) antibody, followed by goat anti-mouse IgG1 AF546. Free binding sites of the goat anti-mouse IgG1 antibody were blocked with 5% mouse serum in PBS, followed by staining with anti-human Melan A biotinylated antibody and anti-human CD74 APC antibody. In the final steps, slides were stained with streptavidin AF488, followed by DAPI for 10 min at RT, and fixed with 1% formaldehyde in PBS for 5 min at RT. Slides were stored at 4 °C until image analysis. Images were acquired with an IX81 (Olympus) microscope using identical settings for all samples. Fluorescence intensity quantification for Melan A and CD74 was performed using ImageJ (Fiji). All primary and secondary antibodies and staining reagents are listed in Supplementary Table 8.

### Patient survival analysis

Kaplan–Meier survival curves were generated using the survfit function (survival_3.3-1)^52^. A univariable proportional hazard model was applied to patient features (age, gender, MT status, log_10_ DCCD_MCSP_, log_10_ DCCD_gp100_, N status, thickness and ulceration) using coxph (survival_3.3-1). For multivariable proportional hazard models, only features selected according to selectCox function (pec_2022.05.04) were visualized with ggforest (survminer_0.4.9).

### IHC and CODEX

IHC was performed on LNs and tissue microarrays of primary tumors and metastases using anti-HMB45 and anti-MCSP (clone 9.2.27) antibodies. Staining was automated (Ventana Benchmark ULTRA autostainer with OptiView DAB detection kit) or manual (standard protocol with antigen retrieval in Tris–EDTA buffer at 120 °C for 5 min).

For CODEX multiplexed imaging of LNs with low DCCD, 2.5-µm sections of formalin-fixed paraffin-embedded LN blocks with incipient metastatic colonization were prepared, with every second slide stained for Melan A or MCSP (clone 9.2.27) on a Ventana Benchmark ULTRA autostainer using the OptiView DAB detection kit (Roche Diagnostics), according to the manufacturer’s instructions. One of the remaining slides in between (closest to the Melan A IHC staining with the best tumor single cells and clusters) was selected for CODEX staining and performed as described previously^53^. Following deparafinization, hydration and antigen retrieval (Dako Target Retrieval Solution, pH 9, 108 °C, 20 min), autofluorescence was quenched by bleaching (100 ml of 1× PBS + 18 ml of H_2_O_2_ 30% + 3.2 ml of 1 M NaOH) between two light-emitting diode (LED) plates (Aibecy A5 Ultra Bright 25,000-lux LED light box-tracing pads, AliExpress) for 45 min twice as per the manufacturer’s instructions (Akoya Biosciences). After washing with 1× PBS for 5 min and 1× Tris-buffered saline with Tween-20 for 10 min (Cell Marque), nonspecific binding was blocked with 200 µl of CODEX buffer S2 with 0.065 mg ml^−1^ mouse IgG (Biozol), 0.065 mg ml^−1^ rat IgG (Biozol), 0.43 mg ml^−1^ sheared salmon sperm DNA (Invitrogen, Thermo Fisher Scientific) and a mixture of nonfluorescent CODEX oligonucleotides (Biomers) at a final oligonucleotide concentration of 0.5 mM. After 1 h, slides were incubated overnight at 4 °C with DNA-conjugated antibodies in CODEX buffer S2 (anti-human Melan A, anti-human CD3, anti-human IFNG, anti-human TIM3 and anti-human CD47). Slides were washed with CODEX buffer S2 for 2 min, fixed with CODEX buffer S4 containing 1.6% paraformaldehyde for 10 min, treated with ice-cold 100% methanol (Thermo Fisher Scientific) for 5 min and subjected to final fixation with 3 mg ml^−1^ BS3 fixative (Thermo Fisher Scientific) in PBS for 20 min. Slides were stored in CODEX buffer S4 at 4 °C. CODEX imaging was performed on PhenoCycler Fusion 2.0 (low DCCD) or BZ-X810 microscope with PhenoCycler (high DCCD). Overlay images were created after visual assessment of antibody staining using ImageJ (Fiji, version 2.0.0) and QuPath (version 0.5.1)^54^, respectively, for high-DCCD and low-DCCD tissues. Details on all primary and secondary antibodies and staining reagents are given in Supplementary Table 8.

### WGA and analysis of CNAs

Single-cell genomic DNA was collected during WTA by precipitation and amplified using the Ampli1 WGA kit (Menarini Silicon Biosystems) or previously described methods^23,55^. CNA analysis was performed with the Ampli1TM LowPass kit (Menarini Silicon Biosystems) as per the manufacturer’s instructions. Libraries were sequenced on MiSeq (Illumina) or NovaSeq6000 (Illumina). Genomic coordinates were analyzed with the LowPass bioinformatics pipeline (Menarini Silicon Biosystems) or HIENA (Fraunhofer ITEM) and submitted to Progenetix (version 4.0, 2022)^56^ for cumulative frequency plot generation.

### WTA

mRNA isolation from single cells, reverse transcription and global amplification of first-strand complementary DNA were performed as described previously^22,23^. WTA product quality was assessed by a multiplex housekeeping gene expression assay comprising three genes^4^. High quality was assigned to cells with at least one of three transcripts detected.

### Marker expression analysis in single cells

Endpoint PCR for specific transcripts was carried out on all WTA products as previously described^57^. Primers are given in Supplementary Table 9.

### Next-generation sequencing mRNA library preparation and sequencing

Next-generation sequencing (NGS) mRNA libraries were prepared from single MCSP^+^ cells from patients with and without melanoma and human epidermal melanocytes (Lonza) as previously described^4^. Libraries were quantified on the MiSeq System (MiSeq reagent kit v2, 50 cycles, Illumina), pooled and sequenced on an Illumina NovaSeq6000.

RNA from 1–2 million MelDCC cells was extracted using the RNeasy mini kit (Qiagen) and libraries were generated using the TruSeq Stranded mRNA library prep kit (Illumina). Sequencing was performed (SE-82-10) on a NextSeq550 at the NGS Core Unit, Leibniz Institute for Immunotherapy and University Medical Center Regensburg.

### scRNA-seq data analysis

FastQC (version 0.11.5)^58^ was used for sequence quality evaluation. After trimming with BBDuk, STAR (version 2.6.1c)^59^ and RSEM (version 1.3.1)^60^ were used to get expected gene counts on the basis of GRCh38. Cells with fewer than 50,000 counts, >70% mitochondrial gene counts or <1,000 expressed genes were excluded and genes expressed in at least three cells were kept. Seurat (version 4.1.0)^61^ was used for data processing with the NormalizeData and FindVariableFeatures tools for expression normalization and identification of top 2,000 variable genes. Principal component (PC) analysis dimensions were selected on the basis of Elbowplot and JackStrawplot. Testing of 5, 10, 15, 20, 25 and 30 dimensions revealed that 20 is the minimal number of dimensions to separate melanocytes from DCCs. RunUMAP was performed on the top 20 PCs with the parameters umap.method ‘umap-learn’ (n.epochs = 1,000, mindist = 0.1 and n.neighbors = 15). Clusters were computed using FindNeighbors (reduction = ‘umap’, k.param = 10 and dims = 1:2) and FindClusters (resolution = 0.2).

Cluster robustness and consistency was assessed with clusterMany (clusterExperiment, version 2.12.0)^62^ using multiple algorithms (pam, clara, kmeans, spectral, hierarchicalK and tight), ks = 3:10 and PCs 5, 10, 15, 20, 25 and 30, yielding 288 clustering schemes. Consensus of pairwise cell clusters under 288 clustering schemes was calculated and compared to clusters given by Seurat graph-based clustering. Bluster (version 1.2.1) was used to confirm cluster modularity through UMAP and PC analysis. FindAllMarkers (test.use = ‘mast’) was used to detect cluster-specific markers. DEGs (upregulated) with *P* < 0.01 were input for Enrichr^63^ on the basis of the Gene Ontology 2015 and MSIGDB_HALLMARK 2020 (ref. ^64^) databases.

Gene expression signature scores for melanoma DCC subtypes were calculated with AUCell^65^ (aucMaxRank = 0.3) and rescaled (R package scales, version 1.2.0). Pseudotime and cell trajectories were inferred with slingshot (version 2.0.0)^39^, using UMAP and Seurat clusters, with the cluster that had the lowest DCCD (cluster 0) serving as the start. Trajectories were verified by constructing an Elastic structure^66^ in ElPiGraph.R (version 1.0.0) and UMAP with parameter NumNodes = 6 and GetSubGraph with parameter structure = ‘end2end’. NumNodes = 6 was the minimal number that could bind all Seurat clusters in one tree. The node with lowest DCCD level was set as the tree root. For each lineage retrieved, pseudotime was calculated. Signature score changes along pseudotime were tested (gam, version 1.20.1) with chi-square *P* values in each lineage for both slingshot and ElPiGraph.

Metacells were created for each cluster using scWGCNA (version 0.0.0.9000)^67^ (construct_metacells, *k* = 5, reduction = ‘pca’) and a weighted gene coexpression network was built with the top 1,000 variable genes using WGCNA (version 1.70-3)^68^ (blockwiseConsensusModules, consensusQuantile = 0.3, power = 10, networkType = signed, mergeCutHeight = 0.2 and minModuleSize = 50). Power = 10 was chosen according to pickSoftThereshold. Respective gene modules were obtained accordingly.

Module preservation was assessed with WGCNA (version 1.70-3; modulePreservation and resampling), which confirmed the high conservation of detected modules. Only modules with an ANOVA *P* value < 0.001 between clusters were processed for pathway enrichment analysis using the same method as for DEG gene set analysis.

CONICsmat (version 0.0.0.1)^69^ inferred CNAs using gene expression at chromosome arm levels, including 200 melanocytes^70^ as controls. Chromosome arms with Bayesian information criterion difference > 70 and adjusted *P* value < 0.01 were kept as CNA candidates and posterior probabilities of CNA were binarized at threshold = 0.9.

SCENIC (version 1.2.4)^65^ inferred transcription factor gene-regulatory networks using GENIE3 and motif database (https://resources.aertslab.org/cistarget/databases/old/homo_sapiens/hg19/refseq_r45/mc9nr/gene_based/hg19-tss-centered-10kb-7species.mc9nr.feather). Regulatons with highest scores > 0.3 in ≥5 cells with scores > 0.1 were kept. Differentially activated transcription factors for each cluster were identified using a pairwise *t*-test (Benjamini–Hochberg-adjusted *P* value < 0.05).

Cells from this project were integrated with LN and skin cell types from the Human Cell Atlas^71^ using SCTransform and IntegrateData from the Seurat package, followed by PC analysis and UMAP (dims = 1:30).

### Bulk RNA-seq data analysis of MelDCC

Gene counts were calculated as for scRNA-seq and normalized using log counts per million. Gene set variation analysis^72^ with the marker gene set for each melanoma DCC cluster (0–4) was used to obtain DCC cluster signature scores for each MelDCC line.

### Isolation of sEV by dUC

A total of 2 × 10^6^ MelDCC cells were seeded in T175 cell culture flasks and cultured for 3 days. On day 3, medium was removed, cells were washed with PBS and 30 ml of FBS-free medium was added. Conditioned medium was collected after 48 h and subjected to dUC as previously described^40^. Briefly, the medium was centrifuged at 300*g* for 10 min at 4 °C to remove dead cells and debris, followed by 2,000*g* for 20 min at 4 °C (2 K pellet) and then 10,000*g* (10K pellet) for 40 min at 4 °C and finally at 100,000*g* (100K pellet) for 90 min at 4 °C. Respective pellets were pooled, washed in 35–50 ml of PBS and recentrifuged at the same speed at which they were initially harvested before resuspending in 1 µl of PBS (1 × 10^6^ EV-producing cells) at the time of supernatant harvesting.

### SEC

The 100K pellets from dUC were pooled before the last washing step and loaded onto an SEC Column (Plastic XXL column with 45 ml of 2% BCL agarose bead standard, 50–150 µm, with plastic XXL column frit, Agarose Bead Technologies). The column was connected to a Zetasizer Nano ZS (Malvern Panalytical) for real-time particle detection, allowing separation of the vesicle and protein fractions. The vesicle fraction was concentrated at 100,000*g* for 90 min at 4 °C. The protein fraction was concentrated using a Macrosep Advance 3K device (Pall) at 3,200*g* (30 min, 4 °C) followed by Vivaspin 500 (molecular weight cutoff: 5,000, PES, Sartorius). Both fractions were characterized by WB and used in CD8 T cell assays.

### Label-free MS-based proteomics

The 100K EVs of MelDCC 10a (duplicates) were resuspended in 50 µl of gel-aided sample preparation (GASP) buffer (50 mM Tris-HCl pH 8.8, 6 M urea, 1.5 M thiourea and 4% SDS) and sonicated for 15 cycles 60/30 (BioRuptor pico, Diagenode). After centrifugation (20,000*g*, 15 min, 4 °C), proteins were quantified using the SERVA purple protein assay (SERVA Electrophoresis). Sample preparation for liquid chromatography–MS followed the GASP protocol^73^ with slight modifications^74^. Then, 2 µg of peptides were spiked with 100 fmol of RePLiCal (Polyquant) and analyzed on an Eksigent ekspert nanoLC 400 system coupled to a TripleTOF 5600+ MS instrument (SCIEX). Samples were loaded onto a YMC-Triart C18 trap column (3-μm particle size, 0.5-cm length; YMC America) at a flow rate of 10 µl min^−1^ for 5 min (isocratic conditions A: 0.1% formic acid and 0.1% acetonitrile). Peptides were then separated on a reverse-phase column (YMC-Triart C18, 1.9-µm particle size, 120 Å, flow rate of 5 µl min^−1^, 40 °C) using a 94-min binary acetonitrile gradient (3–40% B in 87 min, 40–45% B in 7 min). Duplicate samples were run twice.

SWATH acquisition was performed with a 50-ms full MS scan (400–1,000 *m*/*z*) followed by 60 SWATH windows (35 ms each, 230–1,500 *m*/*z*). Libraries were generated from pooled samples measured in data-dependent acquisition mode (DDA, TOP20 method) with a full MS scan for 250 ms and MS/MS scans for 50 ms each. MS/MS spectra from the DDA runs were searched against the respective UniProt database (Swissprot-human 12-2021) using ProteinPilot 5.0 and imported into PeakView 2.1 using the SWATH MicroApp 2.0, allowing six peptides per protein and six transitions per peptide. Raw values were normalized to total intensity.

### Immuno-WB

WB was performed as previously described^4^ with minor modifications. Protein (cell lysates) or sEVs were denatured by incubation for 5 min at 95 °C in the presence of 1× Leammli buffer (Bio-Rad) containing 10% 2-mercaptoethanol (Sigma-Aldrich) and loaded on 10% or 4–20% Mini-PROTEAN TGX Stain-Free protein gels (Bio-Rad). Incubation of blotted PVDF membranes with primary antibodies was performed overnight. Details on all primary antibodies, secondary antibodies and staining reagents are given in Supplementary Table 8. After washing, blots were incubated with horseradish-peroxidase-conjugated anti-mouse, anti-rat or anti-rabbit IgG secondary antibodies for 2 h at RT. Protein bands were visualized using SuperSignal West Pico PLUS chemiluminescent substrate (Thermo Fisher Scientific). Chemiluminescence was recorded by a ChemiDoc MP imaging system (Bio-Rad) and analyzed with Image Lab (version 6.1, Bio-Rad).

### NTA measurement of sEV

For the detection of CD81^+^ sEVs, 1 µl of 100K pellet and 1 µl of anti-human CD81 PE/Dazzle 594 were incubated for 30 min at RT in 10 µl of PBS (Gibco) and then diluted 1:10,000 in PBS to a final volume of 10 ml. For sEV enumeration from MelDCC culture supernatants, cells were incubated in EV production medium (serum-free RPMI without phenol red (Gibco), 2 mM l-glutamine and 1% P/S; all Pan-Biotech). After 48 h, the supernatant was harvested, centrifuged at 300*g* for 10 min and filtered (0.22 µm). sEVs were stained with 1 µl of a 1:10 dilution of CMG plasma membrane stain (Thermo Fisher Scientific) in PBS (100 µl, 1 h, RT) and then diluted 1:25–30 in PBS to 1 ml. For EV analysis, default software settings were used. Each measurement scanned 11 cell positions, capturing 30 frames per position. In scatter mode, sensitivity was set to 80 and trace length was set to 15; for CD81-stained or CMG-stained particles, the 500-nm long-pass fluorescence filter was used with sensitivity set to 96 and trace length set to 7 or 10. Data were analyzed with ZetaView Software (version 8.05.14 SP7) and FlowJo (version 10.8.1).

### TEM imaging of EV

EVs were isolated from MelDCC cells by dUC and diluted 1:10 using HEPES-buffered saline (pH 7.4). Then, 4 µl of this suspension were applied onto hydrophilized, carbon-coated grids (400-mesh, Cu; Plano) for 30 s, blotted with filter paper and stained with a 1% uranyl acetate solution for 30 s, before final blotting and air-drying. For freeze-etching, undiluted EVs were processed with unidirectional platinum and carbon shadowing (45°, 1.5 nm) and carbon coating (90°, 15 nm) as described previously^75^. Samples were screened and imaged with a 200-kV field-emitter TEM (JEM-2100F, JEOL) and a 16-megapixel complementary metal–oxide–semiconductor camera (F416, TVIPS). Microscope and imaging parameters were controlled using SerialEM software (version 3.8)^76^. Images were recorded at magnifications from ×2,000 up to ×40,000, resulting in pixel sizes from 5.5 nm to 0.28 nm.

### Inhibition of EV biogenesis by macitentan

MelDCC 6 were incubated with DMSO or 50 µM macitentan (Selleckchem) in culture medium. After 24 h, the medium was replaced with serum-free medium for sEV production or serum-free medium without phenol red for NTA. The medium was supplemented with DMSO or macitentan and cells and supernatants were harvested after 48 h. Cells were analyzed by flow cytometry for cell surface expression of CD155, CD276 and CD81. sEVs for WB analysis were isolated by UC and the fold change in sEV numbers in the native culture supernatant was analyzed by NTA.

### Topology assessment of EV-associated ICLs

sEVs (0.5 × 10^6^ MelDCC cells) were incubated for 2 h at 37 °C in 50 mM Tris-HCl pH 8.0 with 5 mM CaCl_2_ (Sigma-Aldrich) in the presence or absence of 0.125 mg ml^−1^ PK (Promega or Roche) with or without 1% Triton X-100 (Sigma-Aldrich). Proteinase activity was inhibited by incubation with 1 mM PMSF(Sigma-Aldrich) for 10 min at RT before WB against CD155, CD276, CD81 and GAPDH was performed.

### CRIPSR–Cas9 genetic engineering

Knockout of CD155 and CD276 in MelDCC 10 was performed with 1.5 μM Cas9 protein (Alt-R S.p. HiFi Cas9 nuclease V3), 1.8 μM *trans*-activating CRISPR RNA (Alt-R CRISPR–Cas9 tracrRNA), 1.8 μM CD276 AD crRNA (Hs.Cas9.CD276.1.AD), PVR AB crRNA (Hs.Cas9.PVR.1.AB) or control crRNA, 1.8 μM Alt-R Cas9 electroporation enhancer (all IDT) and 100,000 cells per transfection. Transfection was performed using the NEON transfection instrument (Thermo Fisher Scientific; settings: 1,600 V, 10-ms pulse width, three pulses).

### Induction of dedifferentiation in MelDCC lines

A total of 1×10^6^ MelDCC cells were seeded in T25 flasks 1 day before 500 U of IFNG (Peprotech) in PBS and 0.1% BSA was added to the culture medium. IFNG-containing medium was replaced every 2–3 days for 28 days. On day 28, the medium was switched to EV production medium (phenol-red-free and serum-free RPMI (Gibco), 2 mM l-glutamine and 1% P/S; all Pan-Biotech). Control cells were plated at 400,000 cells per T25 flask and the medium was switched to EV production medium after 48 h. After another 48 h, supernatants were collected from both IFNG-treated and control cells for NTA or sEV isolation by dUC. Flow cytometry was performed on seeded MelDCC at days 0, 28 and 30 and cell counts were recorded at harvest.

### CD8 T cell isolation

Peripheral blood mononuclear cells were prepared by density gradient centrifugation (60% Percoll solution, GE Healthcare) and cryoconserved. Upon thawing with 100 µg ml^−1^ DNAse I (Roche), cells were rested overnight at 2 × 10^6^ cells per well in a 96-well plate in 200 µl of RPMI 1640 medium with 10% FBS, 2 mM l-glutamine and 1% P/S (all Pan-Biotech), 100 mM HEPES (Sigma-Aldrich) and 0.1% 2-mercaptoethanol (Thermo Fisher Scientific). The next day, cells were pooled and CD8 T cells were isolated using the MojoSort Human CD8 T cell isolation kit (Biolegend) with LS columns (Miltenyi Biotec) and MACS buffer (PBS (Thermo Fisher Scientific), 0.5% BSA (Roche) and 2 mM EDTA (Thermo Fisher Scientific)) as per the manufacturer’s instructions.

### Testing of CD8 T cell-inhibitory function of sEVs

Polyclonal or MART1_27L26-35_-specific CD8 T cells were labeled with 2 µM CFSE (eBioscience) for 10 min at 37 °C in PBS with 1% FBS. The reaction was stopped with RPMI 1640 with 10% FBS (all Pan-Biotech). A total of 1 × 10^5^ CFSE-labeled CD8 T cells per well were stimulated on 96-well plates coated with 2 µg ml^−1^ anti-CD3 (OKT3) and 2 µg ml^−1^ CD28 (CD28.2) antibodies (both Biolegend) in 200 µl of PBS and overnight at 4 °C. sEVs were added 18 h before or after stimulation in a total volume of 200 µl and a ratio of 50:1, if not indicated otherwise. To block the CD155–TIGIT interaction, anti-TIGIT neutralizing antibody or the isotype control (as listed in Supplementary Table 8) was added at a final concentration of 10 µg ml^−1^. On day 4, cultures were restimulated for 4 h with 1× cell stimulation cocktail including protein transport inhibitors (eBioscience) and analyzed for CFSE dilution and INFG and GZMB production by flow cytometry.

### In vitro cytotoxicity assay

CD8 T cells from an HLA A02:01 healthy donor were expanded using artificial antigen-presenting cells (aAPCs) loaded with MART1_27L26-35_ as described previously^77^. To assess antigen-specific cytotoxicity, CD8 T cells were exposed to PBS or sEVs (50:1 ratio) before stimulation on anti-CD3/CD28-coated plates (2 µg ml^−1^). On day 4, CD8 T cells were mixed at a 1:1 ratio with 25,000 target and control cells and plated on a round-bottom 96-well plate. T2 target cells were labeled with CFSE (2 µM; eBioscience), preloaded with 10 µg ml^−1^ MART1_27L26-35_ peptide (1 h at 37 °C, 5% CO_2_) and washed. Unloaded T2 cells labeled with 2 µM CellTrace violet (eBioscience) were used as nontarget controls. After 24 h, T2 cells were analyzed by flow cytometry. Specific cytotoxic lysis was calculated as follows: (1 − (*r* with T cells/*r* without T cells) × 100), where *r* = (% CFSE^+^/% CellTrace violet^+^).

### PCLSs

PCLSs were prepared from tumor-distant tissue after tumor resection as described previously^78^. Lung lobes were filled with 4% low-melting-point agarose (Thermo Fisher Scientific), punched into 8-mm cores and sliced into 300-µm-thick slices using a Krumdieck tissue slicer (Alabama R&D). PCLSs were cultured in 48-well plates (one slice per well in 250 µl of DMEM F/12 (Thermo Fisher Scientific)) and treated with anti-CD3/CD28 beads (Thermo Fisher Scientific) 18 h before adding sEV or 5 µM pimecrolimus (Merck). Flow cytometry was performed 6 days after treatment on dissociated PCLS (six replicated per condition). Tissue was minced and dissociated with 2.2 mg ml^−1^ collagenase D and 0.055 mg ml^−1^ DNase I (Roche) in DMEM F/12 with 1% P/S (Thermo Fisher Scientific), shaken for 1 h at 37 °C and filtered through 100-µm sieves to obtain single-cell suspensions.

### Uptake of CFSE-labeled sEV by CD8 T cells

The 100K pellets were labeled with 20 µM CFSE (eBioscience) for 2 h at 37 °C. The reaction was stopped with 10% EV-free FBS (120,000*g* for 23 h) for 10 min at RT. PBS was processed identically as a control. CFSE-labeled sEVs or volume-matched PBS was added in a ratio of 50:1 and 10:1 to 1 × 10^5^ CD8 T cells stimulated for 18 h with 2 µg ml^−1^ plate-bound anti-CD3/CD28 antibodies (Supplementary Table 8). After 24 h at 37 °C and 5% CO_2_, cells were analyzed by flow cytometry for CFSE positivity.

### Flow cytometry

Single-cell suspensions of LNs or PCLSs, CD8–EV cocultures or MelDCC lines were incubated for 5 min at 4 °C with PBS and 10% AB serum (Bio-Rad) or human TruStain FcX receptor blocking solution (Biolegend) to reduce nonspecific antibody binding, stained with fluorescence-labeled antibodies for 30 min at 4 °C and washed once with PBS, 2% FBS and 0.01% NaN_3_. For intracellular cytokine staining, cells were fixed for 20 min at RT with FluoroFix (Biolegend) and permeabilized with intracellular staining permeabilization wash buffer (Biolegend). Intranuclear staining was conducted with the Foxp3 transcription factor staining buffer set (Thermo Fisher Scientific). Cells were stained for 30 min using the antibodies and isotype controls listed in Supplementary Table 8. Fixable viability dye eFluor 780 (eBioscience) or the Zombie NIR fixable viability kit (Biolegend) was used for live–dead cell discrimination. Cells were analyzed on an LSR II, FACSCelesta, FACSymphonyA5 SORP or Cytoflex (Beckman Coulter) machine and data were analyzed with FloJo (version 10.8.1; Tree Star). Sorting of CD155 and CD276 MelDCC lines after CD155 and CD276 CRIPSR–Cas9 knockout was performed with a FACSAria IIu cell sorter (BD Bioscience).

### Statistics and reproducibility

No statistical method was used to predetermine the sample size. For RNA-seq experiments, sample size was determined by the availability of high-quality RNA from DCCs and control cells in LNs to ensure sufficient power for meaningful patterns. For other experiments, sample numbers were based on practical considerations to reliably address primary objectives. Post hoc assessments confirmed that sample numbers were adequate for valid results.

To ensure reproducibility, independent replicate experiments were performed, including biological and technical replicates where applicable. Technical replicates were used to assess measurement accuracy, assay reproducibility and technical variability. Data were analyzed using multiple statistical approaches to confirm robustness. All replication attempts were successful, with consistent results across biological samples and experimental conditions. WB analyses were performed in at least two independent biological replicates and microscopy images were obtained from multiple replicates, with consistent findings observed.

Statistical analyses were conducted using GraphPad Prism (version 9.3.1) and R (version 4.1.0). Data distribution was assumed to be normal but this was not formally tested. Data distribution (individual data points) is shown when possible and always for *n* ≤ 10. Differences in mean values were assessed using Student’s *t*-test or a one-way ANOVA with post hoc tests. Univariable, multivariable and survival analyses were performed using Cox regression and the Mantel–Cox log-rank test. All tests were two-sided, with *P* < 0.05 considered statistically significant. DCCD values were log-transformed as DCCD = log_10_(DCCD + 1).

Patient samples were included in the study according to availability, which was determined by factors such as the number of isolated cells, RNA quality and available survival data. No preassigned groupings or patient selections were made before data analysis. As the study did not involve controlled experimental conditions or treatment interventions, random allocation of samples was not applicable. No formal covariate-based randomization was performed, as the study addressed the natural variability within the available patient samples. For in vitro experiments involving cell lines, sEVs and CD8 T cells, the samples were first pooled and then allocated to the various experimental conditions.

All patient samples were pseudonymized according to EU General Data Protection Regulation (GDPR) and pseudonyms linked clinical and outcome data. Investigators were blinded to patient disease progression and clinical status until final bioinformatics analysis. This ensured that the data collection and initial analysis were conducted without bias related to disease state. For post-RNA-seq analyses, such as survival analysis, reidentification was necessary to link clinical outcomes to the molecular data. Blinding was not feasible for survival analysis, as patient outcomes were required to interpret these findings; however, bias was minimized by the use of pseudonymized data during the initial steps of the experiment. For cell line, sEV and T cell experiments, investigators were not blinded because different treatments were required for separate groups.

### Reporting summary

Further information on research design is available in the Nature Portfolio Reporting Summary linked to this article.

## Supplementary information

Supplementary Information Summary of flow cytometry gating strategies.

Reporting Summary

Supplementary Tables 1–9 Supplementary Tables 1–9.

## Source data

Source Data Fig. 1 Data values.

Source Data Fig. 2 Data values.

Source Data Fig. 3 Data values.

Source Data Fig. 4 Data values.

Source Data Fig. 5 Data values.

Source Data Fig. 6 Data values.

Source Data Table 2 Data values.

Source Data Figs. 5 and 6 and Extended Data Figs. 7, 9 and 10 Unprocessed western blots for all Figs. 5 and 6 and Extended Data Figs. 7, 9 and 10.

Source Data Extended Data Fig. 1 Data values.

Source Data Extended Data Fig. 2 Data values.

Source Data Extended Data Fig. 3 Data values.

Source Data Extended Data Fig. 4 Data values.

Source Data Extended Data Fig. 5 Data values.

Source Data Extended Data Fig. 6 Data values.

Source Data Extended Data Fig. 7 Data values.

Source Data Extended Data Fig. 8 Data values.

Source Data Extended Data Fig. 9 Data values.

Source Data Extended Data Fig. 10 Data values.

## Data Availability

The RNA-seq data generated in this study were deposited to the European Genome–Phenome Archive (EGA) under accession number EGAS00001006702. The MS proteomics data were deposited to the ProteomeXchange Consortium through the PRIDE^79^ partner repository with the dataset identifier PXD059510. Access to patient-derived material and raw sequencing data is restricted because of patient consent and compliance with the GDPR. To request access, a formal application must be submitted to the Data Access Committee (DAC) associated with the dataset through the EGA. Access requests are reviewed and processed as promptly as possible but remain subject to DAC approval. To obtain processed data in the form of count tables, please contact the corresponding authors. Previously published RNA-seq data reanalyzed in this study are available from the Gene Expression Omnibus or EGA under the following accession codes: Karras_Braf and Karras_NRAS (GSE207592), Belote (GSE151091), Wouters (GSE134432), Jerby-Arnon (GSE115978), Pozniak (EGAD00001010921) and Cheng (EGAS00001002927). Cancer Cell Line Encyclopedia data were obtained online (https://data.broadinstitute.org/ccle/CCLE_RNAseq_rsem_genes_tpm_20180929.txt.gz) and Human Cell Atlas data were retrieved from figshare (https://figshare.com/articles/dataset/Tabula_Sapiens_release_1_0/14267219). For datasets where accession codes were not available, count tables or gene expression signatures were obtained from the supplementary materials of the respective publications or directly from the authors upon request. All other data supporting the findings of this study are available within the article and Supplementary Information or from the corresponding authors upon reasonable request. Source data are provided with this paper.

## Code availability

All data analysis and processing were executed through the use of published software or packages, the details of which are referred to within Methods. No novel code or mathematical algorithms were generated by this study.
