# Supplementary material for: MCSP+ metastasis founder cells activate immunosuppression early in human melanoma metastatic colonization
Source: Nat Cancer. 2025 May 16;6(6):1017–34. doi: 10.1038/s43018-025-00963-w (PMC12202500; doi:10.1038/s43018-025-00963-w)
Supplement: Supplementary file 1 — Summary of flow cytometry gating strategies. [file 43018_2025_963_MOESM1_ESM.pdf]

nature cancer

Supplementary information

https://doi.org/10.1038/s43018-025-00963-w

MCSP$^+$ metastasis founder cells activate immunosuppression early in human melanoma metastatic colonization

In the format provided by the authors and unedited

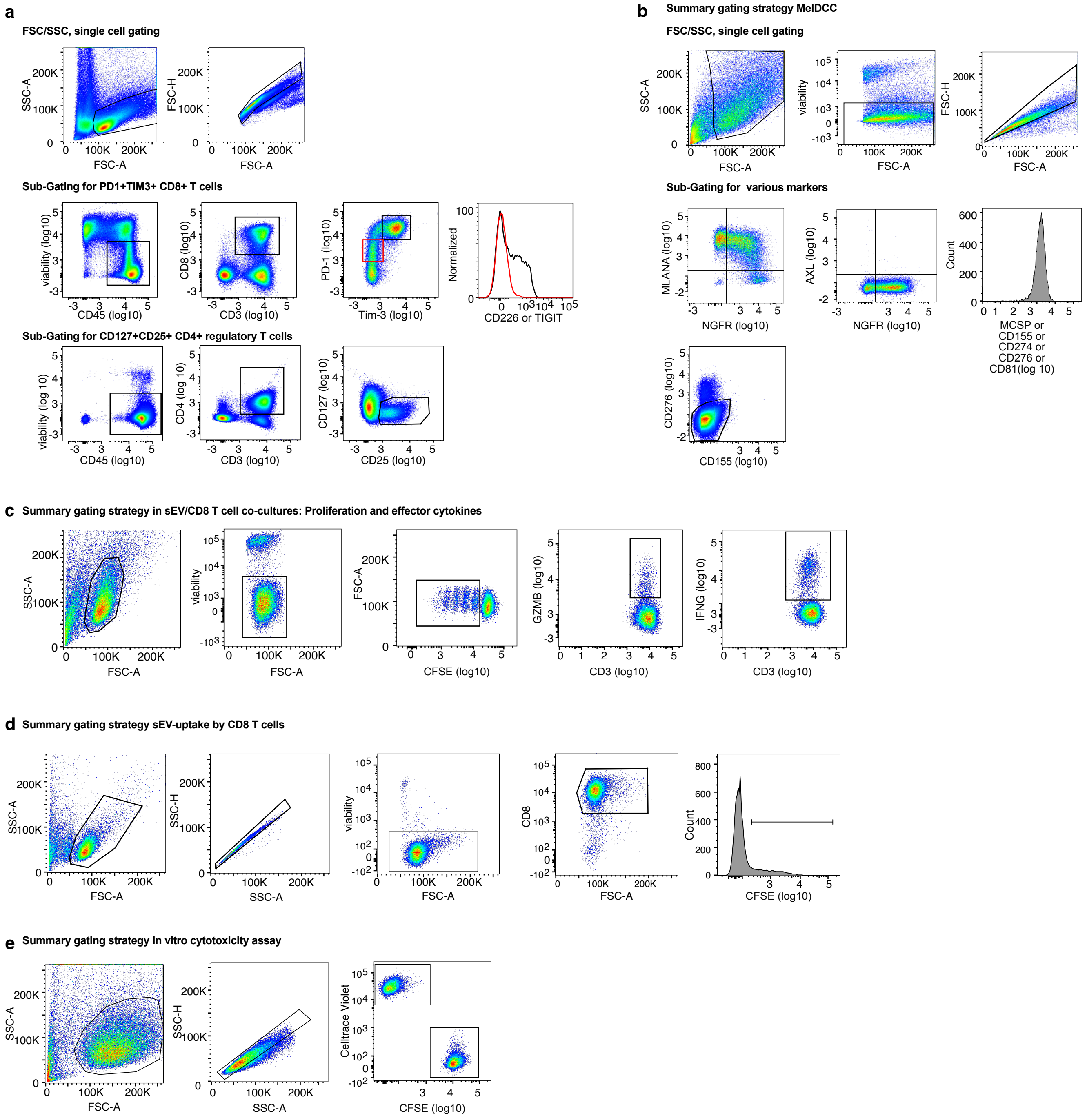

Supplementary Figure 1. Flow cytometry gating strategies. a, Gating strategy for the analysis of CD127+CD25+CD4+ T cells, PD1+Tim3+ CD8 T cells and their expression of indicated protein markers, in lymph nodes of melanoma and non-melanoma patients. **b,** Gating strategy for the analysis of MelDCC and their expression of indicated protein markers. **c,** Gating strategy for the analysis of CD8 T cell proliferation and effector cytokines as a function of CFSE-dilution. **d,** Gating strategy used for the analysis of sEV-uptake by CD8 T cells. **e,** Gating strategy for the detection of Celltrace Violet- and CFSE-labeled T2 cells in *in vitro* cytotoxicity assays.
