# Supplementary material for: MCSP+ metastasis founder cells activate immunosuppression early in human melanoma metastatic colonization
Source: Nat Cancer. 2025 May 16;6(6):1017–34. doi: 10.1038/s43018-025-00963-w (PMC12202500; doi:10.1038/s43018-025-00963-w)
Supplement: Supplementary file 2 — Reporting Summary [file 43018_2025_963_MOESM2_ESM.pdf]

nature portfolio

Corresponding author(s): Melanie Werner-Klein, Christoph Andreas Klein

Last updated by author(s): 24/02/2025



# Reporting Summary

Nature Portfolio wishes to improve the reproducibility of the work that we publish. This form provides structure for consistency and transparency in reporting. For further information on Nature Portfolio policies, see our Editorial Policies and the Editorial Policy Checklist.

## Statistics

For all statistical analyses, confirm that the following items are present in the figure legend, table legend, main text, or Methods section.

| n/a | Confirmed | |
|---|---|---|
| ☐ | ☒ | The exact sample size (*n*) for each experimental group/condition, given as a discrete number and unit of measurement |
| ☐ | ☒ | A statement on whether measurements were taken from distinct samples or whether the same sample was measured repeatedly |
| ☐ | ☒ | The statistical test(s) used AND whether they are one- or two-sided<br>*Only common tests should be described solely by name; describe more complex techniques in the Methods section.* |
| ☐ | ☒ | A description of all covariates tested |
| ☐ | ☒ | A description of any assumptions or corrections, such as tests of normality and adjustment for multiple comparisons |
| ☐ | ☒ | A full description of the statistical parameters including central tendency (e.g. means) or other basic estimates (e.g. regression coefficient) AND variation (e.g. standard deviation) or associated estimates of uncertainty (e.g. confidence intervals) |
| ☐ | ☒ | For null hypothesis testing, the test statistic (e.g. *F*, *t*, *r*) with confidence intervals, effect sizes, degrees of freedom and *P* value noted<br>*Give P values as exact values whenever suitable.* |
| ☒ | ☐ | For Bayesian analysis, information on the choice of priors and Markov chain Monte Carlo settings |
| ☒ | ☐ | For hierarchical and complex designs, identification of the appropriate level for tests and full reporting of outcomes |
| ☐ | ☒ | Estimates of effect sizes (e.g. Cohen's *d*, Pearson's *r*), indicating how they were calculated |

*Our web collection on statistics for biologists contains articles on many of the points above.*

## Software and code

Policy information about availability of computer code

| | |
|---|---|
| Data collection | No software or code was used to collect data |
| Data analysis | FACS samples were analysed with FlowJo (v10.8.1,Tree Star). MS/MS spectra wer analyzed using ProteinPilot (v5.0), PeakView (v2.1) and SWATH MicroApp 2.0. Western Blot images were analyzed with Image Lab (v6.1, Bio-Rad). NTA-analysis was perfomed using the ZetaView software (v8.05.14 SP7) and FlowJo (v10.8.1, TreeStar). Transmission electron microscopy images were acquired using the SerialEM software package (Mastronarde et al 2005). Data analyis, statistical testing and visualization was performed using the GraphPad Prism (v9.3.1) software (GraphPad Software, Inc.) and R (v4.1.0). Immunofluorescent staining were evaluated with ImageJ/Fiji.For RNA sequencing quality control and alignment FastQC (v0.11.5), MulitQC 1.12 and STAR (v2.6.1c) was used. Gene counts were calculated with RSEM (v1.3.1). Data analysis and visualization of processed RNAseq data was performed using Seurat (v4.1.0), ), Bluster (v1.2.1), enrichR(v3.1), slingshot (v2.0.0), gam (v1.20.1), scWGCNA (v0.0.0.9000), WGCNA (v1.70.3), SCENIC(v1.2.4 ), CONICSmat (v0.0.0.0.1),clusterExperiment(v2.12.0) ,ElPiGraph.R(v1.0.0),AUCell(v1.14.0),ggplot2(v3.4.0). For CNA analysis genomic coordinates were analyzed with the LowPass bioinformatics pipeline of Menarini Silicon Biosystems or HIENA (Fraunhofer ITEM-R and submitted to Progenetix (v4.0). For CODEX analysis of LN with high DCCD ImageJ (Fiji, version 2.0.0). was used. For the LN with low DCCD, the overlay images were created in QuPath 0.5.1. |

For manuscripts utilizing custom algorithms or software that are central to the research but not yet described in published literature, software must be made available to editors and reviewers. We strongly encourage code deposition in a community repository (e.g. GitHub). See the Nature Portfolio guidelines for submitting code & software for further information.

# Data

Policy information about availability of data

All manuscripts must include a data availability statement. This statement should provide the following information, where applicable:

- Accession codes, unique identifiers, or web links for publicly available datasets
- A description of any restrictions on data availability
- For clinical datasets or third party data, please ensure that the statement adheres to our policy

The RNA sequencing data generated in this study have been deposited at the European Genome-Phenome Archive (EGA) under accession number EGAS00001006702. The mass spectrometry proteomics data have been deposited to the ProteomeXchange Consortium via the PRIDE 80 partner repository with the dataset identifier PXD059510. Access to patient-derived material and raw sequencing data is restricted due to patient consent and compliance with the General Data Protection Regulation (GDPR). Previously published RNA sequencing data re-analyzed in this study are available under the following accession codes: Karras_Braf and Karras_NRAS (GSE207592), Belote (GSE151091), Wouters (GSE134432), Jerby-Arnon (GSE115978), and Pozniak (EGAD00001010921). CCLE data were obtained from https://data.broadinstitute.org/ccle/CCLE_RNAseq_rsem_genes_tpm_20180929.txt.gz, and the Human Cell Atlas data were retrieved from https://figshare.com/articles/dataset/Tabula_Sapiens_release_1_0/14267219. For datasets where accession codes were not available, count tables or gene expression signatures were obtained from the supplementary materials of the respective publications or directly from the authors upon request. All other data supporting the findings of this study are available within the article, its Extended Data, and source files, or from the corresponding author upon reasonable request.

# Research involving human participants, their data, or biological material

Policy information about studies with human participants or human data. See also policy information about sex, gender (identity/presentation), and sexual orientation and race, ethnicity and racism.

| | |
|---|---|
| Reporting on sex and gender | The survival analysis is based on 192 female and 243 male participants. Gender of participants was recorded based on self-reporting. Inclusion criteria for the study was not based on gender. Gender was included as parameter in multivariable analysis. |
| Reporting on race, ethnicity, or other socially relevant groupings | Inclusion criteria for the study was not based on race, ethnicity or other socially relevant groupings. |
| Population characteristics | Human disseminated cancer cells were obtained from sentinel or regional lymph nodes of melanoma patients. Skin draining control lymph nodes were obtained from non-melanoma patients. Patients were included independent of gender or age. |
| Recruitment | Clinicians recruited patients according to the above mentioned population characteristics. All patients signed informed consent. No other selection criteria were applied. Participants did not receive any compensation for their involvement in this study. |
| Ethics oversight | The study complies with all relevant ethical regulations regarding the use of patient material. Human disseminated cancer cells were obtained from sentinel or regional LNs of melanoma patients and control skin-draining LNs were obtained from non-melanoma patients (ethics vote 07-079 and 18-948-101, ethics committee of the University of Regensburg. Human peripheral blood mononuclear cells were obtained from a healthy donor (ethics vote 20-1991-101, ethics committee of the University of Regensburg) and human tumor-free lung samples from patients with lung cancer (ethics vote 2701-2015, ethic committee Medical School Hannover. Written informed consent was obtained from all patients and healthy participants. Participantsprovided explicit consent for the inclusion of information listed in Table 2 and the source data. Participants did not receive any compensation for their involvement in this study. |

Note that full information on the approval of the study protocol must also be provided in the manuscript.

# Field-specific reporting

Please select the one below that is the best fit for your research. If you are not sure, read the appropriate sections before making your selection.

☒ Life sciences ☐ Behavioural & social sciences ☐ Ecological, evolutionary & environmental sciences

For a reference copy of the document with all sections, see nature.com/documents/nr-reporting-summary-flat.pdf

# Life sciences study design

All studies must disclose on these points even when the disclosure is negative.

| | |
|---|---|
| Sample size | No statistical method was used to pre-determine the sample size. For RNA-seq experiments, sample size was determined by the availability of high-quality RNA from DCC and control cells in lymph nodes to ensure sufficient power for meaningful patterns. For other experiments, sample sizes were based on practical considerations to reliably address primary objectives. Post hoc assessments confirmed that sample sizes were adequate for valid results. |

Data exclusions

Single cells (DCC or control cells) with insufficient RNA-quality were excluded. For scRNAseq, cells with less than 50,000 counts, mitochondrial gene counts of more than 70% or less than 1,000 expressed genes were excluded, and only genes expressed in at least 3 cells were kept.

Replication

To ensure reproducibility, independent replicate experiments were performed, including biological and technical replicates where applicable. Technical replicates were used to assess measurement accuracy, assay reproducibility, and technical variability. Data were analyzed using multiple statistical approaches to confirm robustness. All replication attempts were successful, with consistent results across biological samples and experimental conditions. Western blot analyses were performed in at least two independent biological replicates, and microscopy images were obtained from multiple replicates, with consistent findings observed.

Randomization

Patient samples were included in the study based on availability, which was determined by factors such as the number of isolated cells, RNA quality, and available survival data. No pre-assigned groupings were made prior to data analysis. As the study did not involve controlled experimental conditions or treatment interventions, random allocation of samples was not applicable. No formal covariate-based randomization was performed, as the focus of this study was to evaluate natural variability within the available patient samples. For in vitro experiments involving cell lines, sEVs, and CD8 T cells, the samples were first pooled and then allocated to the various experimental conditions.

Blinding

All patient samples were pseudonymized according to EU-GDPR and pseudonyms linked clinical and outcome data. Investigators were blinded to the patient's disease progression and clinical status until final bioinformatics analysis. This ensured that the data collection and initial analysis were conducted without bias related to disease state. For post-RNA sequencing analyses, such as patient survival analysis, re-identification was necessary to link clinical outcomes to the molecular data. Blinding was not feasible for survival analysis, as patient outcomes were required to interpret these findings, but bias was minimized by the use of pseudonymized data during the initial steps of the experiment. For cell line/sEV/T cells experiments investigators were not blinded since different treatments were required for separate groups.

# Reporting for specific materials, systems and methods

We require information from authors about some types of materials, experimental systems and methods used in many studies. Here, indicate whether each material, system or method listed is relevant to your study. If you are not sure if a list item applies to your research, read the appropriate section before selecting a response.

## Materials & experimental systems

| n/a | Involved in the study |
|---|---|
| ☐ | ☒ Antibodies |
| ☐ | ☒ Eukaryotic cell lines |
| ☒ | ☐ Palaeontology and archaeology |
| ☒ | ☐ Animals and other organisms |
| ☒ | ☐ Clinical data |
| ☒ | ☐ Dual use research of concern |
| ☒ | ☐ Plants |

## Methods

| n/a | Involved in the study |
|---|---|
| ☒ | ☐ ChIP-seq |
| ☐ | ☒ Flow cytometry |
| ☒ | ☐ MRI-based neuroimaging |

## Antibodies

Antibodies used

See also Supplementary Table 8:
Reagent/Type Used for Target Conjugation clone stock conc. Dilution or final concentration Cat # Supplier

Antibody Quantitative Immunocytology, IHC gp100 none HMB45 N.A. 1:100 M063401-2 Agilent DAKO
Antibody Quantitative Immunocytology, IHC, CODEX, Flowcytometry MCSP none 9.2.27 0.5 mg/ml 1:50 554275 BD Biosciences
TruStain FC Immunofluorescent staining, Flowcytometry, Blocking FC Receptors none N.A. N.A. 1:20 422302 Biolegend
Antibody Multicolor-immunofluorescent staining on adhesion slides MCSP none LHM2 1 mg/ml 1:176 20156 Abcam
Sec. Antibody Multicolor-immunofluorescent staining on adhesion slides mouse IgG1 AF546 polyclonal 2 mg/ml 1:150 A-21123 Invitrogen
Mouse Serum Multicolor-immunofluorescent staining on adhesion slides blocking free binding sites of anti-mouse IgG1 none N.A. N.A. 1:20 X091001-8 DAKO
Antibody Multicolor-immunofluorescent staining on adhesion slides MelanA Biotin A-103 0.1 mg/ml 1:100 MA5-14168 Invitrogen
Antibody Multicolor-immunofluorescent staining on adhesion slides CD74 APC LN2 0.15 mg/ml 1:20 326812 Biolegend
Streptavidin Multicolor-immunofluorescent staining on adhesion slides Biotin AF488 N.A. 2 mg/ml 1:250 S11223 Invitrogen
DAPI Multicolor-immunofluorescent staining on adhesion slides dsDNA none N.A. 5 mg/ml 1:25,000 10236276001 Roche
Antibody IHC MelanA none A-103 N.A. 1:600 M7196 Agilent DAKO
Antibody CODEX MelanA oligo Tag A103+M2-7C10+M29e3 1 mg/ml 1:50 NBP2-34546-0.1mg Novus Biologicals
Antibody CODEX CD3 oligo Tag MRQ-39 1 mg/ml 1:50 custom order Cell Marque
Antibody CODEX IFNy oligo Tag clone not disclosed N.A. 1:100 4250062 Akoya Biosciences
Antibody CODEX Tim3 oligo Tag EPR22241 0.978 - 1.045 mg/mL 1:50 ab242080 Abcam
Antibody CODEX CD47 oligo Tag polyclonal 0.2 mg/mL 1:50 AF4670 R&D systems
Antibody WB CD81 none B-11 0.2 mg/ml 1:10,000 sc-166029 Santa Cruz Biotechnology
Antibody WB GAPDH none 6C5 0.1 mg/ml 1:1,000 sc-32233 Santa Cruz Biotechnology
Antibody WB IL20Rα none EE09 0.1 mg/ml 1:500 sc-80065 Santa Cruz Biotechnology
Antibody WB Calnexin none 37/Calnexin 0.25 mg/ml 1:1,000 610524 BD Biosciences

Antibody WB HSP70 none 7/Hsp70 0.25 mg/ml 1:1,000 610607 BD Biosciences
Antibody WB TSG101 none 51/TSG101 0.25 mg/ml 1:1,000 612697 BD Biosciences
Antibody WB CD39 none EPR20627 0.613 mg/ml 1:1,000 ab223842 Abcam
Antibody WB CD73 none EPR6114 2.235 mg/ml 1:1,000 ab133582 Abcam
Antibody WB CD200 none EPR22412-229 0.507 mg/ml 1:1,000 ab254193 Abcam
Antibody WB CD276 none EPR20115 0.516 mg/ml 1:5,000 ab219648 Abcam
Antibody WB PD-L1 none EPR19759 0.443 mg/ml 1:1,000 ab213524 Abcam
Antibody WB CD155 none EPR17302 0.151 mg/ml 1:2,000 ab205304 Abcam
Antibody WB GRP94 none B-11 1 mg/ml 1:1,000 ADI-SPA-850-F Enzo Life Sciences
Antibody WB Albumin none JF32-10 1 mg/ml 1:1,000 MA5-3253 Invitrogen
Antibody WB ACLY none polyclonal rabbit 0.037 mg/ml 1:1,000 4332S Cell Signaling
Antibody WB Fibronectin none E5H6X 0.1 mg/ml 1:1,000 26836T Cell Signaling
Antibody WB Histone H2A none polyclonal 0.01 mg/ml 1:1,000 2578S Cell Signaling
Antibody WB mouse IgG HRP polyclonal N.A. 1:10,000 A8924-.5ML Sigma Aldrich
Antibody WB rabbit IgG1 HRP polyclonal N.A. 1:10,000 A0545-1ML Sigma Aldrich
Antibody WB rat IgG1 HRP polyclonal N.A. 1:10,000 A9037-1ML Sigma Aldrich
Antibody NTA CD81 PE/Dazzle 594 5A6 lot-specific, use of individual lots not documented 1:10 349519 Biolegend
CellMaskGreen NTA Plasma membrane proprietary N.A. N.A. 1:1,000 C37608 Invitrogen
CFSE Flowcytometry Intracellular proteins none N.A. 10 mM 1:500/1:5,000 65-0850-84 eBioscience
Antibody polyclonal CD8 T cell stimulation CD3 none OKT3 0.5 mg/ml 1:250 317302 Biolegend
Antibody polyclonal CD8 T cell stimulation CD28 none CD28.2 0.5 mg/ml 1:250 302902 Biolegend
Antibody anti-TIGIT blockade TIGIT none clone not disclosed 3.01 mg/ml 10 ug/ml 71340 BPS Bioscience
Human IgG1 Isotype control for anti-TIGIT blocking antibody N.A. none QA16A12 1 mg/ml 10 ug/ml 403501 Biolegend
CellTrace Violet Flowcytometry Intracellular proteins none N.A. 5 mM 1:2,500 C34557 Invitrogen
human AB Serum Flowcytometry blocking of human FC Receptors none N.A. N.A. 1:10 805135 Biorad
Antibody Flowcytometry CD45 FITC HI30 lot-specific, use of individual lots not documented 1/2 of concentration indicated on data-sheet 304006 Biolegend
Antibody Flowcytometry CD45 AF488 HI30 lot-specific, use of individual lots not documented 1/2 of concentration indicated on data-sheet 304017 Biolegend
Antibody Flowcytometry CD45 PerCP HI30 lot-specific, use of individual lots not documented 1/2 of concentration indicated on data-sheet 304026 Biolegend
Antibody Flowcytometry CD3 AF700 SK7 lot-specific, use of individual lots not documented 1/2 of concentration indicated on data-sheet 344822 Biolegend
Antibody Flowcytometry CD3 AF700 UCHT1 lot-specific, use of individual lots not documented 1/2 of concentration indicated on data-sheet 300424 Biolegend
Antibody Flowcytometry CD3 PerCP/Cyanine5.5 SK7 lot-specific, use of individual lots not documented 1/2 of concentration indicated on data-sheet 344808 Biolegend
Antibody Flowcytometry CD3 PerCP/Cyanine5.5 HIT3a lot-specific, use of individual lots not documented 1/2 of concentration indicated on data-sheet 300328 Biolegend
Antibody Flowcytometry CD4 BV650 RPA-T4 lot-specific, use of individual lots not documented 1/2 of concentration indicated on data-sheet 300536 Biolegend
Antibody Flowcytometry CD4 AF700 SK3 lot-specific, use of individual lots not documented 1/2 of concentration indicated on data-sheet 344622 Biolegend
Antibody Flowcytometry CD8 AF700 HIT8α lot-specific, use of individual lots not documented 1/2 of concentration indicated on data-sheet 300920 Biolegend
Antibody Flowcytometry CD8 BV510 RPA-T8 lot-specific, use of individual lots not documented 1/2 of concentration indicated on data-sheet 301048 Biolegend
Antibody Flowcytometry CD8 BV 421 RPA-T8 lot-specific, use of individual lots not documented 1/2 of concentration indicated on data-sheet 301036 Biolegend
Antibody Flowcytometry CD25 PE BC96 lot-specific, use of individual lots not documented 1/2 of concentration indicated on data-sheet 302606 Biolegend
Antibody Flowcytometry CD127 PE-Dazzle 594 A019D5 lot-specific, use of individual lots not documented 1/2 of concentration indicated on data-sheet 351336 Biolegend
Antibody Flowcytometry Ki-67 APC Ki-67 lot-specific, use of individual lots not documented 1/2 of concentration indicated on data-sheet 350514 Biolegend
Antibody Flowcytometry IFNG PE 4S.B3 lot-specific, use of individual lots not documented 1/2 of concentration indicated on data-sheet 502509 Biolegend
Antibody Flowcytometry GZMB AF647 GB11 lot-specific, use of individual lots not documented 1/2 of concentration indicated on data-sheet 515406 Biolegend
Antibody Flowcytometry CD226 PE/Cyanine7 DNAM-1 lot-specific, use of individual lots not documented 1/2 of concentration indicated on data-sheet 128812 Biolegend
Antibody Flowcytometry TIGIT BV421 VSTM3 lot-specific, use of individual lots not documented 1/2 of concentration indicated on data-sheet 372710 Biolegend
Antibody Flowcytometry TIM-3 BV510 F38-2E2 lot-specific, use of individual lots not documented 1/2 of concentration indicated on data-sheet 345030 Biolegend
Antibody Flowcytometry PD-1 BV711 EH12.2H7 lot-specific, use of individual lots not documented 1/2 of concentration indicated on data-sheet 329928 Biolegend
Antibody Flowcytometry PD-1 BV421 EH12.2H7 lot-specific, use of individual lots not documented 1/2 of concentration indicated on data-sheet 329920 Biolegend
Antibody Flowcytometry CD155 PE SKII.4 lot-specific, use of individual lots not documented 1/2 of concentration indicated on data-sheet 337610 Biolegend
Antibody Flowcytometry CD155 PE/Cyanine7 SKII.4 lot-specific, use of individual lots not documented 1/2 of concentration indicated on data-sheet 337614 Biolegend
Antibody Flowcytometry CD271 PE ME20.4 lot-specific, use of individual lots not documented 1/2 of concentration indicated on data-sheet 345106 Biolegend
Antibody Flowcytometry CD274 BV711 29E.2A3 lot-specific, use of individual lots not documented 1/2 of concentration indicated on

data-sheet 329722 Biolegend
Antibody Flowcytometry CD276 APC MIH42 lot-specific, use of individual lots not documented 1/2 of concentration indicated on data-sheet 351006 Biolegend
Antibody Flowcytometry CD8 BUV805 HIT8α lot-specific, use of individual lots not documented 1/2 of concentration indicated on data-sheet 742030 BD Biosciences
Antibody Flowcytometry TIM-3 BUV615 7D3 lot-specific, use of individual lots not documented 1/2 of concentration indicated on data-sheet 752363 BD Biosciences
Antibody Flowcytometry TNF BUV395 Mab11 lot-specific, use of individual lots not documented 1/2 of concentration indicated on data-sheet 563996 BD Biosciences
Antibody Flowcytometry CD276 PerCP-eFluor710 7-517 lot-specific, use of individual lots not documented 1/2 of concentration indicated on data-sheet 46-2769-42 Thermo Fisher Scientific
Antibody Flowcytometry AXL Super Bright 436 DS7HAXL lot-specific, use of individual lots not documented 1/2 of concentration indicated on data-sheet 62-1087-42 Thermo Fisher Scientific
Antibody Flowcytometry MelanA AF647 A103 lot-specific, use of individual lots not documented 1/2 of concentration indicated on data-sheet sc-20032 Santa Cruz
Antibody Flowcytometry MCSP FITC EP-1 lot-specific, use of individual lots not documented 1/2 of concentration indicated on data-sheet 130-098-794 Miltenyi
Isotype ctrl. Flowcytometry mouse IgG1, κ BUV395 X40 lot-specific, use of individual lots not documented matched to antibody 563547 BD Biosciences
Isotype ctrl. Flowcytometry mouse IgG1, κ BUV615 X40 lot-specific, use of individual lots not documented matched to antibody 612986 BD Biosciences
Isotype ctrl. Flowcytometry mouse IgG1, κ PE MOPC-21 lot-specific, use of individual lots not documented matched to antibody 400111 Biolegend
Isotype ctrl. Flowcytometry mouse IgG1, κ BV711 MOPC-21 lot-specific, use of individual lots not documented matched to antibody 400167 Biolegend
Isotype ctrl. Flowcytometry mouse IgG1, κ PE/Cyanine7 MOPC-21 lot-specific, use of individual lots not documented matched to antibody 400125 Biolegend
Isotype ctrl. Flowcytometry mouse IgG1, κ BV421 MOPC-21 lot-specific, use of individual lots not documented matched to antibody 400158 Biolegend
Isotype ctrl. Flowcytometry mouse IgG1, κ APC MOPC-21 lot-specific, use of individual lots not documented matched to antibody 400121 Biolegend
Isotype ctrl. Flowcytometry mouse IgG1, κ AF647 MOPC-21 lot-specific, use of individual lots not documented matched to antibody 400136 Biolegend
Isotype ctrl. Flowcytometry mouse IgG2b, κ BV711 MPC-11 lot-specific, use of individual lots not documented matched to antibody 400354 Biolegend
Isotype ctrl. Flowcytometry mouse IgG2a, κ BV421 MOPC-173 lot-specific, use of individual lots not documented matched to antibody 400260 Biolegend
Isotype ctrl. Flowcytometry mouse IgG1, κ PerCP-eFluor710 P3.6.2.8.1 lot-specific, use of individual lots not documented matched to antibody 46-4714-82 Thermo Fisher Scientific
Isotype ctrl. Flowcytometry mouse IgG1, κ BV510 MOPC-21 lot-specific, use of individual lots not documented matched to antibody 400172 Biolegend
Isotype ctrl. Flowcytometry mouse IgG1, κ Super Bright 436 P3.6.2.8.1 lot-specific, use of individual lots not documented matched to antibody 62-4714-80 Thermo Fisher Scientific
Live/dead cells discrimination, Fixable Viability Dye Flowcytometry dead cells eFluor 780 N.A. N.A. 1:2000 65-0865-18 eBioscience
Live/dead cells discrimination, Zombie Flowcytometry dead cells NIR N.A. N.A. 1:200 423105 Biolegend

Validation

The antibodies used for immunocytology in this study were either previously validated (Werner-Klein et al Nature Communications 2018; Ulmer et al. PLoSMedicine 2014) or validated using control cells with or without antigen-expression. Antibodies for FACS analysis were commercially available and have been validated by the manufacturere for the application used in the study. Key features were verified by subgating strategies. True positive staining was determined by using isotype controls.

## Eukaryotic cell lines

Policy information about cell lines and Sex and Gender in Research

Cell line source(s)

MelDCC lines were established from xenografts derived from DCC from sentinel or regional LN of melanoma patients undergoing lymph node exstirpation at the university clinics of Regensburg. (Werner-Klein et al Nature Communications 2018). TThe NCI-H1975 cell line was obtained from ATCC and the HeLa cell line from Prof. Hehlgans, Leibniz Institute for Immunotherapy. Both lines were authenticated using STR profiling.

Authentication

The patient origin of MelDCC lines was verified by STR analysis (Cell-ID™, Promega), their melanoma origin by a human pathologist and their aberrant genotype by CGH. HeLa and NCI-H1975 were also verified by STR analysis.

Mycoplasma contamination

All cell lines were routinely tested for mycoplasma and were found to be negative.

Commonly misidentified lines
(See ICLAC register)

No misidentified lines were used in this study.

## Palaeontology and Archaeology

Specimen provenance

n.a.

Specimen deposition | n.a.

Dating methods | n.a.

☐ Tick this box to confirm that the raw and calibrated dates are available in the paper or in Supplementary Information.

Ethics oversight | n.a.

Note that full information on the approval of the study protocol must also be provided in the manuscript.

## Animals and other research organisms

Policy information about studies involving animals; ARRIVE guidelines recommended for reporting animal research, and Sex and Gender in Research

| | |
|---|---|
| Laboratory animals | n.a. |
| Wild animals | n.a. |
| Reporting on sex | n.a. |
| Field-collected samples | n.a. |
| Ethics oversight | n.a. |

Note that full information on the approval of the study protocol must also be provided in the manuscript.

## Dual use research of concern

Policy information about dual use research of concern

### Hazards

Could the accidental, deliberate or reckless misuse of agents or technologies generated in the work, or the application of information presented in the manuscript, pose a threat to:

| No | Yes | |
|---|---|---|
| ☒ | ☐ | Public health |
| ☒ | ☐ | National security |
| ☒ | ☐ | Crops and/or livestock |
| ☒ | ☐ | Ecosystems |
| ☒ | ☐ | Any other significant area |

### Experiments of concern

Does the work involve any of these experiments of concern:

| No | Yes | |
|---|---|---|
| ☒ | ☐ | Demonstrate how to render a vaccine ineffective |
| ☒ | ☐ | Confer resistance to therapeutically useful antibiotics or antiviral agents |
| ☒ | ☐ | Enhance the virulence of a pathogen or render a nonpathogen virulent |
| ☒ | ☐ | Increase transmissibility of a pathogen |
| ☒ | ☐ | Alter the host range of a pathogen |
| ☒ | ☐ | Enable evasion of diagnostic/detection modalities |
| ☒ | ☐ | Enable the weaponization of a biological agent or toxin |
| ☒ | ☐ | Any other potentially harmful combination of experiments and agents |

# Plants

| | |
|---|---|
| Seed stocks | n.a. |
| Novel plant genotypes | n.a. |
| Authentication | n.a. |

# ChIP-seq

## Data deposition

☐ Confirm that both raw and final processed data have been deposited in a public database such as GEO.

☐ Confirm that you have deposited or provided access to graph files (e.g. BED files) for the called peaks.

| | |
|---|---|
| Data access links<br>*May remain private before publication.* | n.a. |
| Files in database submission | n.a. |
| Genome browser session<br>(e.g. UCSC) | n.a. |

## Methodology

| | |
|---|---|
| Replicates | n.a. |
| Sequencing depth | n.a. |
| Antibodies | n.a. |
| Peak calling parameters | n.a. |
| Data quality | n.a. |
| Software | n.a. |

# Flow Cytometry

## Plots

Confirm that:

☒ The axis labels state the marker and fluorochrome used (e.g. CD4-FITC).

☒ The axis scales are clearly visible. Include numbers along axes only for bottom left plot of group (a 'group' is an analysis of identical markers).

☒ All plots are contour plots with outliers or pseudocolor plots.

☒ A numerical value for number of cells or percentage (with statistics) is provided.

## Methodology

| | |
|---|---|
| Sample preparation | Single cell suspensions of lymph nodes or PCLS, CD8/EV co-cultures or MelDCC-lines were incubated for 5 min at 4° C with PBS/10 % AB-serum (Bio-Rad) or human TruStain FcX receptor blocking solution (Biolegend) to reduce non-specific antibody binding, stained with fluorescence-labeled antibodies for 30 min at 4° C and washed once with PBS/2 % FBS/0.01 % NaN3. For intracellular cytokine staining, cells were fixed for 20 min at RT with FluoroFix (BioLegend) and permeabilized with Intracellular Staining Permeabilization Wash Buffer (Biolegend) according to the manufacturer's instructions. Intranuclear staining was conducted with the Foxp3/Transcription Factor Staining Buffer Set (Thermo Fisher) according to the manufacturer's instructions and stained for 30 min. Fixable Viability Dye eFluor 780 (ebioscience) or Zombie NIR Fixable Viability Kit (Biolegend) was used for live/dead cell discrimination. |
| Instrument | Cells were analysed on a LSR II, FACSCelestaTM, FACSymphony™ A5 SORP or Cytoflex (Beckman Coulter) machine. |

| | |
|---|---|
| Software | Data was analyzed with FloJo 10.8.1 (Tree Star). |
| Cell population abundance | Sorting of CD155/CD276 MelDCC lines after CD155/CD276 CRIPSR/Cas9 knock-out was performed with a FACSAria™ IIu cell sorter (BD Bioscience). Purity after post-sorting was determined by FACS. |
| Gating strategy | Control stains (unstained, single stained or FMO controls) were used to set gates. All samples were first FSC-A and SSC-A gated . Gating for total leukocytes was conducted in lymph node samples based on their typical pattern. Subsequent relevant gating was conducted as shown in the gating strategy exemplifying figures (see main figures and extended data). |

☒ Tick this box to confirm that a figure exemplifying the gating strategy is provided in the Supplementary Information.

# Magnetic resonance imaging

## Experimental design

| | |
|---|---|
| Design type | n.a. |
| Design specifications | n.a. |
| Behavioral performance measures | n.a. |

## Acquisition

| | |
|---|---|
| Imaging type(s) | n.a. |
| Field strength | n.a. |
| Sequence & imaging parameters | n.a. |
| Area of acquisition | n.a. |

Diffusion MRI ☐ Used ☒ Not used

## Preprocessing

| | |
|---|---|
| Preprocessing software | n.a. |
| Normalization | n.a. |
| Normalization template | n.a. |
| Noise and artifact removal | n.a. |
| Volume censoring | n.a. |

## Statistical modeling & inference

| | |
|---|---|
| Model type and settings | n.a. |
| Effect(s) tested | n.a. |

Specify type of analysis: ☐ Whole brain ☐ ROI-based ☐ Both

| | |
|---|---|
| Statistic type for inference (See Eklund et al. 2016) | n.a. |
| Correction | n.a. |

## Models & analysis

| n/a | Involved in the study |
|---|---|
| ☒ | ☐ Functional and/or effective connectivity |
| ☒ | ☐ Graph analysis |
| ☒ | ☐ Multivariate modeling or predictive analysis |

| | |
|---|---|
| Functional and/or effective connectivity | n.a. |
| Graph analysis | n.a. |
| Multivariate modeling and predictive analysis | n.a. |
