# Supplementary material for: MCSP+ metastasis founder cells activate immunosuppression early in human melanoma metastatic colonization
Source: Nat Cancer. 2025 May 16;6(6):1017–34. doi: 10.1038/s43018-025-00963-w (PMC12202500; doi:10.1038/s43018-025-00963-w)
Supplement: Supplementary file 11 — Unprocessed western blots for all Figs. 5 and 6 and Extended Data Figs. 7, 9 and 10. [file 43018_2025_963_MOESM11_ESM.pdf]

Fig. 5c

Colorimetric for Marker

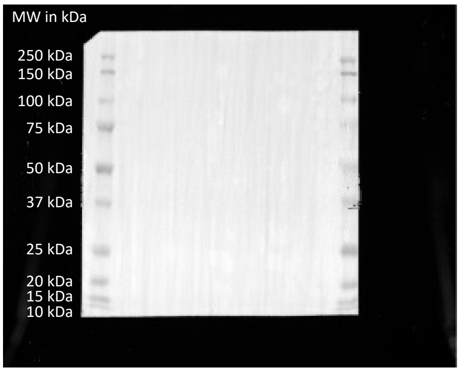

Chemiluminescent

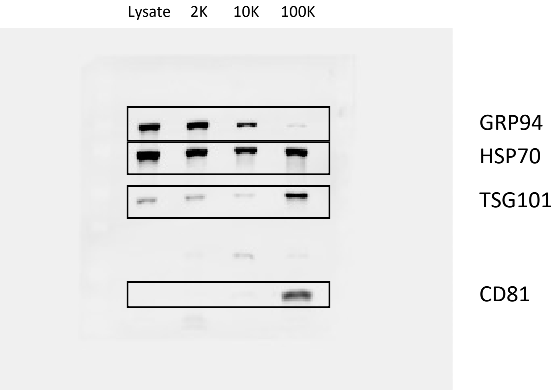

Stain free gel

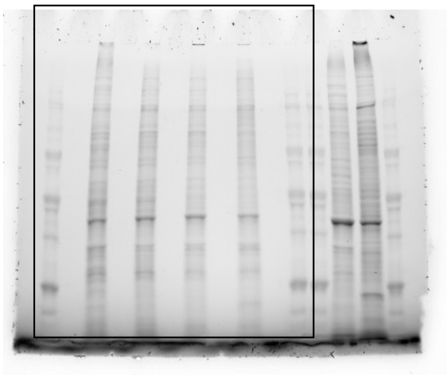

Stain free blot

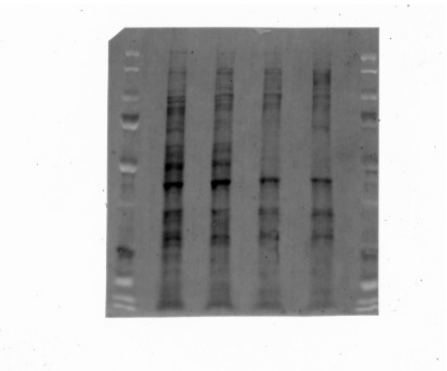

Colorimetric for Marker

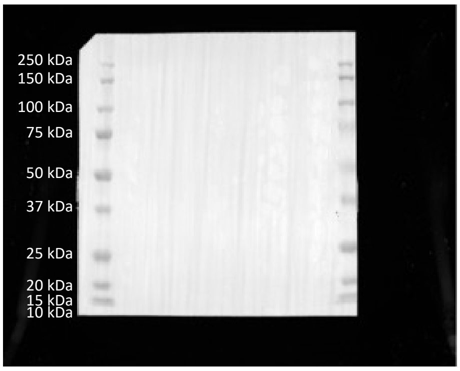

Chemiluminescent

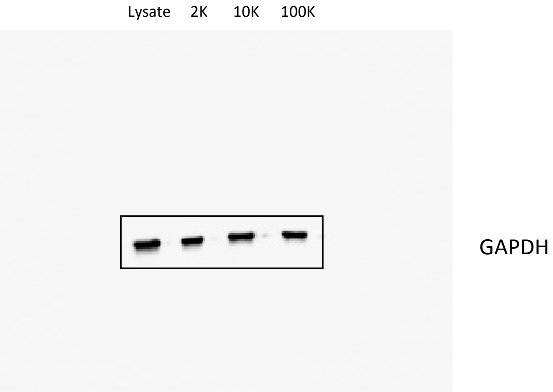

Stain free gel

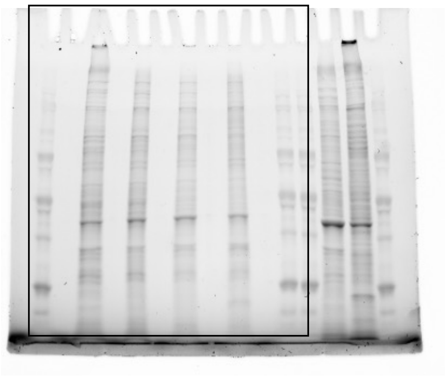

Stain free blot

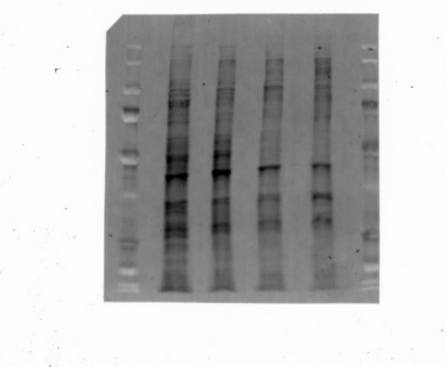

Fig. 6a

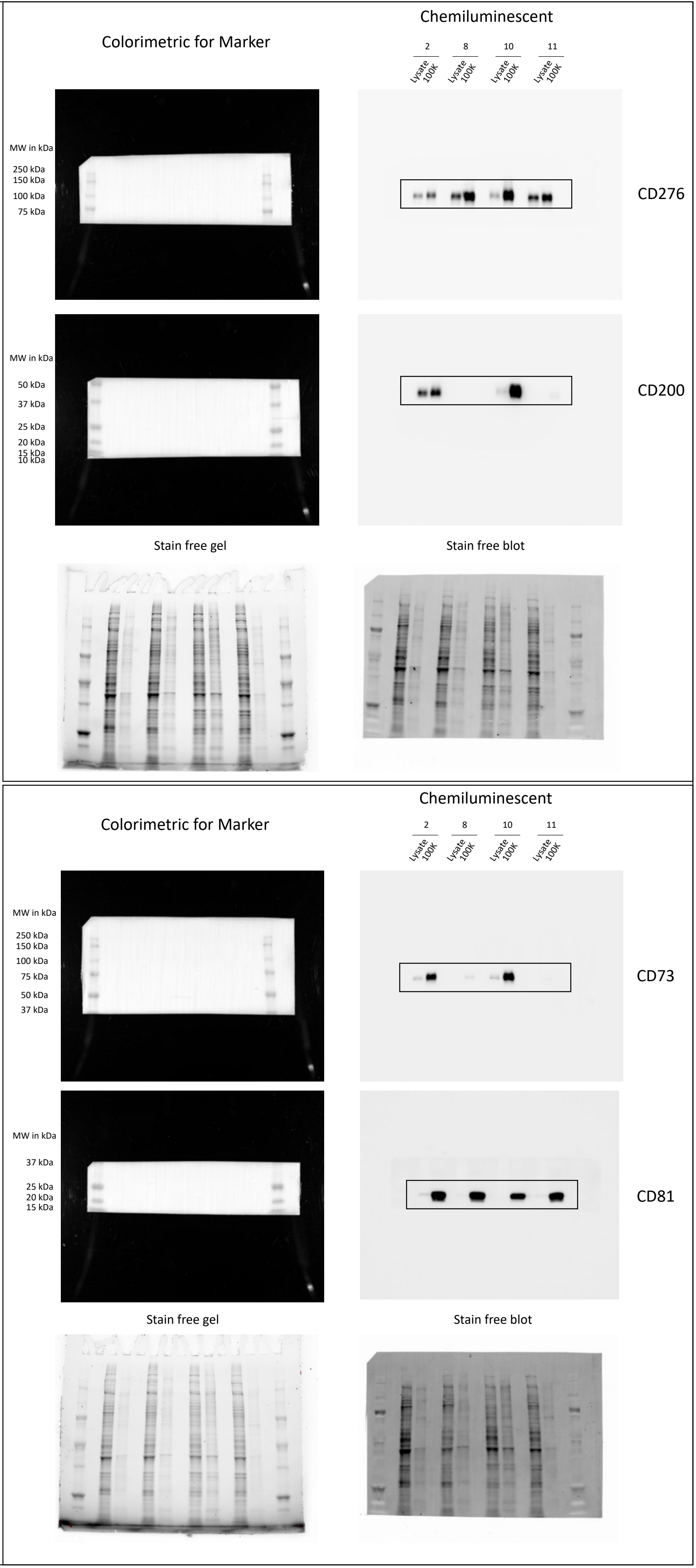

Chemiluminescent
Colorimetric for Marker
2
8
10
11
Lysate
100K
Lysate
100K
Lysate
100K
Lysate
100K
MW in kDa
250 kDa
150 kDa
100 kDa
75 kDa
CD276
MW in kDa
50 kDa
37 kDa
25 kDa
20 kDa
15 kDa
10 kDa
CD200
Stain free gel
Stain free blot
Chemiluminescent
Colorimetric for Marker
2
8
10
11
Lysate
100K
Lysate
100K
Lysate
100K
Lysate
100K
MW in kDa
250 kDa
150 kDa
100 kDa
75 kDa
50 kDa
37 kDa
CD73
MW in kDa
37 kDa
25 kDa
20 kDa
15 kDa
CD81
Stain free gel
Stain free blot

Fig. 6a

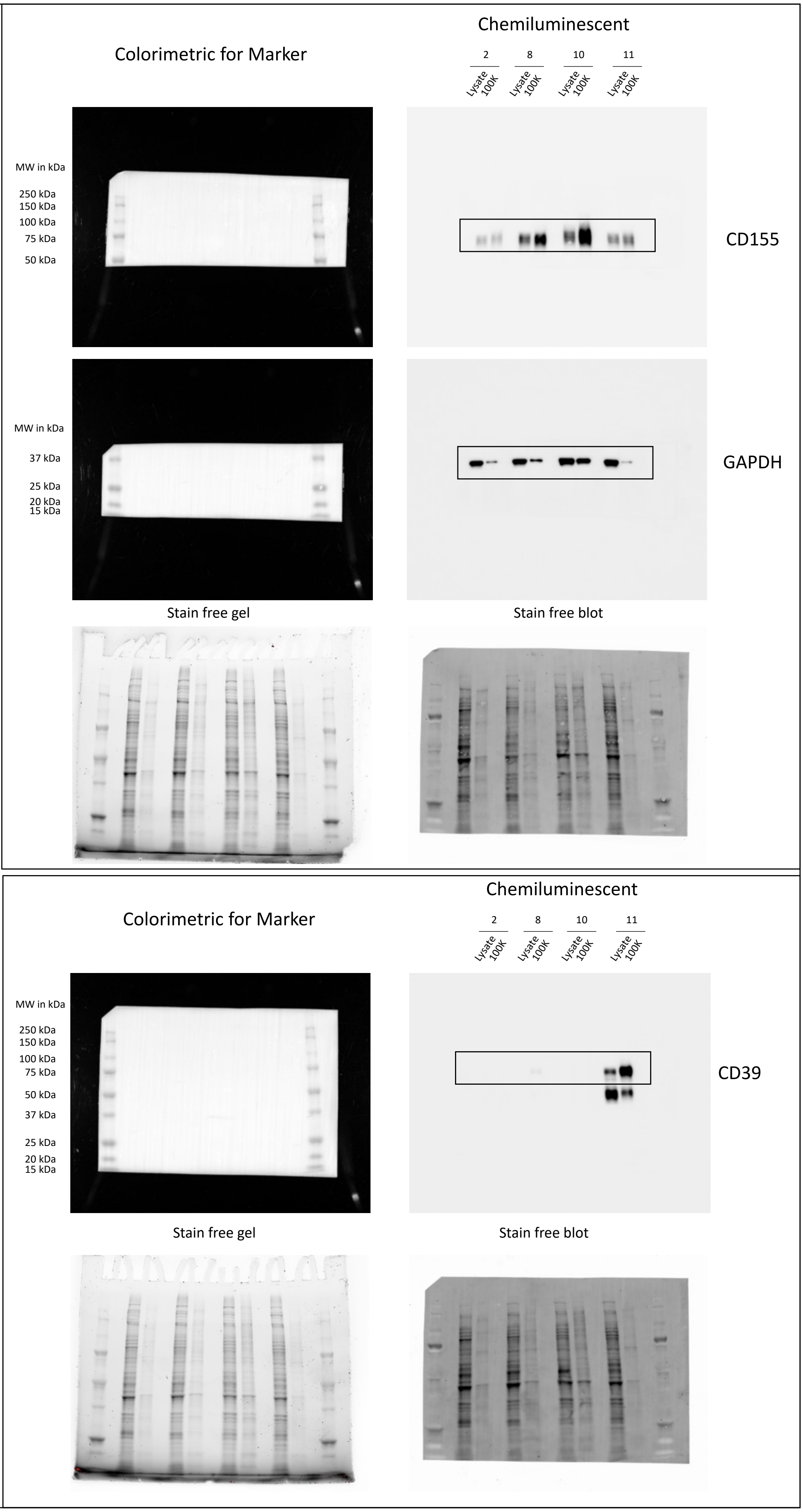

Chemiluminescent
Colorimetric for Marker
2 8 10 11
Lysate 100K Lysate 100K Lysate 100K Lysate 100K
MW in kDa
250 kDa
150 kDa
100 kDa
75 kDa
50 kDa
CD155
MW in kDa
37 kDa
25 kDa
20 kDa
15 kDa
GAPDH
Stain free gel
Stain free blot
Chemiluminescent
Colorimetric for Marker
2 8 10 11
Lysate 100K Lysate 100K Lysate 100K Lysate 100K
MW in kDa
250 kDa
150 kDa
100 kDa
75 kDa
50 kDa
37 kDa
25 kDa
20 kDa
15 kDa
CD39
Stain free gel
Stain free blot

Fig. 6c

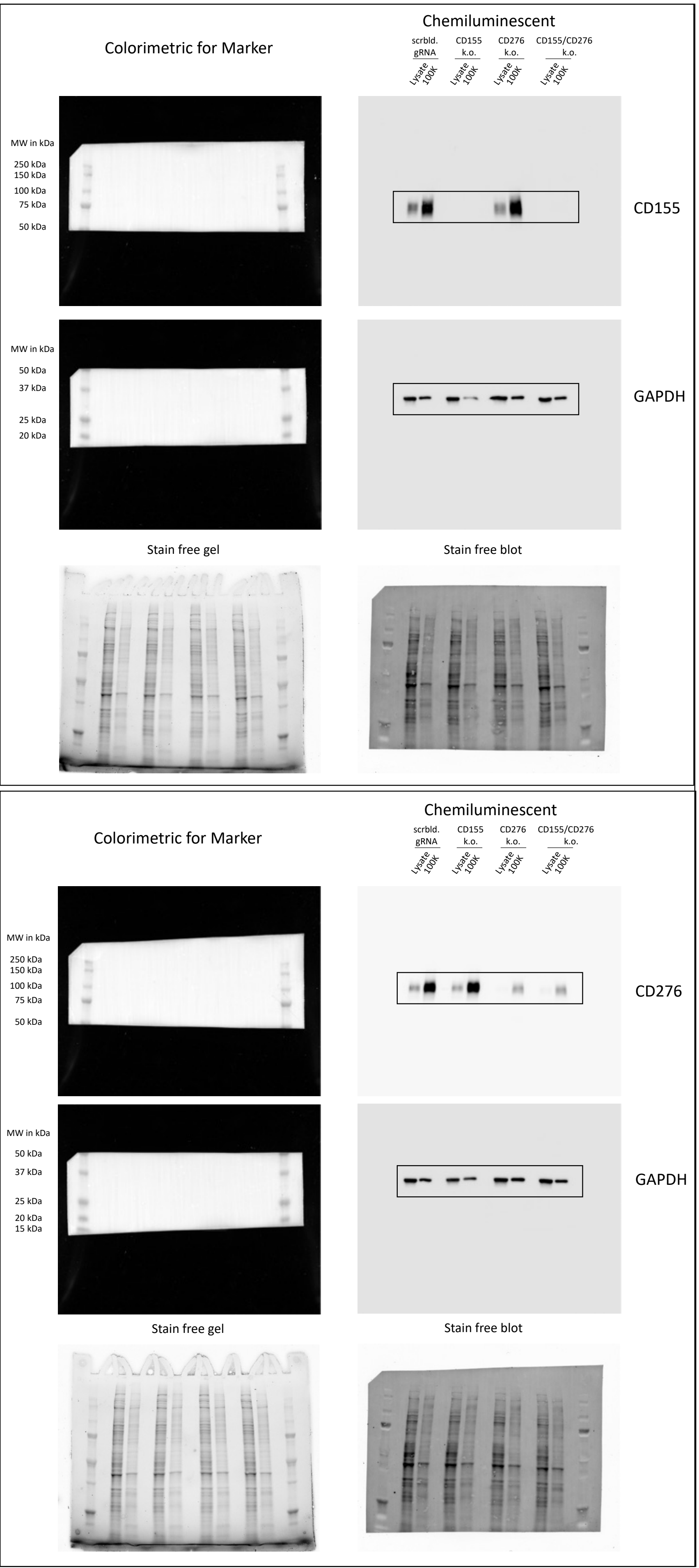

Colorimetric for Marker
Chemiluminescent
scrbld. gRNA
CD155 k.o.
CD276 k.o.
CD155/CD276 k.o.
Lysate
100K
MW in kDa
250 kDa
150 kDa
100 kDa
75 kDa
50 kDa
CD155
MW in kDa
50 kDa
37 kDa
25 kDa
20 kDa
GAPDH
Stain free gel
Stain free blot
Colorimetric for Marker
Chemiluminescent
scrbld. gRNA
CD155 k.o.
CD276 k.o.
CD155/CD276 k.o.
Lysate
100K
MW in kDa
250 kDa
150 kDa
100 kDa
75 kDa
50 kDa
CD276
MW in kDa
50 kDa
37 kDa
25 kDa
20 kDa
15 kDa
GAPDH
Stain free gel
Stain free blot

ED Fig. 7c upper

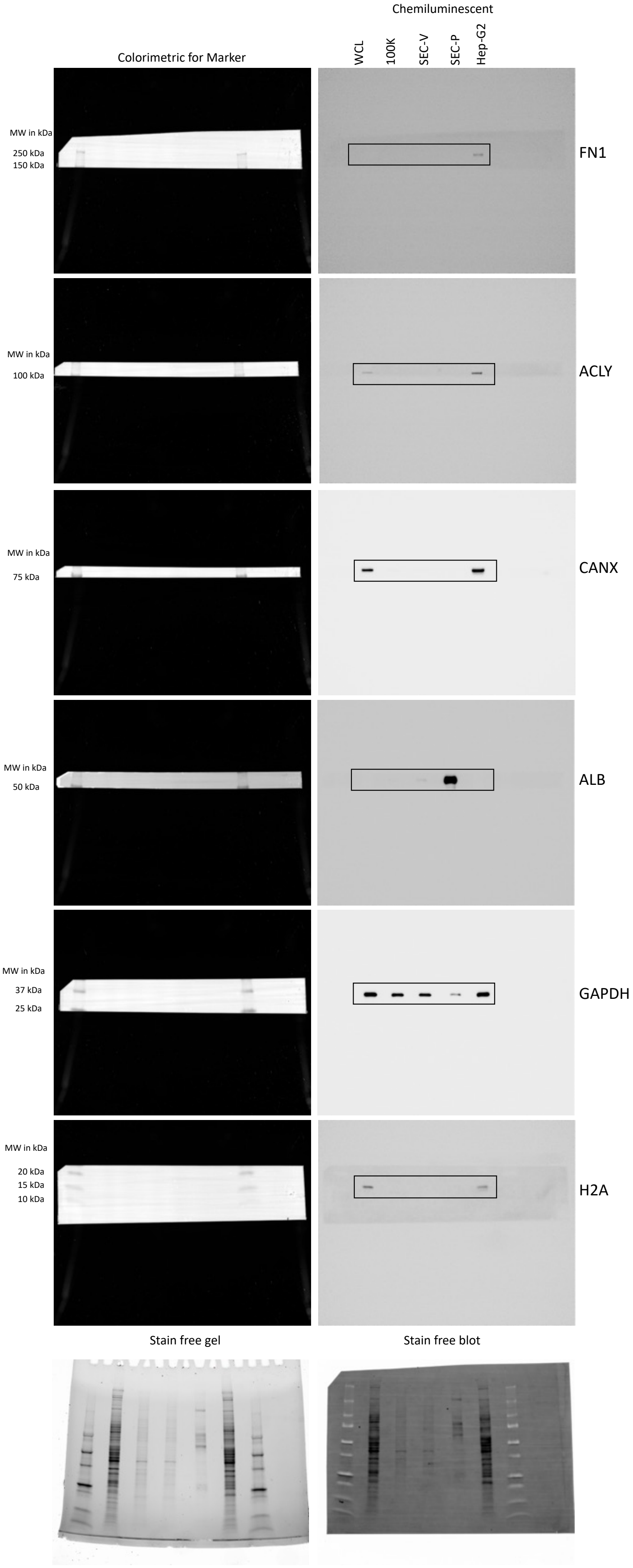

ED Fig. 7c lower

Chemiluminescent

Colorimetric for Marker

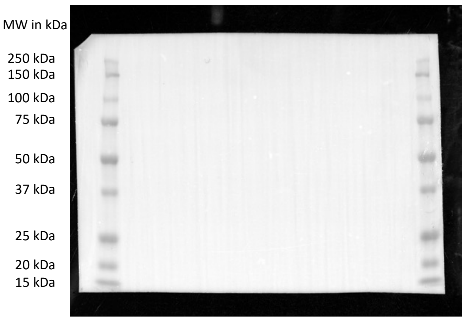

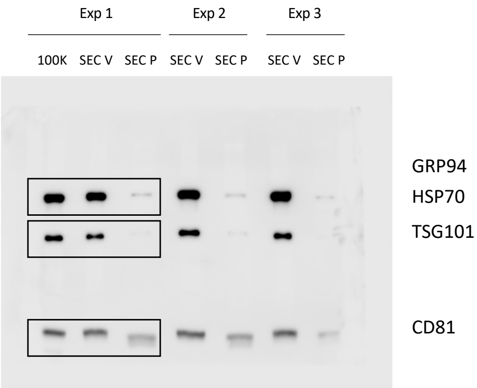

Stain free gel

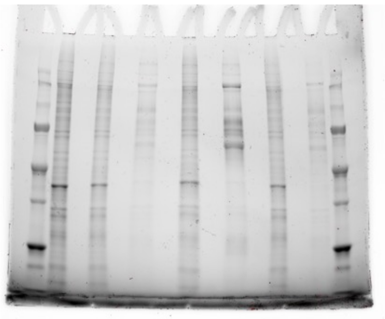

Stain free blot

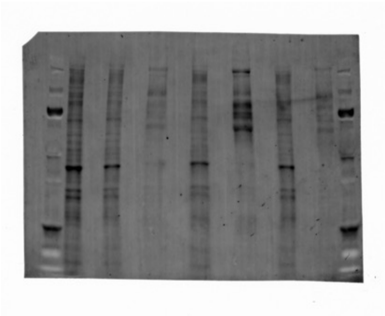

ED Fig. 7f

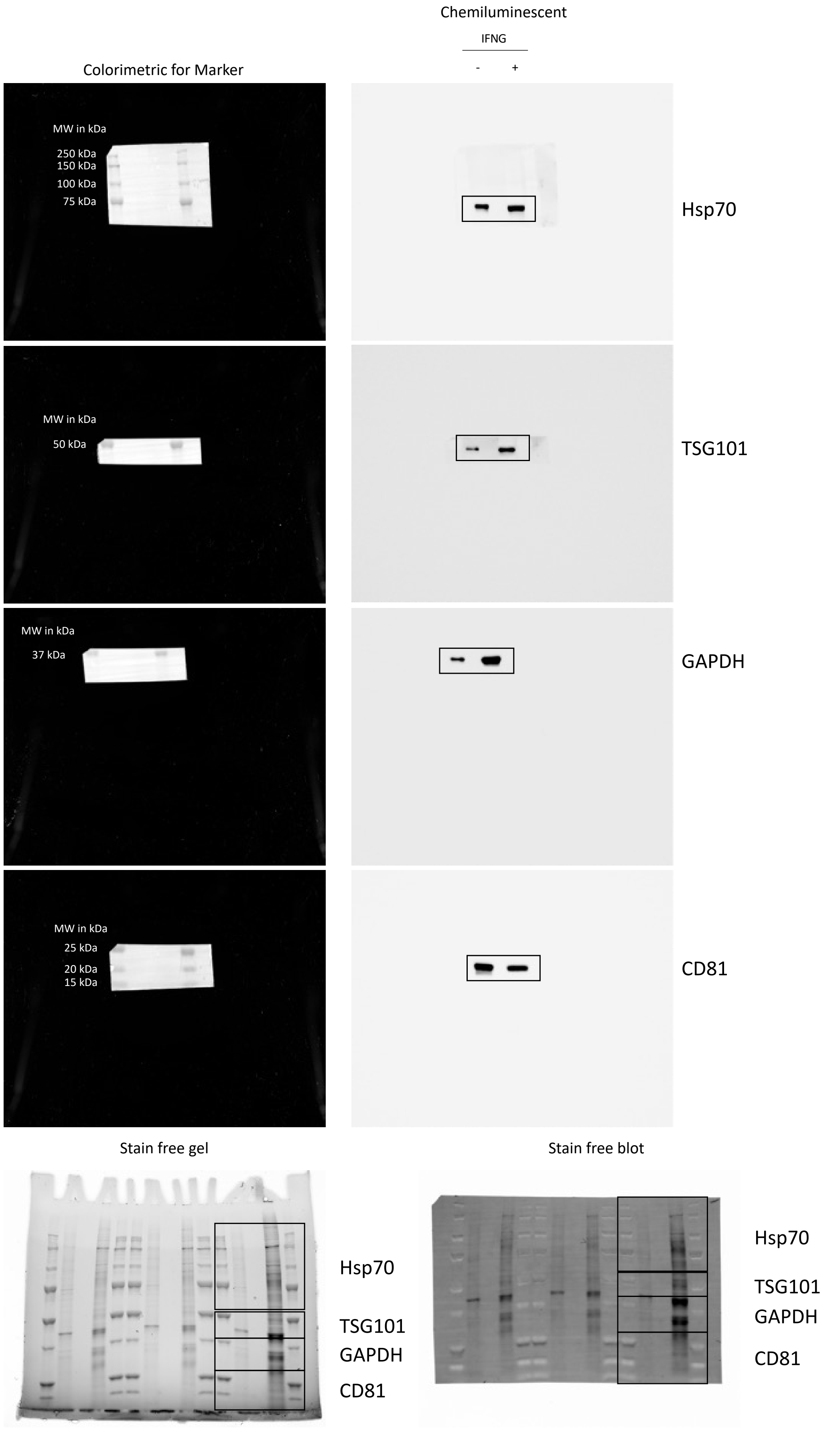
Chemiluminescent
IFNG
- +
Colorimetric for Marker
MW in kDa
250 kDa
150 kDa
100 kDa
75 kDa
Hsp70
MW in kDa
50 kDa
TSG101
MW in kDa
37 kDa
GAPDH
MW in kDa
25 kDa
20 kDa
15 kDa
CD81
Stain free gel
Hsp70
TSG101
GAPDH
CD81
Stain free blot
Hsp70
TSG101
GAPDH
CD81

ED Fig. 9b

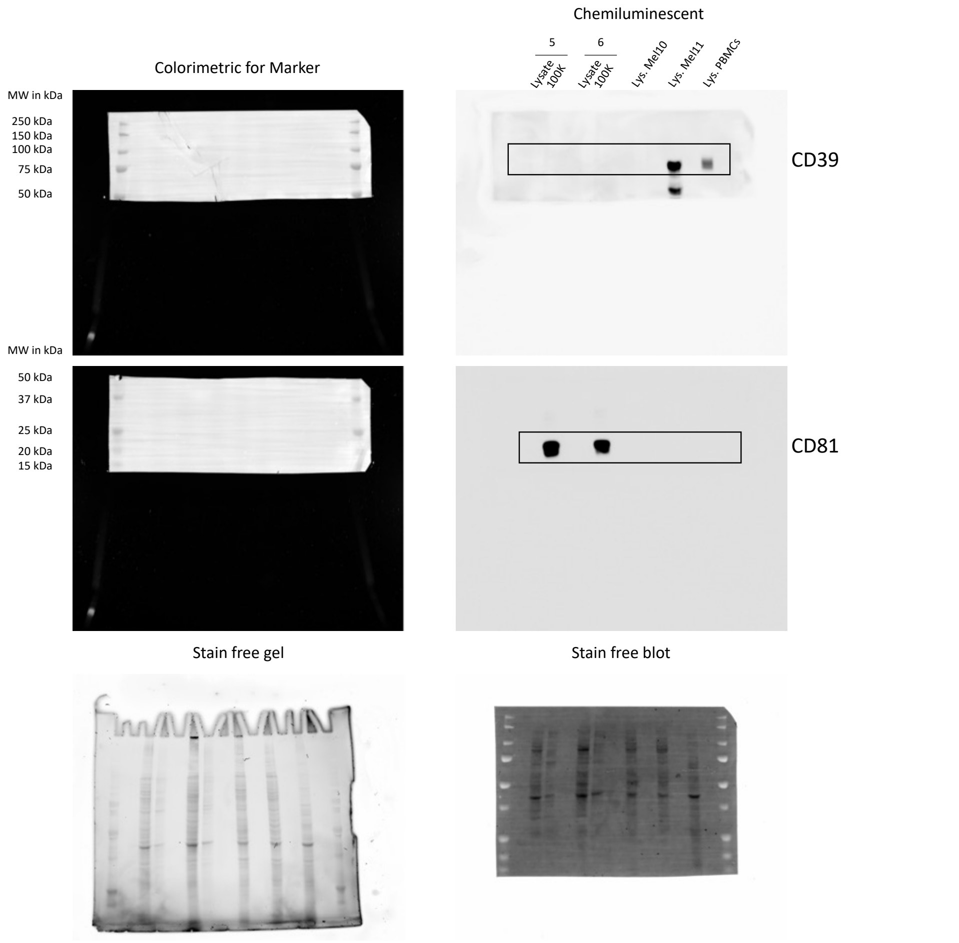
Chemiluminescent
5
6
Lysate 100K
Lysate 100K
Lys. Mel10
Lys. Mel11
Lys. PBMCs
Colorimetric for Marker
MW in kDa
250 kDa
150 kDa
100 kDa
75 kDa
50 kDa
CD39
MW in kDa
50 kDa
37 kDa
25 kDa
20 kDa
15 kDa
CD81
Stain free gel
Stain free blot

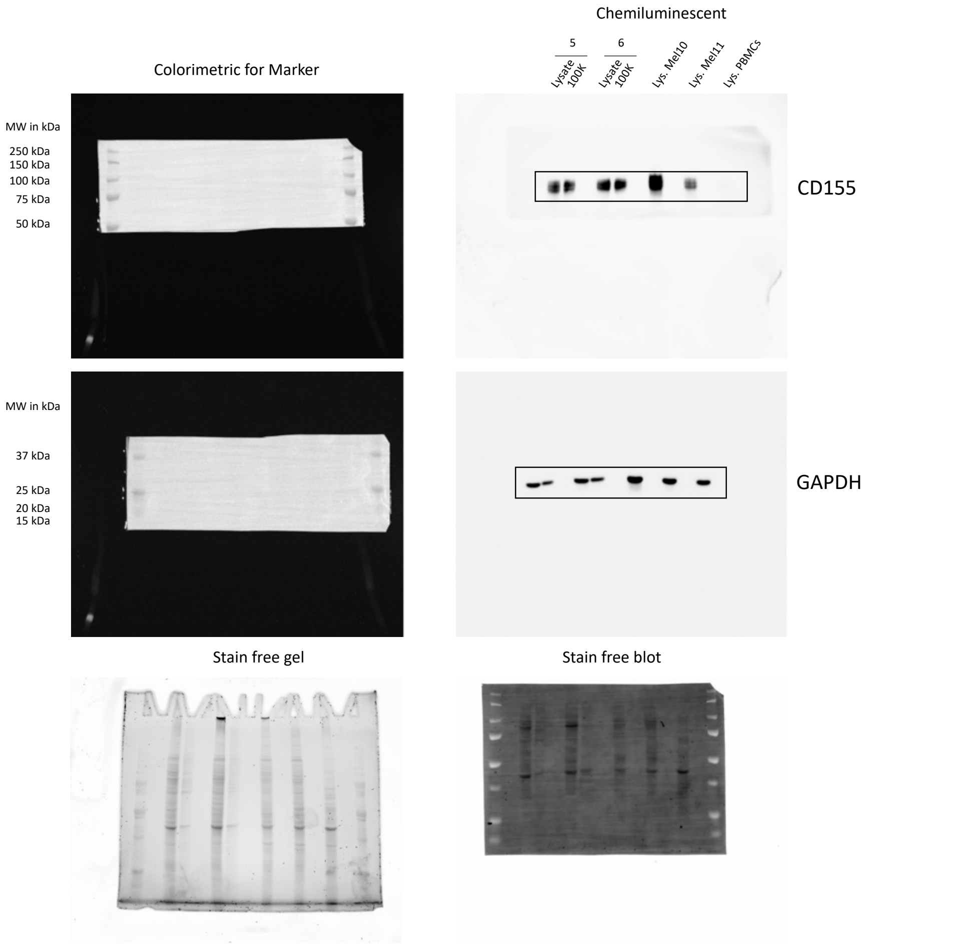
Chemiluminescent
5
6
Lysate 100K
Lysate 100K
Lys. Mel10
Lys. Mel11
Lys. PBMCs
Colorimetric for Marker
MW in kDa
250 kDa
150 kDa
100 kDa
75 kDa
50 kDa
CD155
MW in kDa
37 kDa
25 kDa
20 kDa
15 kDa
GAPDH
Stain free gel
Stain free blot

ED Fig. 9b

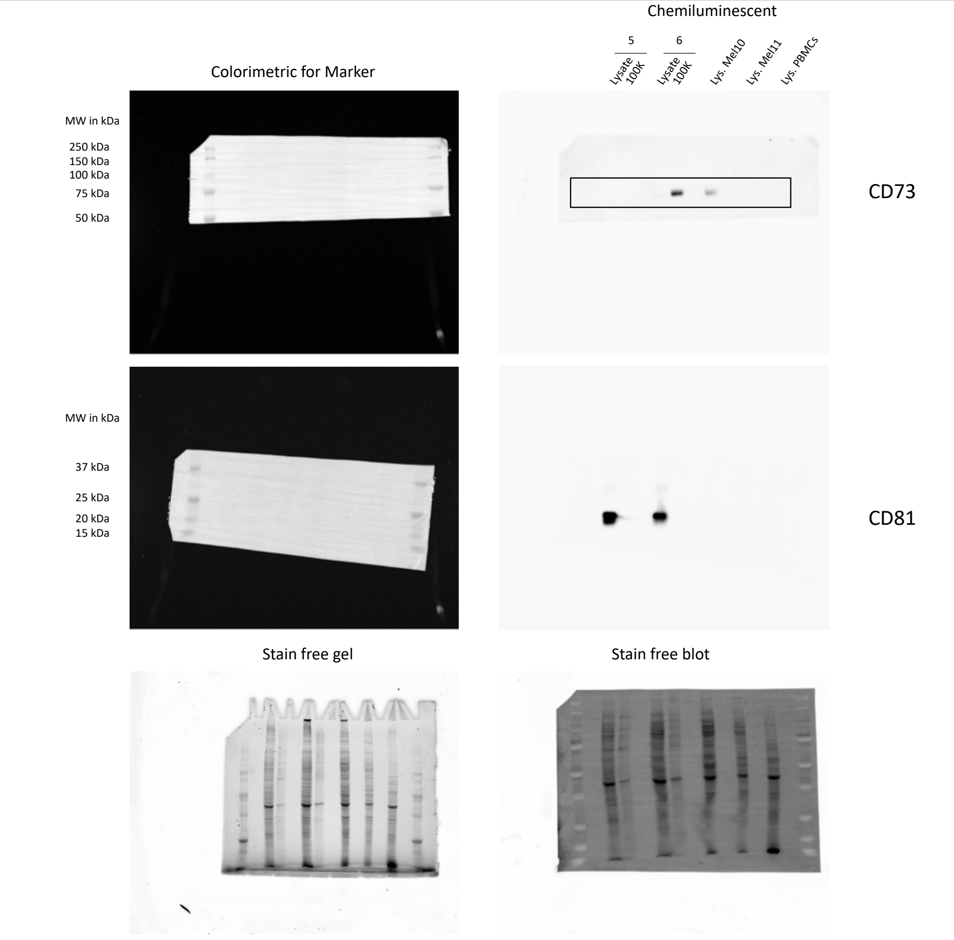
Chemiluminescent
Colorimetric for Marker
5
6
Lysate 100K
Lysate 100K
Lys. Mel10
Lys. Mel11
Lys. PBMCs
MW in kDa
250 kDa
150 kDa
100 kDa
75 kDa
50 kDa
CD73
MW in kDa
37 kDa
25 kDa
20 kDa
15 kDa
CD81
Stain free gel
Stain free blot

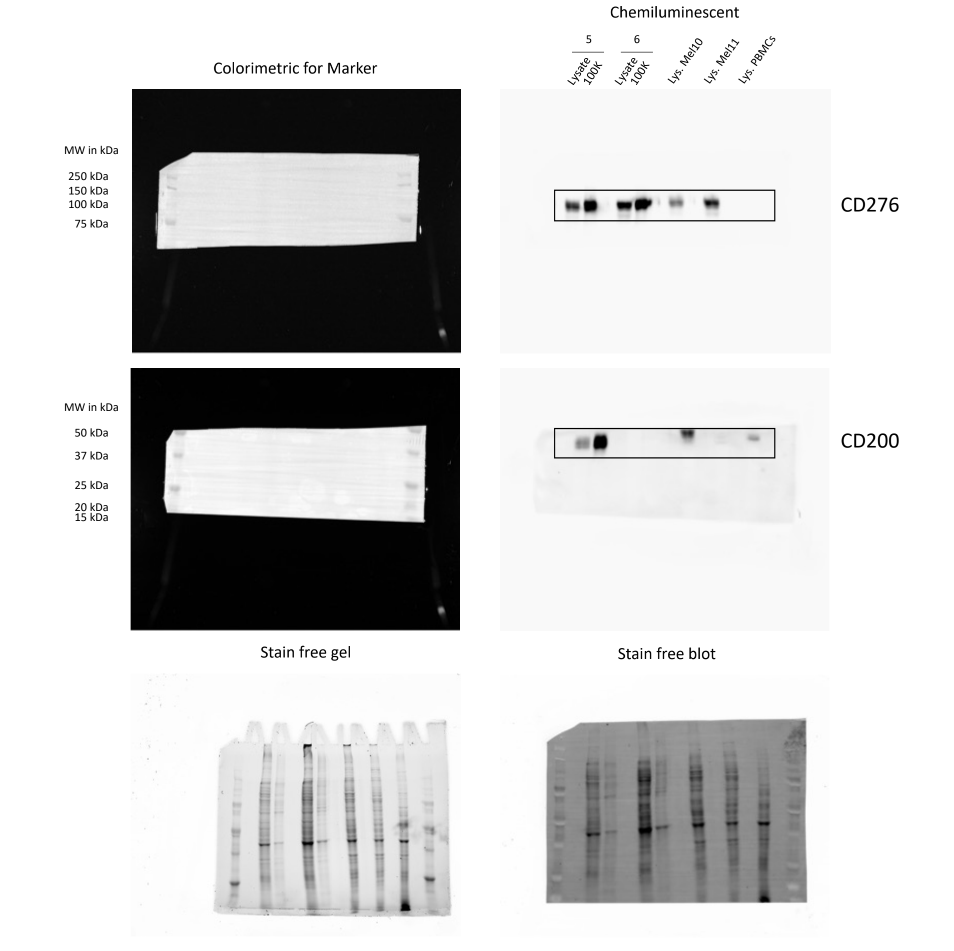
Chemiluminescent
Colorimetric for Marker
5
6
Lysate 100K
Lysate 100K
Lys. Mel10
Lys. Mel11
Lys. PBMCs
MW in kDa
250 kDa
150 kDa
100 kDa
75 kDa
CD276
MW in kDa
50 kDa
37 kDa
25 kDa
20 kDa
15 kDa
CD200
Stain free gel
Stain free blot

ED Fig. 9c

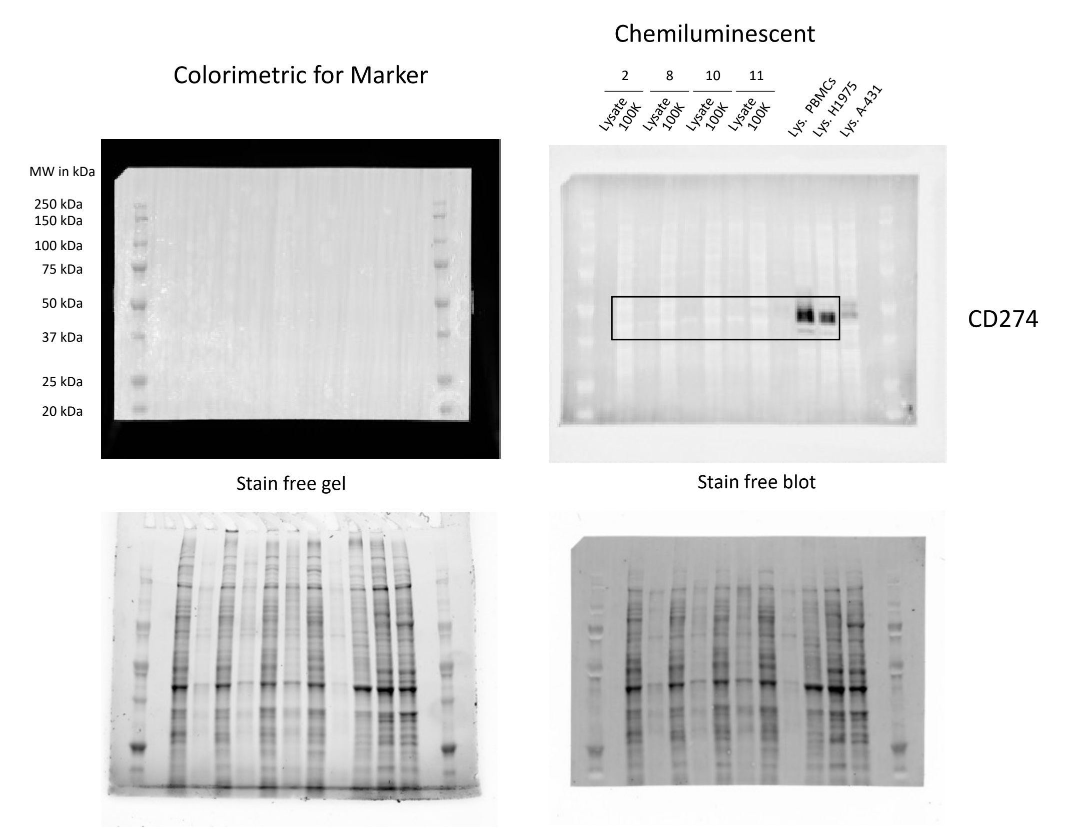
Chemiluminescent
Colorimetric for Marker
2
8
10
11
Lysate
100K
Lysate
100K
Lysate
100K
Lysate
100K
Lys. PBMCs
Lys. H1975
Lys. A-431
MW in kDa
250 kDa
150 kDa
100 kDa
75 kDa
50 kDa
37 kDa
25 kDa
20 kDa
CD274
Stain free gel
Stain free blot

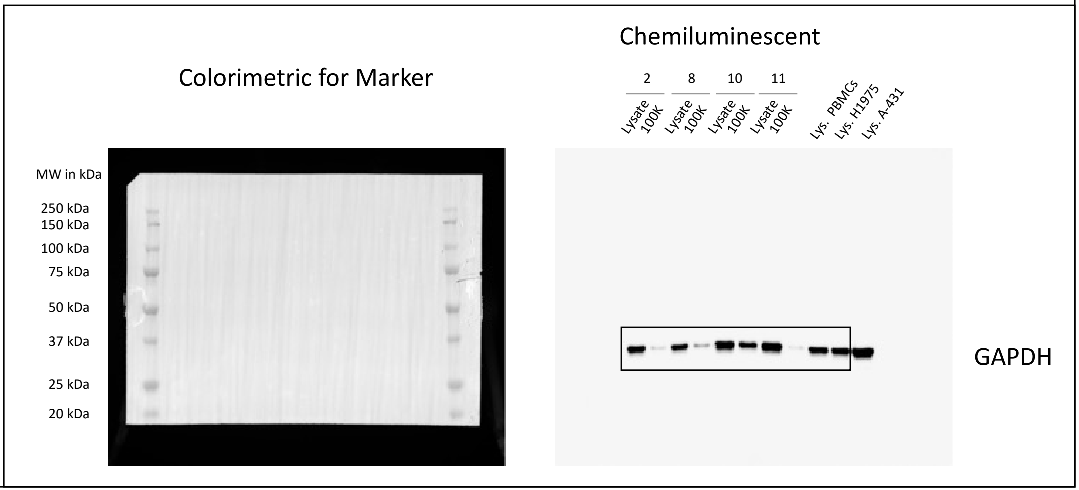
Chemiluminescent
Colorimetric for Marker
2
8
10
11
Lysate
100K
Lysate
100K
Lysate
100K
Lysate
100K
Lys. PBMCs
Lys. H1975
Lys. A-431
MW in kDa
250 kDa
150 kDa
100 kDa
75 kDa
50 kDa
37 kDa
25 kDa
20 kDa
GAPDH

ED Fig. 9d

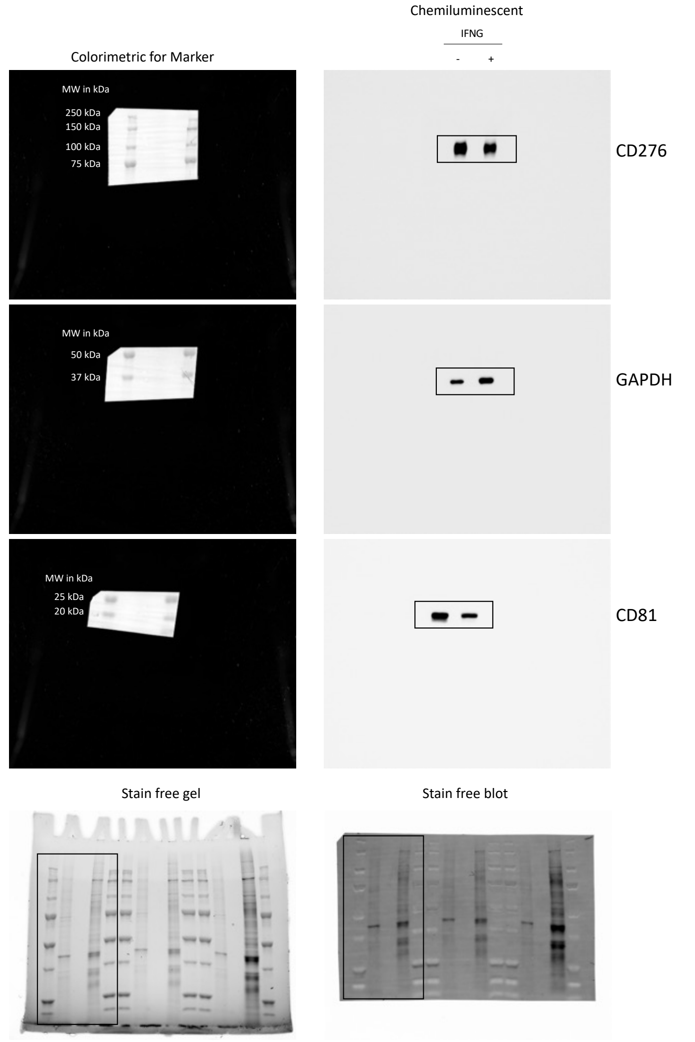
Chemiluminescent
IFNG
- +
Colorimetric for Marker
MW in kDa
250 kDa
150 kDa
100 kDa
75 kDa
CD276
MW in kDa
50 kDa
37 kDa
GAPDH
MW in kDa
25 kDa
20 kDa
CD81
Stain free gel
Stain free blot

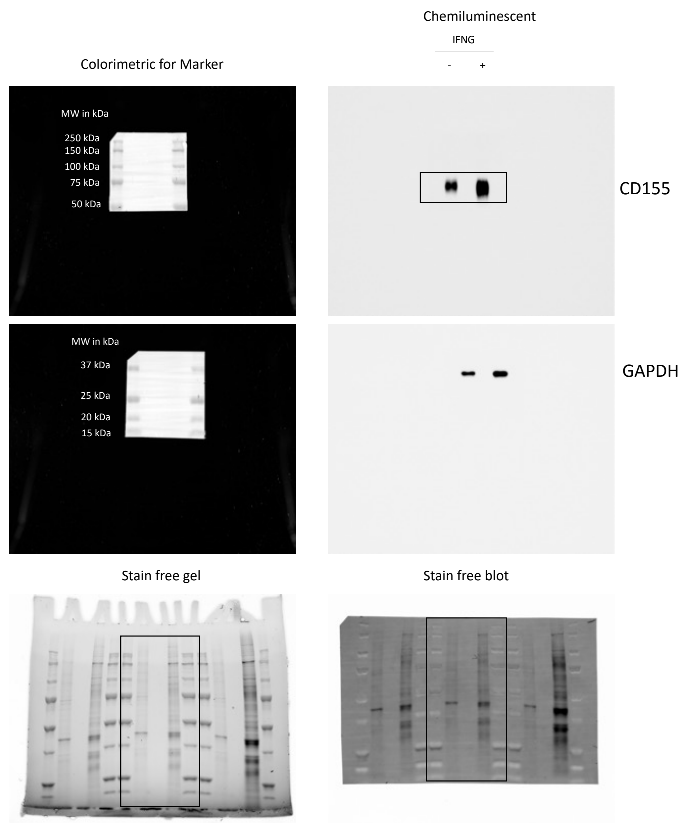
Chemiluminescent
IFNG
- +
Colorimetric for Marker
MW in kDa
250 kDa
150 kDa
100 kDa
75 kDa
50 kDa
CD155
MW in kDa
37 kDa
25 kDa
20 kDa
15 kDa
GAPDH
Stain free gel
Stain free blot

ED Fig. 9h

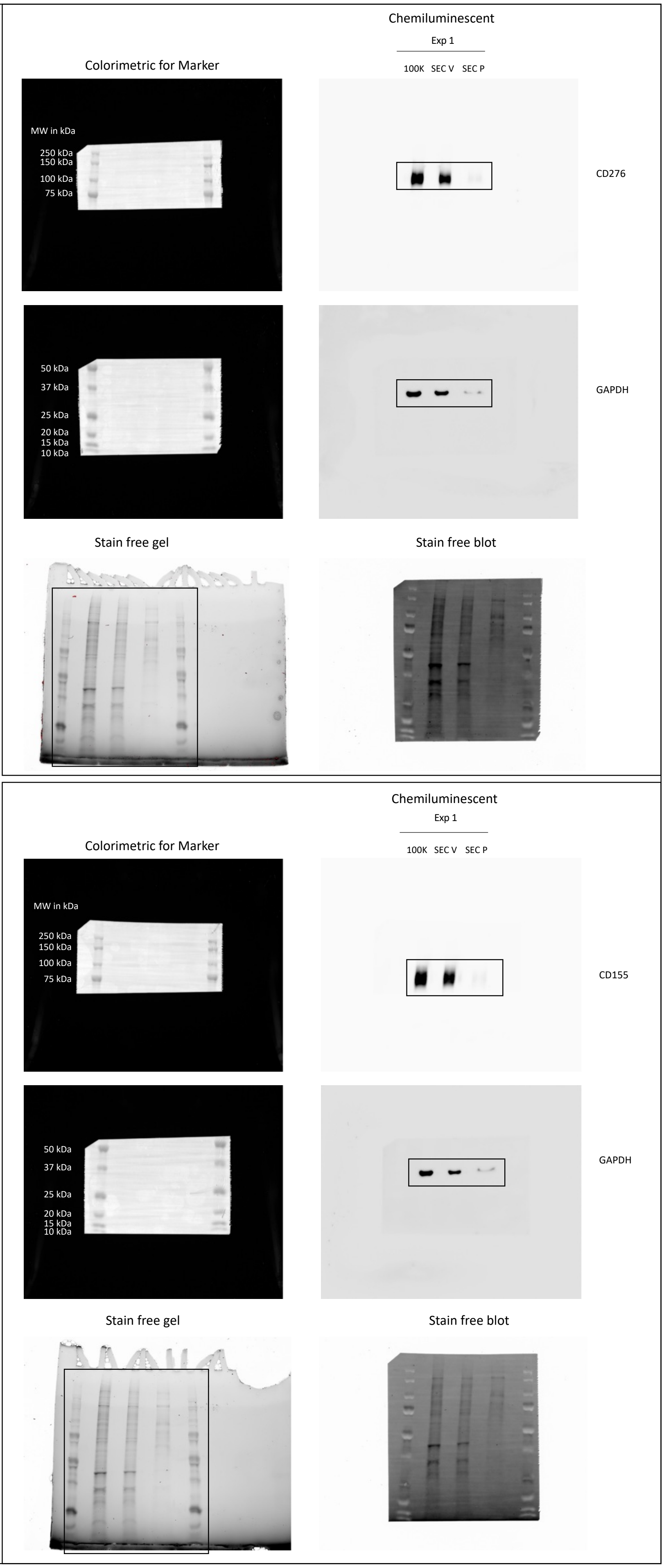
Chemiluminescent
Exp 1
Colorimetric for Marker
100K SEC V SEC P
MW in kDa
250 kDa
150 kDa
100 kDa
75 kDa
CD276
50 kDa
37 kDa
25 kDa
20 kDa
15 kDa
10 kDa
GAPDH
Stain free gel
Stain free blot
Chemiluminescent
Exp 1
Colorimetric for Marker
100K SEC V SEC P
MW in kDa
250 kDa
150 kDa
100 kDa
75 kDa
CD155
50 kDa
37 kDa
25 kDa
20 kDa
15 kDa
10 kDa
GAPDH
Stain free gel
Stain free blot

ED Fig. 9i

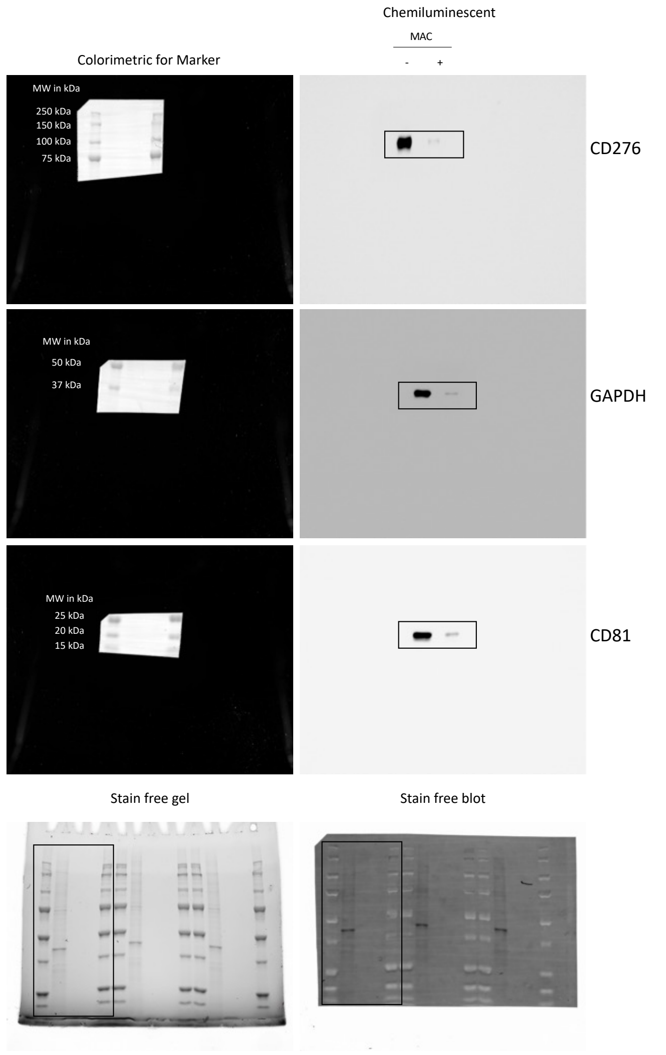
Chemiluminescent
MAC
- +
Colorimetric for Marker
MW in kDa
250 kDa
150 kDa
100 kDa
75 kDa
CD276
MW in kDa
50 kDa
37 kDa
GAPDH
MW in kDa
25 kDa
20 kDa
15 kDa
CD81
Stain free gel
Stain free blot

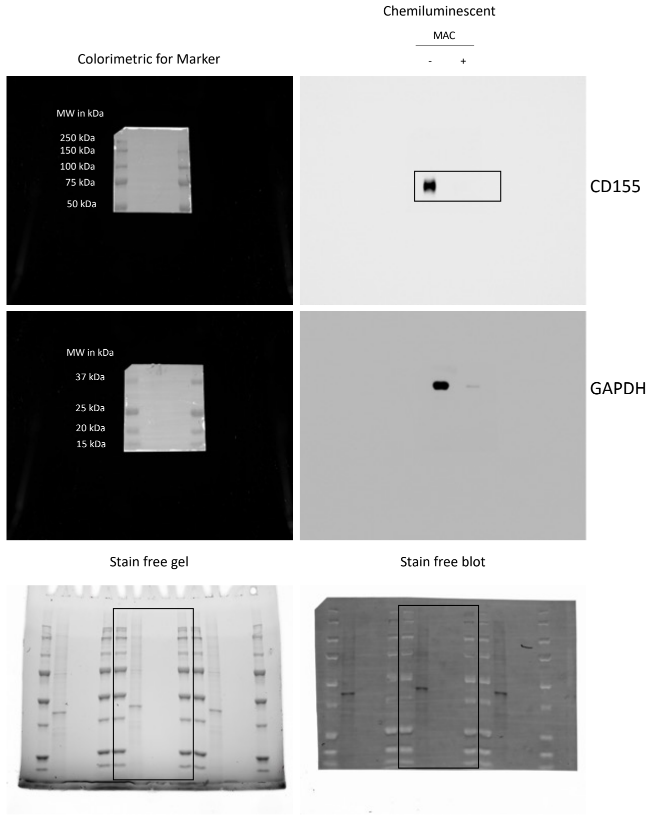
Chemiluminescent
MAC
- +
Colorimetric for Marker
MW in kDa
250 kDa
150 kDa
100 kDa
75 kDa
50 kDa
CD155
MW in kDa
37 kDa
25 kDa
20 kDa
15 kDa
GAPDH
Stain free gel
Stain free blot

ED Fig. 9l upper

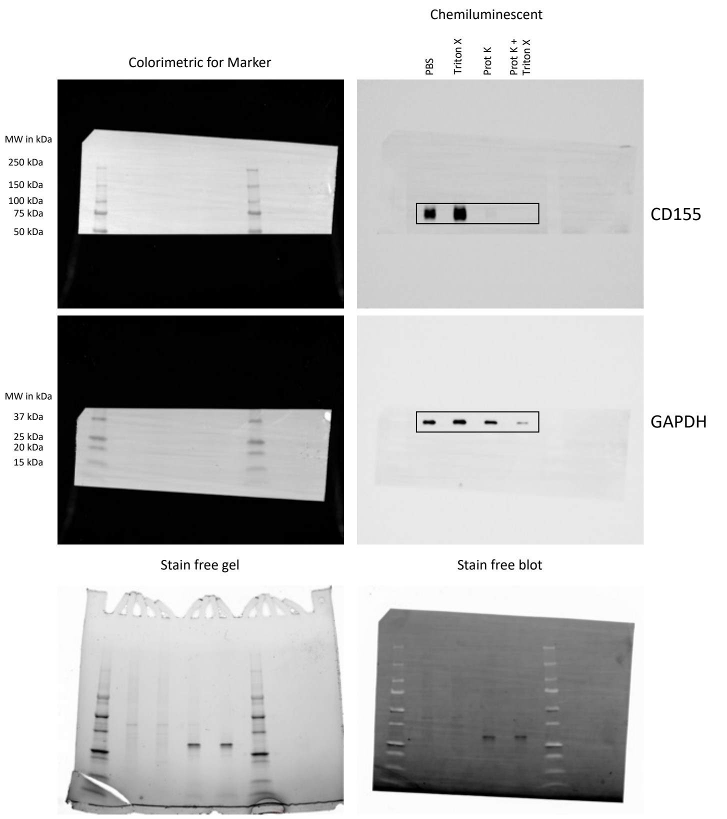

ED Fig. 9l lower

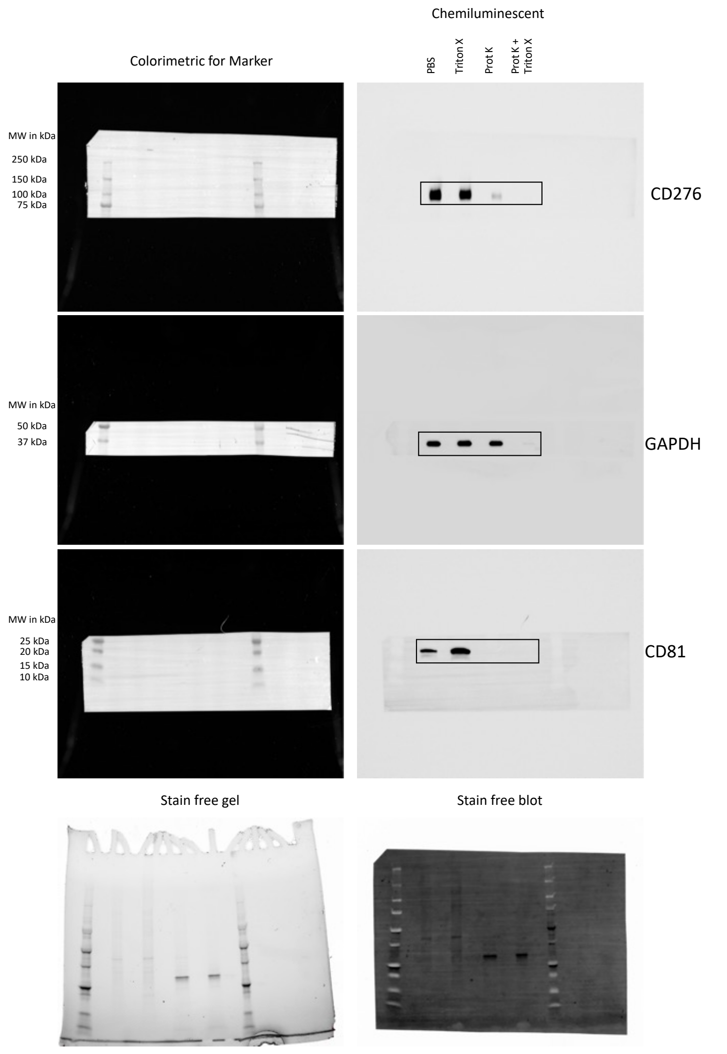

ED Fig. 10a

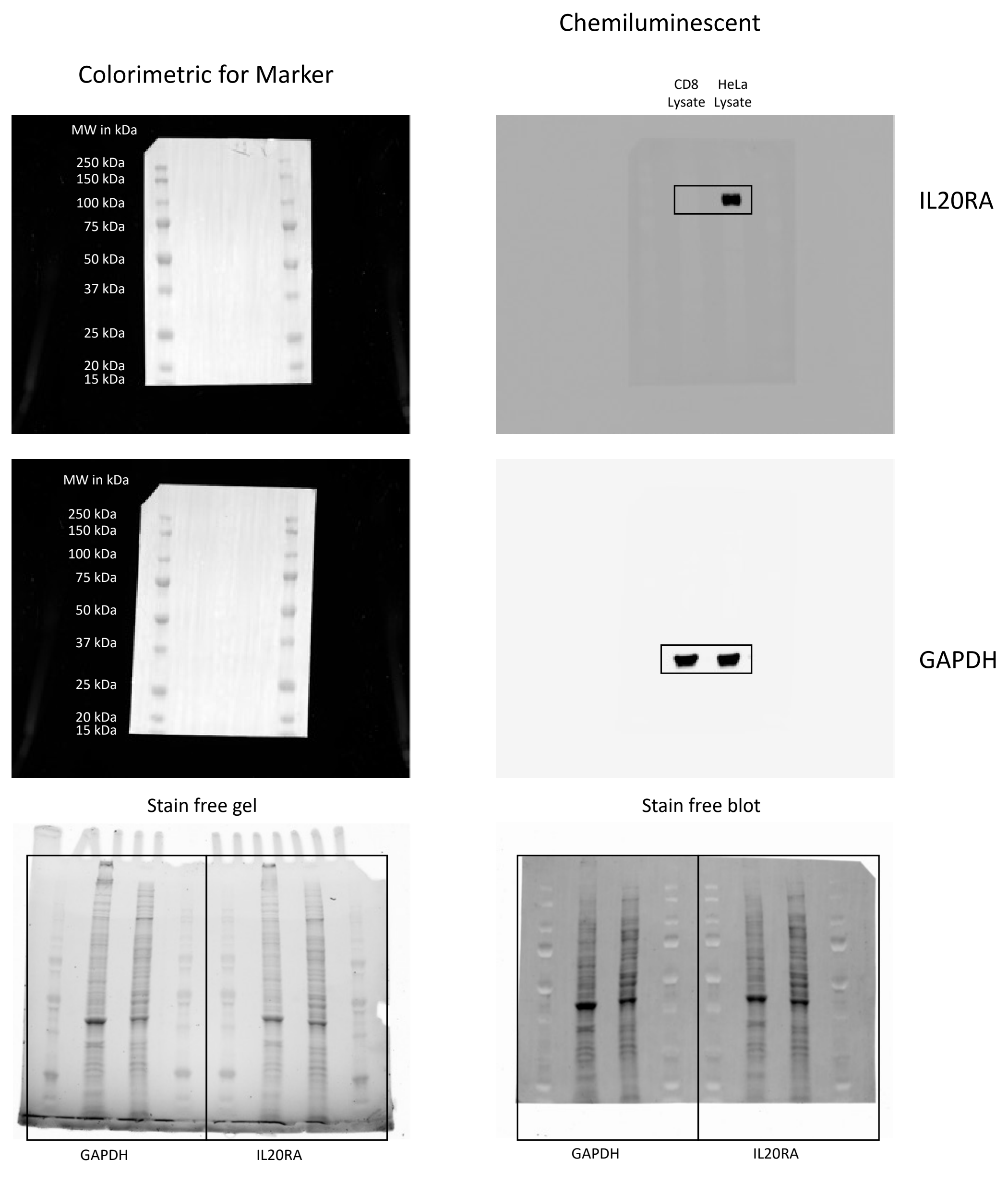
Chemiluminescent
Colorimetric for Marker
MW in kDa
250 kDa
150 kDa
100 kDa
75 kDa
50 kDa
37 kDa
25 kDa
20 kDa
15 kDa
CD8 Lysate
HeLa Lysate
IL20RA
MW in kDa
250 kDa
150 kDa
100 kDa
75 kDa
50 kDa
37 kDa
25 kDa
20 kDa
15 kDa
GAPDH
Stain free gel
Stain free blot
GAPDH
IL20RA
GAPDH
IL20RA
